# Design, Synthesis, and Biological Evaluation of Pseudo‐Natural Products Inspired by Aryloctahydroindole Alkaloids

**DOI:** 10.1002/cmdc.202501102

**Published:** 2026-02-12

**Authors:** Luca C. Greiner, Freddy A. Bernal, Sasikala Thavam, Maite Brachthäuser, Sonja Sievers, Slava Ziegler, Herbert Waldmann

**Affiliations:** ^1^ Abteilung Chemische Biologie Max‐Planck‐Institute für Molekulare Physiologie Dortmund Germany; ^2^ Compound Management and Screening Center Dortmund Germany; ^3^ Fakultät Chemie und Chemische Biologie Technische Universität Dortmund Dortmund Germany

**Keywords:** cell painting seahorse flux, mesembrine, mitochondrial complex, pseudo‐natural products

## Abstract

We report the design, synthesis, and biological characterization of a pseudo‐NP (PNP) collection inspired by the mesembrine alkaloid scaffold. A scalable, microwave‐assisted intramolecular Diels–Alder furan cyclization enabled efficient access to the 3a‐aryloctahydroindole (AOHI) core, which served as a versatile intermediate for diversification through NP‐fragment fusion. The resulting AOHI‐based PNPs explore chemical space bridging properties of NPs and drug‐like molecules. Morphological and bioenergetic profiling revealed distinct mitochondrial phenotypes, including altered morphological features and respiration consistent with complex I perturbation. These findings highlight the AOHI scaffold as a versatile and synthetically accessible alkaloid‐derived fragment for PNP design and as a valuable source for novel biologically active compound classes.

## Graphical abstract

Mesembrine alkaloid pseudo‐natural products were synthesized employing a microwave‐assisted Diels–Alder reaction as a key step. Morphological profiling by cell painting revealed compound‐topology‐dependent mitochondrial fragmentation or inhibition of respiration.

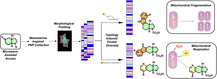

## 1 Introduction

Bioactive natural products (NPs) define the fraction of biologically relevant chemical space explored by nature in evolution and have inspired small‐molecule design in chemical biology and drug discovery [1]. Their scaffolds are biologically validated, but evolution is slow, and biosynthetic constraints limit the exploration of natural product‐related chemical space [2, 3]. This limitation can be addressed by the pseudo‐natural product (PNP) concept [4, 5, 6, 7, 8, 9, 10, 11, 12]. In PNPs, NP fragments are combined in arrangements not accessible by current biosynthesis pathways. Their scaffolds do not occur in nature, yet they retain the biological relevance of NPs and are enriched in unusual or unexpected bioactivity [13, 14, 15, 16, 17, 18, 19, 20, 21, 22, 23].

**FIGURE 1:**
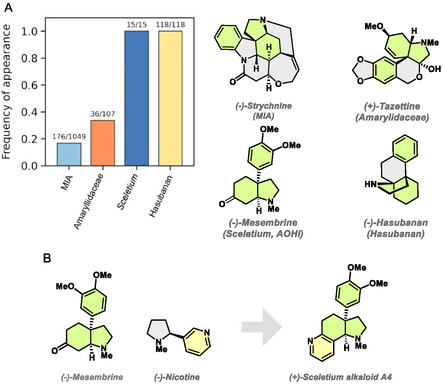
PNP design strategy. (A) Occurrence of the 3a‐aryloctahydroindole (AOHI) motif in alkaloid NPs. Enrichment comparison based on 1,049 monoterpene indole alkaloids (MIAs), 107 Amaryllidaceae alkaloids, 15 *Sceletium* alkaloids, and 118 Hasubanan alkaloids obtained from the ChEMBL and COCONUT databases. (B) Angular edge of a pyridine and an AOHI‐fragment in the alkaloid Sceletium A4.

For PNP design, the combination of fragments that frequently occur in NPs with different bioactivity and biosynthetic origin with unrelated NP‐fragments, preferably in chemical complexity‐generating transformations, may be particularly relevant. Such PNP classes may populate regions of biologically relevant chemical space defined by the guiding NPs but display differing bioactivity. The 3a‐aryloctahydroindole (AOHI) motif occurs in >300 alkaloids with diverse bioactivity and spans various alkaloid classes with differing biosynthetic origin, e.g., *Sceletium*‐ [24], Amaryllidaceae‐ [25], Hasubanan‐ [26], and monoterpene indole alkaloids (MIAs) [27] (Figure 1A). Prominent representative NPs of these alkaloid classes, which exemplify the diverse structural complexity in the presence of the AOHI motif, are (‐)‐strychnine, (+)‐tazettine, (‐)‐mesembrine, and (‐)‐Hasubanan. Notably, the AOHI‐motif defines the scaffold of the alkaloid mesembrine, a serotonine transport inhibitor, and a major alkaloidal constituent of *Sceletium tortuosum* extracts (Zembrin) (Figure 1A) [28, 29]. Fusion with a pyridine ring yields Sceletium A4 (Figure 1B), a neuroactive alkaloid from *Sceletium tortuosum* without an identified molecular target [30, 31]. This finding, and the occurrence of the AOHI motif in different alkaloid classes in general, suggest that a combination of the AOHI‐fragment with different NP‐derived heterocycles might yield novel PNP‐classes with unexpected bioactivity.

Here, we describe the design and synthesis of PNPs in which the AOHI fragment of mesembrine is combined in different arrangements with alkaloid‐derived heterocyclic fragments, characteristic of widely occurring alkaloid classes. Biological investigation in the morphological cell painting assay (CPA) revealed that the PNPs are enriched in bioactivity not shared by the guiding mesembrine, with notable activity in the modulation of mitochondrial function.

## 2 Results and Discussion

### 2.1 PNP Synthesis

**SCHEME 1:**
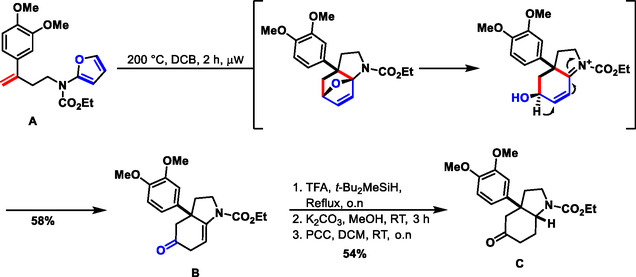
Microwave‐assisted intramolecular Diels–Alder route toward the AOHI fragment. DCB: 1,2‐dichlorobenzene.

**SCHEME 2:**
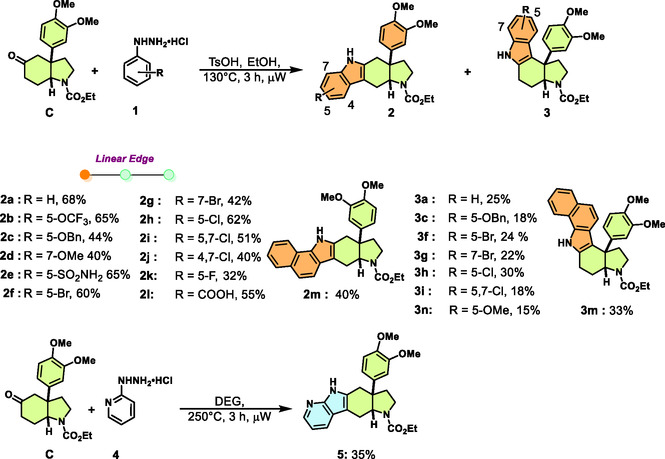
Microwave‐assisted Fischer indole synthesis affords PNPs with angular and linear edge fusions.

For the synthesis of the compound collection, we envisaged employing *γ*‐amino ketone **C** (Scheme 1) as a central intermediate. Mesembrine embodies a characteristic dimethoxy aryl‐substituent, which was also kept constant in our investigations [32]. The *γ*‐amino ketone structure was chosen since the keto group is a versatile handle for the introduction of different heterocycles and because [33, 34, 35, 36, 37], as opposed to the *β*‐amino ketone structure in mesembrine, it is not subject to retro‐Mannich and retro‐aza Michael reactions, facilitating synthesis. We drew from previous work by Padwa et al. [38] and Wipf et al. [39]. These authors had shown that the hexahydroindole core can efficiently be accessed by means of an intramolecular Diels–Alder reaction employing a furan as diene, and that these cycloadditions can be accelerated by microwave irradiation. Accordingly, the synthesis of the desired *α*‐amino‐substituted furan **A** is described in the Supporting Information (Table S1). **A** was exposed to microwave irradiation at elevated temperatures. Optimization of the reaction conditions (see the Supporting Information, Table S1) revealed that irradiation for 2 h at 200°C in 1,2‐dichlorobenzene was most advantageous. After the formation of the initial oxacyclic adduct and nitrogen‐assisted ring opening, a cascade terminating in a vinylogous aza‐pinacol rearrangement yielded intermediate ketone **B** in 58% yield, and on a multigram scale 51% (Scheme 1) [38, 40]. Subsequent simultaneous ionic reduction of the olefin and the ketone, saponification of the intermediary formed trifluoroacetic acid ester, and reoxidation of the liberated secondary alcohol yielded the desired ketone **C** in 54% overall yield. By analogy to the observations by Padwa et al. [38], the final product was formed as a single *cis*‐isomer. Ketone **C** was then subjected to Fischer‐indole syntheses (FIS) under microwave irradiation to fuse an indole ring, characteristic of numerous, widely occurring indole alkaloids, to the mesembrine‐derived fragment in linear or angular edge‐on connectivity (Scheme 2). Exposure of *cis*‐fuzed ketone **C** to various phenylhydrazines under FIS conditions exhibited reversed regioselectivity compared to the findings of Christoffers et al. [41, 42] While Christoffers et al. reported that *cis*‐fuzed systems yield angular indole products. when utilizing *γ*−quaternary ketones, *β*−quaternary ketone **C** primarily yielded linear annulated derivatives **2** as the major isomers, accompanied by minor angular fusion products **3**. This reversal of regioselectivity likely arises from energetically disfavored interactions between the conformationally restricted and sterically congested bridgehead environment and the orientation of aryl groups of the corresponding ene‐hydrazine [41, 42]. Moreover, the presence of vicinally fuzed sp^2^ centers (ene‐hydrazine or indole) further increases strain around the bridgehead segment, disfavoring the angular annulation pathway toward isomer **3.**

**SCHEME 3:**
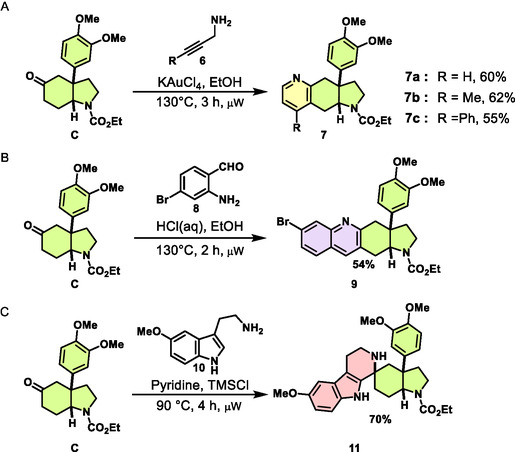
Synthesis of PNPs library through diverse fragment combination reactions. (A) Gold‐catalyzed annulation yields fuzed pyridines. (B) Friedländer synthesis yields a fuzed quinoline. (C) Pictet–Spengler reaction yields fuzed tetrahydrocarboline.

Interestingly, phenylhydrazines with electron‐withdrawing *para*‐substituents like ‐OCF_3_, ‐SO_2_NH_2_, and ‐COOH generally react slower, allowing the system to approach thermodynamic control in the regioselectivity‐determining ene‐hydrazine‐forming step of the FIS [43], resulting in linearly fuzed indole products **2b**, **2e**, and **2l**. For the same reason, when utilizing inherently electron‐deficient heteroaromatics such as pyridyl hydrazine, we exclusively observed the linear 7‐azaindole PNP **5**, which formed only at elevated reaction temperatures. Geometrical factors, such as the *ortho* positioning of methoxy groups on the phenylhydrazine, can hinder ene‐hydrazine orientation, resulting only in the observation of the 7‐substituted linear fusion product **2d**. Additionally, a second chloride may cause steric repulsion with the veratrole group, favoring the formation of linear product **2j**. Chloro‐ and bromo‐substituted analogs readily afforded angular products **3i** and **3g**, whereas the sterically more demanding methoxy‐substituted derivative yielded the linear isomer **2d**. Unsubstituted and monosubstituted halogen‐bearing derivatives afforded product mixtures of **2** and **3** due to their competing +M resonance donation counteracting the dominant ‐I withdrawal, whereas ‐OCF_3_, with its disproportionately stronger ‐I effect and much weaker +M contribution, induced selective formation of the linear product **2b**. Notably, the *p*‐methoxy‐substituted phenylhydrazine deviated from the general electronic trend; side reactions under the reaction conditions could have suppressed productive conversion, such that **3n** was obtained only in minor amounts. In order to expand the compound collection and to introduce further heterocyclic NP‐fragments, we employed a method for the annulation of pyridines to cyclic ketones by means of Au(III)‐catalyzed cyclizations [44, 45, 46]. To this end, **C** was treated with propargylamines **6** in the presence of KAuCl_4_ at 130°C under microwave irradiation (Scheme 3). Thereby, PNPs with edge‐fusion of a pyridine ring and an AOHI‐fragment were formed in 55–62% yield. For the synthesis of further edge‐fuzed PNPs, on the one hand, a Friedländer synthesis was employed, yielding quinoline‐AOHI PNP **9**, and on the other hand, ketone **C** was subjected to Pictet–Spengler synthesis, which delivered isomeric spiro‐tetrahydrocarbolines **11** (Scheme 3). In total, a collection of 27 mesembrine‐inspired PNPs was synthesized in which the AOHI fragment is fuzed to different NP‐derived heterocycles.

### 2.2 Cheminformatics Analysis of the PNP Collection

**FIGURE 2:**
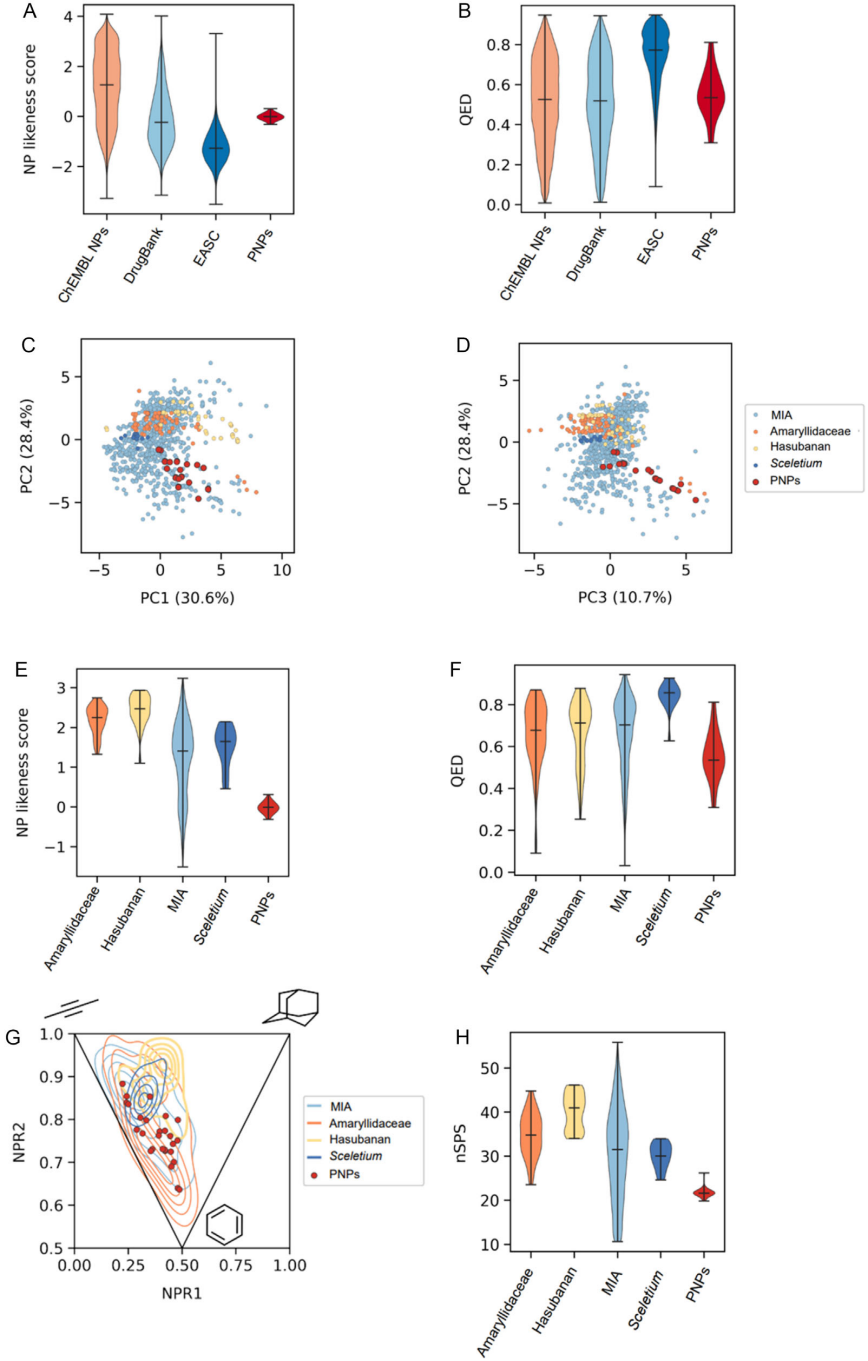
Chemoinformatics‐based characterization of PNPs. (A,B) NP‐likeness scores and quantitative estimate of druglikeness (QED) values position PNPs between NPs and drug‐like compounds. (C,D) Principal component analysis (PCA) of 17 molecular descriptors of PNPs and alkaloid classes. Explained variance is shown in parentheses. (E) Natural product‐likeness scores of PNPs show the closest resemblance to MIAs. (F) QED values of PNPs and alkaloid classes indicate a balanced NP/drug‐like profile. (G) Shape analysis using principal moments of inertia (PMI) of PNPs and alkaloid classes. NPR1 and NPR2 refer to the first and second normalized PMIs. (H) Normalized spatial score (nSPS) of PNPs and alkaloid classes.

The PNP compound collection was characterized in terms of molecular properties using the RDKit [47] and compared to *Sceletium*, Amaryllidaceae, Hasubanan, and MIAs listed in the ChEMBL [48] and COCONUT [49] databases, experimental and approved drugs from DrugBank [50, 51], and the Enamine Advanced Screening Collection (EASC) [52]. Principal component analysis (PCA) and unifold manifold approximation and projection (UMAP) of 17 molecular descriptors, utilizing data from DrugBank and ChEMBL NPs and PNPs [53], are detailed in Figure S14 of the Supporting Information. NP‐likeness [54] scores and quantitative estimate of druglikeness (QED) [55] values were analyzed to quantify fragment contributions. PNPs bridge the distribution of DrugBank and ChEMBL NPs, indicating their role at the NP/drug interface. (Figure 2A,B). For the assessment of the key physicochemical properties like lipophilicity (LogP and pLogD at pH 7.4), topological polar surface area (tPSA), and hydrogen‐bond donor/acceptor counts were calculated using SwissADME [56] and MolGpka [57] (Table S2). The physicochemical parameters of PNPs indicate moderate lipophilicity and polar surface areas suitable for oral drugs, while also showing enhanced polarity and hydrogen‐bonding capacity. Comparison of the PNPs with alkaloid subsets through PCA revealed that the PNPs define a distinct region in the alkaloid space and are somewhat separated from most of the alkaloid families but remain within the MIA domain (Figure 2C,D). While their NP likeness is less pronounced compared to the other alkaloid families (Figure 2E), QED values reveal that PNPs together with MIAs, Hasubanan‐ and Amaryllidaceae‐alkaloids occupy drug‐like space (Figure 2F). General Shape analysis by PMI plots [54] positioned the PNPs among DrugBank and ChEMBL compounds (Figure S15; see the Supporting Information). When compared to alkaloids, the PNPs mainly resided in nonplanar shape space, which is also typical for MIAs. While the PNPs also populate Amaryllidaceae alkaloid chemical space, their crossover population with *Sceletium*‐ and Hasubanan alkaloids is smaller (Figure 2G). Scaffold complexity with relevance to bioactivity was analyzed by means of the spatial score (SPS), which integrates hybridization, content in stereogenic carbons, nonaromatic rings, and branching into a single descriptor that directly correlates with bioactivity [58, 59]. Normalization to the number of heavy atom neighbors yields the normalized SPS (nSPS), which describes spatial complexity density. The analysis revealed that the PNPs scored lower than natural alkaloids, but they remained within the nSPS range of 20–40, favorable for potency and target selectivity and typical for biologically privileged compound classes (Figure 2H).

### 2.3 Biological Assessment of the PNP Collection

Since PNPs are unprecedented and not linked to established bioactivities, we employed unbiased phenotypic profiling by means of CPA to broadly monitor bioactivity [60]. This assay utilizes multichannel fluorescent imaging to capture changes across cellular compartments. Changes are translated into 579 quantitative features [61]. which are z‐Scores that indicate alterations to the DMSO control and offer a complete view of compound‐induced phenotypic response. Induction values are then defined as the percentage of significantly altered features relative to DMSO controls. They serve as a general measure of bioactivity, and compounds are considered active when the Induction is ≥5%. For comparison of biosimilarity, phenotypic profiles are compared and considered similar when BioSim ≥75%. Compounds with biosimilar CPA profiles may share similar targets or modes of action (MOA) [62]. For more conclusive target hypothesis generation beyond mere profile comparison in CPA, we introduced subprofile analysis [59]. In this approach, features are extracted from the full biosimilar profiles, that upon treatment with compounds, are changed in the same direction. A cluster subprofile is generated from such features to evaluate cluster biosimilarity. Initial assessment of mesembrine itself revealed that the alkaloid did not induce any phenotypic changes in this particular phenotypic assay. This finding raised the question whether a combination of the AOHI fragment, characteristic of this alkaloid, with other NP fragments might induce new bioactivity.

**FIGURE 3:**
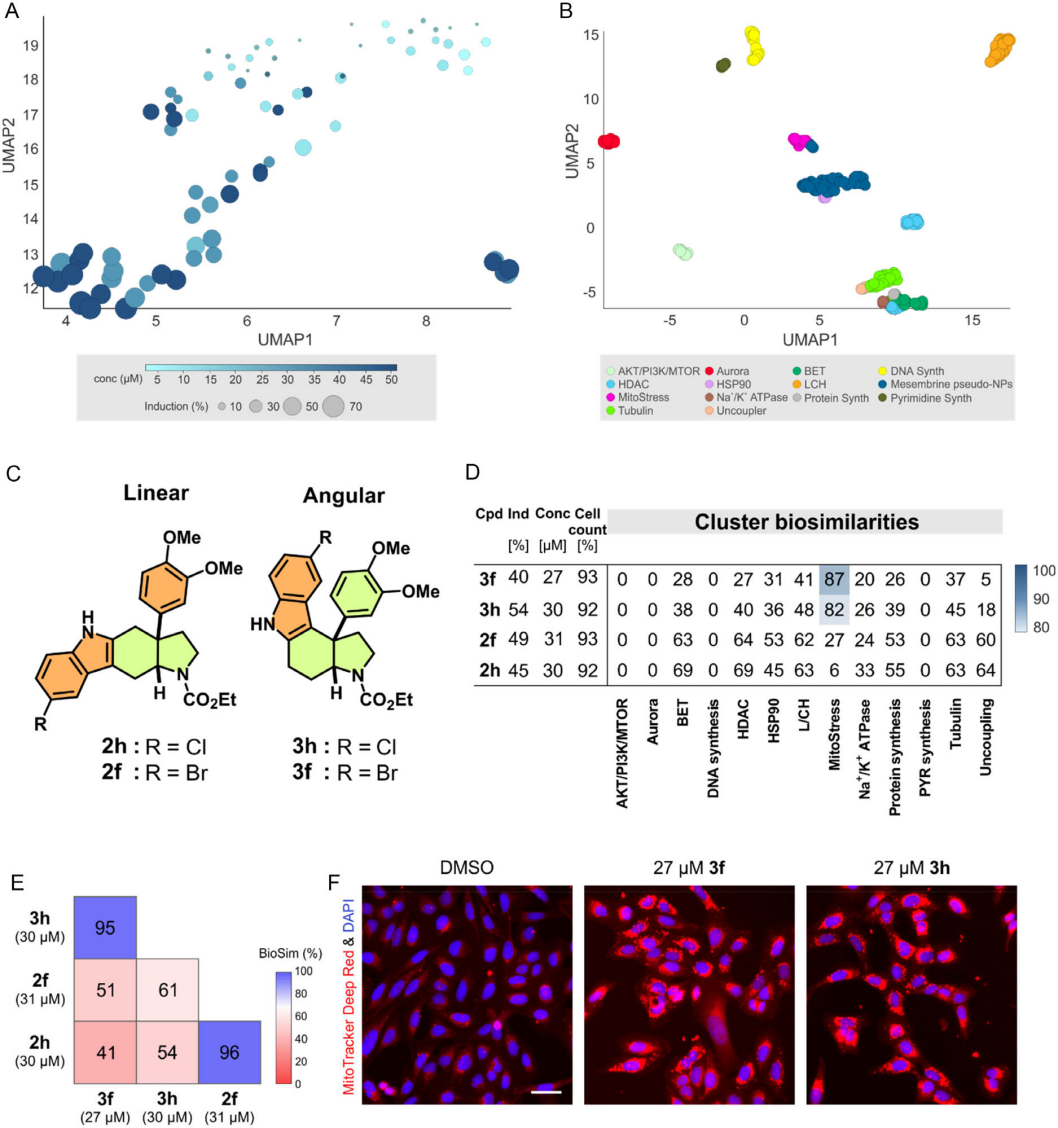
Analysis of the PNPs for bioactivity in the cell painting assay. (A) UMAP plot using the profiles for all active PNPs at different concentrations. The color coding corresponds to the concentration at which the profile was recorded. The size of the symbols depicts the induction value. Not normalized, 10 neighbors. (B) Mapping the bioactivity of the PNPs collection in the space defined by the 13 clusters. Not normalized, 10 neighbors. (C) Structures of the regioisomers **2f**, **2h**, **3f**, and **3h**. (D) Cluster biosimilarity heatmap for compound **2f**, **2h**, **3f**, and **3h**. Biosimilarities are given as percentage values. L/CH: lysosomotropism/cholesterol homeostasis; PYR: pyrimidine. (E) Profile biosimilarity cross‐correlation for **2f**, **2h**, **3f**, and **3h**. (F) MitoTracker Deep Red and nuclear staining (DAPI) for **3f** and **3h**. Images from CPA are shown. Scale bar: 50 µm.

CPA analysis was employed to determine whether (i) the library members in general exhibit activity, (ii) established PNP design principles translate into phenotypic diversity, and (iii) the resulting bioactivity profiles might inform target‐ or MoA hypotheses. Compounds were investigated in the CPA at concentrations of 2–50 µM. *Spiro*‐fuzed tryptamine **11** and unsubstituted pyridine derivative **7a** were inactive in CPA at the tested concentrations. However, attachment of a methyl group or a phenyl ring to the pyridine ring (**7b** and **7c**) yielded induction values of 36.8% and 60.8% at 30 μM. Analysis of the PNP profiles using dimensionality reduction revealed that induction values increase with increasing concentrations (Figure 3A). Interestingly, compounds **3f**, **3h**, and **3g** form a distinct cluster in a dimensionality reduction analysis at concentrations of 10 and 30 µM.

The different subscaffolds did not form separate clusters besides the halogenated congeners **3f**, **3h**, and **3g**, which all represent angular indoles (Figure S16, see Supporting Information). However, not all angular indoles cluster together, indicating that modifications around the scaffold have a profound effect on the type of bioactivity. The profiles of the PNP collection were mapped in the CPA space defined by the 13 bioactivity clusters (Table S3) [63]. The compounds formed two distinct clusters. PNPs **3f**, **3h**, and **3g** were located next to the MitoStress cluster (Figure 3B), which is related to mitochondrial fragmentation and induction of integrated stress response [64]. The corresponding regioisomers. **2f** and **2h** induced different phenotypes: while the profiles of **3f** and **3h** showed high similarity to the MitoStress cluster, **2f** and **2h** did not (Figure 3C,D).

The profiles of the corresponding regiosiomers were not biosimilar (Figure 3E), and mitochondrial fragmentation was observed for **3f** and **3h** but not for **2f** and **2h** (Figures 3F and S17). In general, regioisomers generated distinct morphological changes while being structurally diverse (high intrascaffold similarity of 0.88 and lowered with 0.66–0.75 across scaffolds **2** and **3** (Figure S18).

**FIGURE 4:**
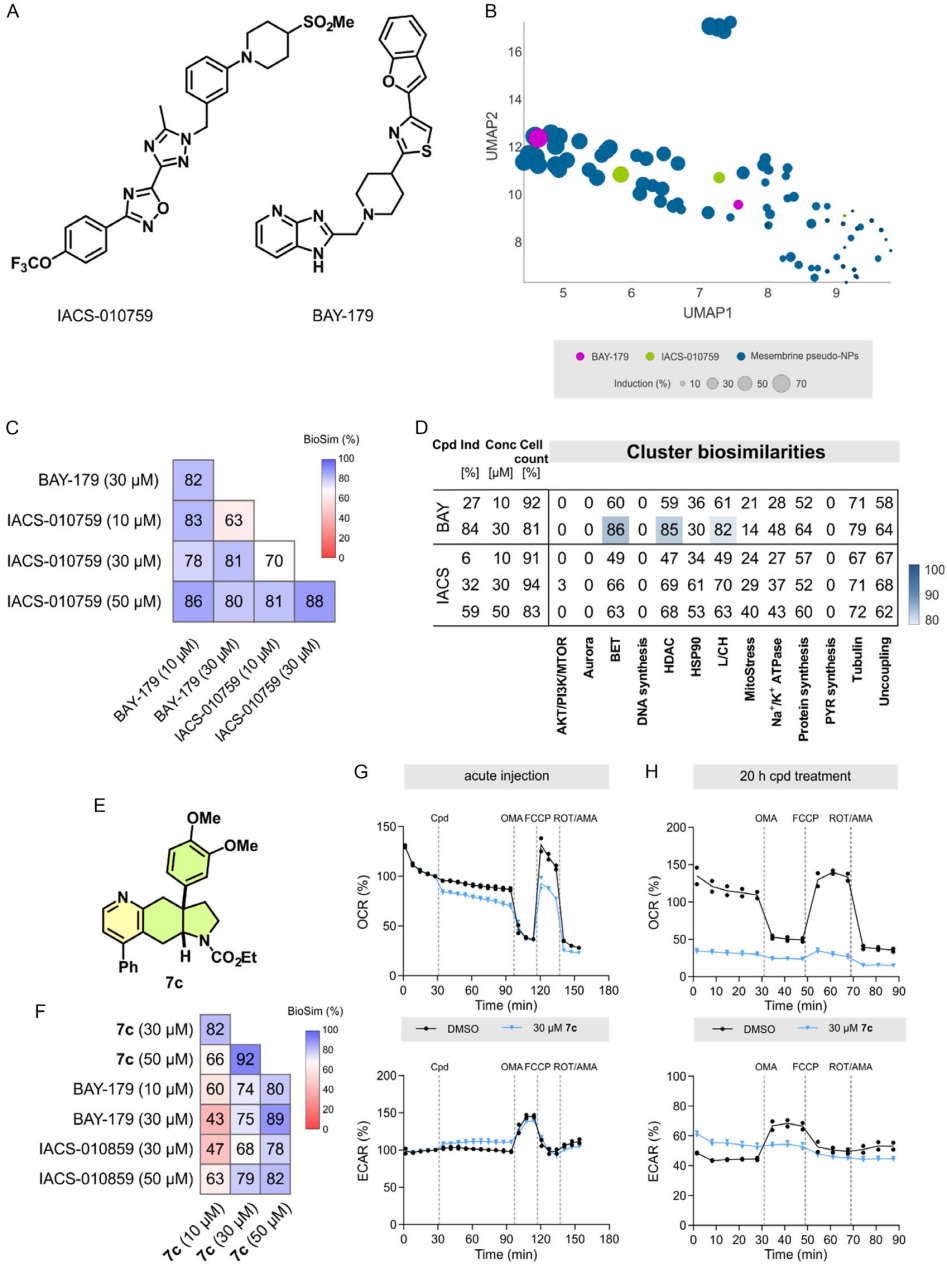
CPA detects inhibition of complex I activity. (A) Chemical structures of the complex I inhibitors BAY‐179 and IACS‐010759. (B) UMAP plot using the profiles of the PNP collection and BAY‐179 and IACS‐010759. The size of the symbols depicts the induction value. Not normalized, 10 neighbors. (C) Profile biosimilarity cross‐correlation for BAY‐179 and IACS‐010759. (D) Cluster biosimilarity heatmap for compound BAY‐179 and IACS‐010759. Biosimilarities are given as percentage values. L/CH: lysosomotropism/cholesterol homeostasis; PYR: pyrimidine. (E) Chemical structure of compound **7c**. (F) Biosimilarity for the profiles of **7c** as compared to the profiles recorded for BAY‐179 and IACS‐010759. (G, H) Influence on mitochondrial respiration of **7c** as determined using the Seahorse MitoStress Test. U‐2OS cells were either directly exposed to the compound (acute injection, G) or treated with the compound for 20 h (H) prior to measuring the oxygen consumption rate and the extracellular acidification rate (ECAR) after acute injection. Data (*N* = 2) are representative of three independent experiments (*n* = 3). Cpd: compound; OMA: oligomycin A; ROT: rotenone; and AMA: antimycin A.

The second cluster was located in the UMAP plot next to the cluster of HSP90 inhibitors (Figure 3B). However, both clusters can clearly be distinguished when analyzed separately (Figure S19). No similarity to a common cluster was detected (Table S3), suggesting a MoA that differs from the bioactivity of the 13 defined clusters. Among the reference compounds with biosimilar profiles were two inhibitors of the mitochondrial complex I, i.e., BAY‐179 and IACS‐010759 (Figure 4A,B). The profiles of these tool compounds are biosimilar (Figure 4C) and at 10 µM lack similarity to the 13 defined clusters (Figure 4D). At 30 µM, the profile of BAY‐179 shows similarity to the BET, HDAC, and the lysosomotropism/cholesterol homeostasis cluster (Figure 4D). However, the activity of 30 µM BAY‐179 was clearly distinguishable from the MoA of this cluster (Figure S20). Therefore, the detected activity in CPA may be related to the inhibition of complex I.

A target or MoA hypothesis generation based on CPA profiles is solidified if compounds annotated for a given target are structurally dissimilar but show profile similarity. Indeed, BAY‐179 and IACS‐010759 do not share a common scaffold and, therefore, the observed profile similarity most likely stems from modulation of complex I. Impairment of the mitochondrial electron transport chain (ETC) by modulating the different complexes does not lead to a common CPA profile.

Inhibition of complex III suppresses pyrimidine biosynthesis, and these inhibitors cluster together with modulators of enzymes involved in pyrimidine biosynthesis (Figure S21A), such as dihydroorotate dehydrogenase (DHODH) and UMP synthase (UMPS) [65]. Oligomycin A, which inhibits complex V of the ETC, induces mitochondrial fragmentation and integrated stress response, which is detectable as a distinct phenotype (Figure S21A) [64]. Moreover, uncouplers of the mitochondrial proton gradient, like FCCP, form as well as a separate CPA cluster (Figure S21A) [63]. The profiles of complex I inhibitors differ from those recorded for modulators of complex III, complex V, or uncoupling agents, i.e., the corresponding clusters (Figure 4D) and (Figure S21B). Therefore, CPA can precisely detect the level at which the ETC is impaired.

To explore whether the PNPs from the large cluster impair mitochondrial respiration, metabolic flux was analyzed after treatment of U‐2OS cells with compound **7c** (Figure 4E) using the Seahorse technology. The profiles of this small molecule display biosimilarity to the profiles of BAY‐179 and IACS‐010759 at the higher concentrations (Figure 4F). After acute injection, **7c** provoked a moderate decrease in the oxygen consumption rate (OCR), which is a measure of mitochondrial respiration. At the same time, the glycolysis rate slightly increased as a compensatory mechanism, which was determined using the extracellular acidification rate (ECAR) (Figure 4G). After an incubation for 20 h, **7c** almost completely abolished mitochondrial respiration (Figure 4H). Similar to BAY‐179 and IACS‐010759, the profiles recorded for **7c** were not biosimilar to the pyrimidine biosynthesis cluster (which includes complex III modulators), the MitoStress cluster (which include the complex V inhibitor oligomycin A) or to uncoupling agents (Figure S22). Moreover, the complex II inhibitors carboxin and lonidamine were not active in Cell painting at 10 and 30 µM [64, 66]. Carboxin was shown to interfere with respiration only after inhibition of complex I, whereas it does not impair basal respiration [66]. As compound **7c** inhibits basal respiration and its profile does not show any biosimilarity to other ETC inhibitors or uncoupling agents, its activity and the PNPs in this large cluster most likely relates to impairment of the ETC at the level of complex I.

## 3 Conclusion

We describe the design and synthesis of a PNP collection through the fusion of the AOHI NP fragment with heterocycles broadly distributed across major alkaloid families. We developed a scalable, microwave‐assisted variant of the intramolecular Diels–Alder furan reaction, building on the approach introduced by Padwa et al. Cheminformatics‐driven analyses revealed that the PNPs occupy an exclusive region among alkaloids and between drug‐like scaffolds and NPs. Extensive phenotypic profiling via CPA highlighted that the fusion topology of indoles directly translates into distinct phenotypic fingerprints, with the emphasis on mitochondrial alteration, consistent with complex I perturbation. Seahorse flux analysis demonstrated that fusion with a pyridine revealed consistent and robust modulation of mitochondrial function, suggesting an upstream effect of complex V. Taken together, these findings highlight that the AOHI scaffold is a very valuable alkaloid fragment for PNP design and identify mitochondria as a major source of the biological mode of action of this compound class.

## Supporting Information

Additional supporting information can be found online in the Supporting Information Section. **Supporting** **Scheme S1:** Synthetic route to ketone C. **Supporting** **Fig. S1** **:** Overview of the synthesized pseudo‐NPs. **Supporting** **Fig. S2** **:** Structure elucidation of Linear Indole 2 using COSY NMR. **Supporting** **Fig. S3** **:** Structure elucidation of Linear Indole 2 using COSY NMR (Zoom). **Supporting** **Fig. S4** **:** Structure elucidation of Linear Indole 2 using NOESY NMR. **Supporting** **Fig. S5** **:** Structure elucidation of Linear Indole 2 using NOESY NMR (zoom). **Supporting** **Fig. S6** **:** Structure elucidation of fused pyridine 7 using COSY NMR. **Supporting** **Fig. S7** **:** Structure elucidation of fused pyridine 7 using COSY NMR (zoom). **Supporting** **Fig. S8** **:** Structure elucidation of fused pyridine 7 using NOESY NMR. **Supporting** **Fig. S9:** Structure elucidation of fused pyridine 7 using NOESY NMR (zoom). **Supporting** **Fig. S10:** Structure elucidation of angular indole 3 using COSY NMR. **Supporting** **Fig. S11** **:** Structure elucidation of angular indole 3 using COSY NMR (zoom). **Supporting** **Fig.** **S12:** Structure elucidation of angular indole 3 using NOESY NMR. **Supporting** **Fig. S13** **:** Structure elucidation of angular indole 3 using NOESY NMR (zoom). **Supporting** **Fig. S14** **:** A) Principal Component Analysis (PCA) on 17 molecular descriptors. B) Uniform Manifold Approximation and Projection (UMAP) on 17 molecular descriptors. **Supporting** **Fig. S15** **:** A) normalized Spatial score. B) Böttcher‐Score. C) normalized Principal Moments of Inertia (PMIs). Comparisons are based on 78k natural products registered in ChEMBL, 11k compounds in the DrugBank, and a random sample of 50k compounds from the Enamine Advanced Screening Collection. **Supporting** **Fig. S16** **:** Profile analysis of the PNP collection based on the substructures. UMAP plot using the profiles for all active PNPs at different concentrations. The size of the symbols depicts the induction value. Not normalized, 10 neighbors. **Supporting** **Fig. S17** **:** MitoTracker Deep Red and nuclear staining (DAPI) for 2f and 2h. Images from CPA are shown. Scale bar: 50 µm. **Supporting** **Fig. S18** **:** Chemical similarity was determined using the Tanimoto score of the Morgan fingerprints with radius 2, as implemented in the RDKit. (A) Linear and angular indole fused AOHIs. (B) Chemical similarity is shown as AP Tanimoto similarities. **Supporting** **Fig. S19** **:** Profile analysis of the PNP collection and HSP90 inhibitors. UMAP plot using the profiles for all active PNPs at different concentrations and the profiles of HSP90 inhibitors. The size of the symbols depicts the induction value. Not normalized, 10 neighbors. **Supporting** **Fig. S20** **:** Analysis of the profiles of BAY‐179 and IACS‐010759. UMAP plot using the profiles of BAY‐179 and IACS‐010759 at different concentrations and along with selected clusters. Not normalized, 10 neighbors. L/CH: lysosomotropism/cholesterol homeostasis. **Supporting** **Fig. S21** **:** Profile analysis of ETC inhibitors. (A) Cluster biosimilarity heatmap for compound for selected ETC inhibitors. Biosimilarities are given as percentage values. L/CH: lysosomotropism/cholesterol homeostasis; PYR: pyrimidine. (B) Profile biosimilarity cross‐correlation for ETC inhibitors. **Supporting** **Fig. S22** **:** Profile analysis for compound 7c. (A) Cluster biosimilarity heatmap for compound 7c. Biosimilarities are given as percentage values. L/CH: lysosomotropism/cholesterol homeostasis; PYR: pyrimidine. (B) Profile similarity for compound 7c and ETC inhibitors. **Supporting** **Table S1** **:** Optimization of the microwave‐assisted IMDAF reaction.

## Funding

This Study was supported by Max‐Planck‐Gesellschaft, European Union (DDHD), Innovative Medicines Initiative, European Union's Seventh Framework Programme, EFPIA companies, Netzwerke 2021

## Conflicts of Interest

The authors declare no conflicts of interest.

## Supporting information

Supplementary Material

## Data Availability

The data that support the findings of this study are available from the corresponding author upon reasonable request. The code used for data analysis in this study is publicly available at https://github.com/mpimp-comas/2025_Greiner_PseudoNPs.
