# Supplementary material for: Design, Synthesis, and Biological Evaluation of Pseudo‐Natural Products Inspired by Aryloctahydroindole Alkaloids
Source: ChemMedChem. 2026 Feb 12;21(3):e202501102. doi: 10.1002/cmdc.202501102 (PMC12900267; doi:10.1002/cmdc.202501102)

Design, Synthesis and Biological Evaluation of Pseudo-Natural Products Inspired by Aryloctahydroindole Alkaloids

Luca C. Greiner,[a] Freddy A. Bernal,[a,b] Sasikala Thavam,[a] Maite Brachthäuser,[a] Sonja Sievers,[a,b] Slava Ziegler,[a] Herbert Waldmann*[a,c]

---

[a] Luca C. Greiner, Freddy A. Bernal,
S. Thavam, M. Brachthäuser, S. Sievers,
Slava Ziegler, Herbert Waldmann,
Abteilung Chemische Biologie
Max-Planck-Institute für Molekulare Physiologie,
Otto-Hahn-Straße 11, 44227 Dortmund, Germany

[b] Freddy A. Bernal, Sonja Sievers,
Compound Management and Screening Center,
Otto-Hahn-Straße 15, 44227, Dortmund, Germany

[c] Herbert Waldmann,
Fakultät Chemie und Chemische Biologie
Technische Universität Dortmund
Otto-Hahn-Straße 6, 44221 Dortmund, Germany

Table of Contents

Supporting Information Figures and Schemes 3

All Pseudo-Natural Product Identifications 4

General Synthetic Experimental Details 5

Procedure for the Suzuki Coupling to S3 6

Procedure for the Curtius Rearrangement to S6 7

Procedure for the Synthesis of the Cyclization Precursor (A) 8

Procedures for the Synthesis of Hexahydroindole (B) 10

Procedure for the Synthesis of Octahydroindole (C) 11

General Procedure for the Fischer Indole Synthesis for Classes 2 and 3 12

General Procedure for Gold-Catalyzed Pyridine Synthesis for Class 7 12

Structure Determination by NMR Analysis 35

General Chemoinformatic Data 47

Biological Experimental Details 51

General Biological Experimental Details 51

Cell Painting Assay Details 52

Seahorse XF Mito Stress Test 56

References 65

NMR Spectra 68

Supporting Information Figures and Schemes

Synthetic Scheme of the synthesis of AOHI via an intramolecular Diels-Alder route.

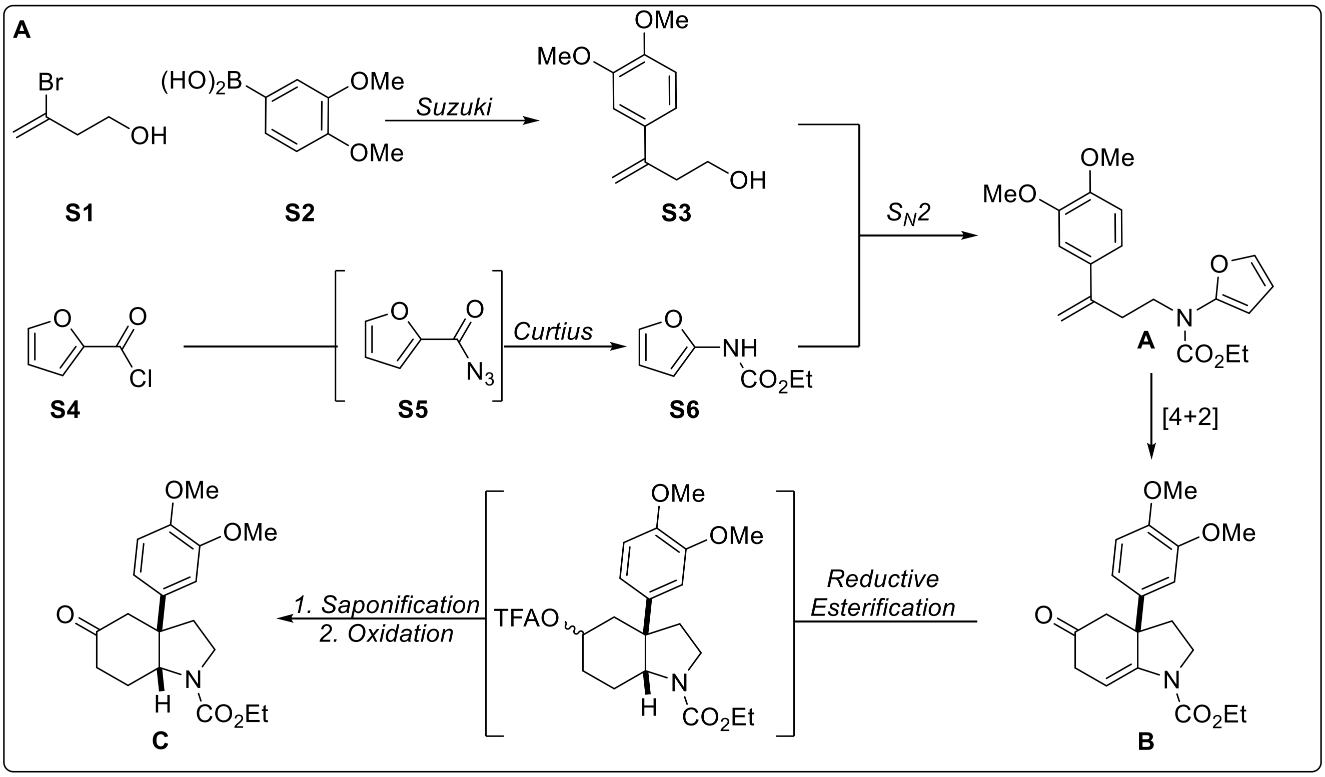

Scheme S1. Synthetic route to ketone **C**

All Pseudo-Natural Product Identifications

Compounds included in the Pseudo-Natural Product (PNP) collection with their corresponding yields from the final synthetic step. **2**: 12 members; **3**: 7 members; **5**: 1 member; **7**: 3 members; **9**: 1 member; **11**: 1 member

Linear Fused Indoles (**2**)

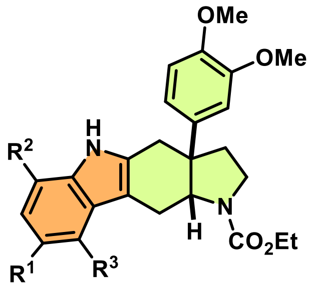

Entry	R^1	R^2	R^3	Yield [%]
**2a**	H	H	H	68
**2b**	OCF_3	H	H	65
**2c**	OBn	H	H	44
**2d**	H	OMe	H	40
**2e**	SO_2NH_2	H	H	65
**2f**	Br	H	H	60
**2g**	H	Br	H	42
**2h**	Cl	H	H	62
**2i**	Cl	Cl	H	51
**2j**	H	Cl	Cl	40
**2k**	F	H	H	32
**2l**	CO_2H	H	H	55

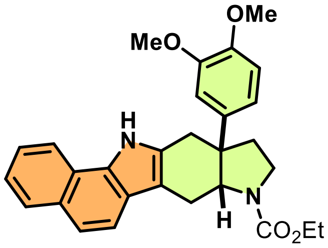

2m : 40%

Angular Fused Indoles (**3**)

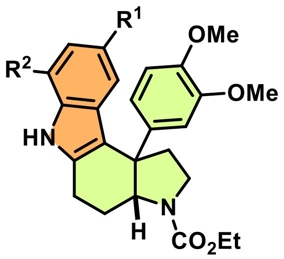

Entry	R^1	R^2	Yield [%]
**3a**	H	H	25
**3b**	OBn	H	18
**3c**	OMe	H	24
**3d**	Br	H	22
**3e**	H	Br	30
**3f**	Cl	H	18
**3g**	Cl	Cl	15

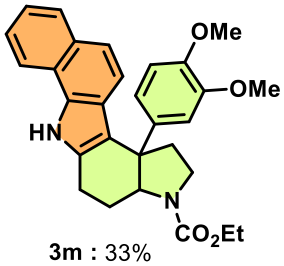

3m : 33%

Linear Fused Azaindole (**5**)

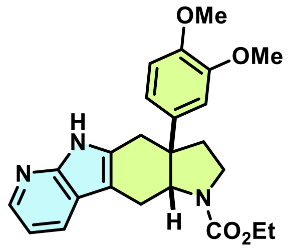

5 : 35 %

Linear Fused Pyridines (**7**)

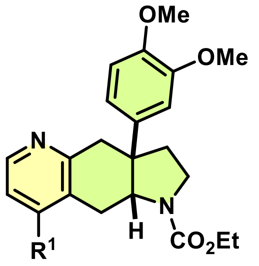

Entry	R^1	R^2	Yield [%]
**7a**	H	H	60
**7b**	OBn	H	62
**7c**	Br	H	55

Linear Fused Quinoline (**9**)

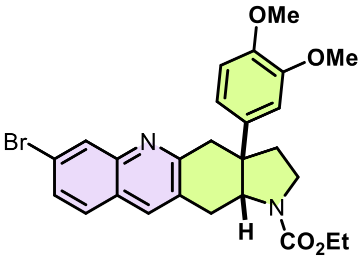

9 : 54%

Spiro Fused Tetrahydrocarboline (**11**)

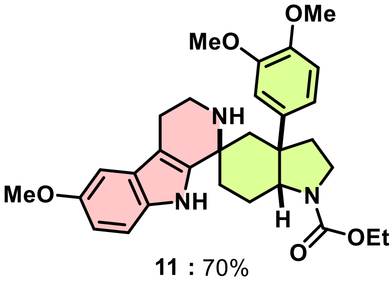

11 : 70%

Figures S1. Overview of the synthesized pseudo-NPs.

General Synthetic Experimental Details

Unless otherwise noted, all commercially available compounds were used as provided without further purification. Solvents used for chromatography were technical grade. Analytical thin-layer chromatography (TLC) was performed using a gradient of petroleum ether and ethyl acetate, based on Merck aluminum TLC sheets (silica gel $60F_{254}$). Compounds were visualized by irradiation with UV light. Column chromatography was performed using silica gel Merck 60 (particle size 0.040-0.063 mm). ^{1}H-NMR and ^{13}C-NMR were recorded on a Bruker DRX400 (400 MHz), Bruker DRX500 (500 MHz), INOVA500 (500 MHz), or Bruker DRX700 using CD_2Cl_2, $CDCl_3$, or CD_3COCD_3 as solvent. Data are reported in the following order: chemical shift (δ) values are reported in ppm with the solvent resonance as internal standard (CD_2Cl_2: $\delta = 5.32$ ppm for ^{1}H, $\delta = 53.84$ ppm for ^{13}C; $CDCl_3$: $\delta = 7.26$ ppm for ^{1}H, $\delta = 77.16$ ppm for ^{13}C); multiplicities are indicated s (singlet), d (doublet), t (triplet), q (quartet), m (multiplet); coupling constants (J) are given in Hertz (Hz). High-resolution mass spectra were recorded on a LTQ Orbitrap mass spectrometer coupled to an Accela HPLC-System (HPLC column: Hypersyl GOLD, 50 mm x 1 mm, particle size 1.9 µm, ionization method: electron spray ionization). Data collection for single crystal X-ray structure analyses was conducted on a Bruker D8 Venture four-circle diffractometer by Bruker AXS GmbH using a PHOTON II CPAD detector by Bruker AXS GmbH. X-ray radiation was generated by microfocus sources IµS 3.0 Mo by Incoatec GmbH with HELIOS mirror optics and a single-hole collimator by Bruker AXS GmbH. For the data collection, the programs APEX 3 Suite (v.2018.72) with the integrated programs SAINT (integration) and SADABS (adsorption correction) by Bruker AXS GmbH were used. Microwave reactions were carried out in a CEM Discover SP Activent machine.

Procedure for the Suzuki Coupling to S3

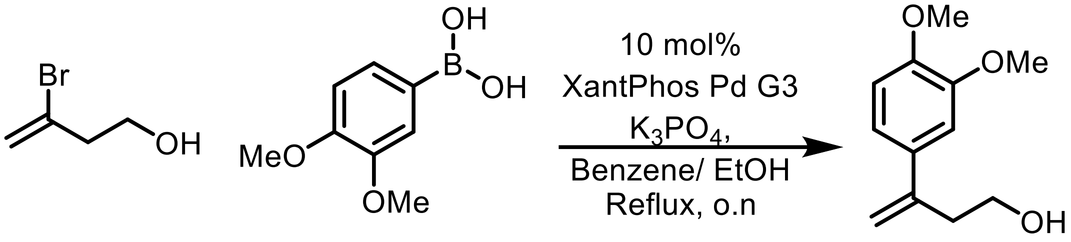

The procedure for the preparation of homoallylic alcohol by the Suzuki-coupling was performed according to the literature.[1-3] To a stirred mixture of XantPhos Pd G3 4 (3.4 g, 3.64 mmol), K_3PO_4 (2 M aqueous solution, 50 mL), benzene (150 mL), and 3-bromo-3-buten-1-ol **S1** (5.5 g, 36.4 mmol) was added dropwise a clear solution of aryl 1.5 equiv. of boronic acid **S2** (9.9 g, 54.6 mmol) in EtOH (20 mL) at 25°C. The mixture was then heated to reflux under an Ar atmosphere, with the oil bath reaching approximately 80°C overnight. When the reaction was complete, as monitored by TLC, the mixture was cooled to 25 °C, and 25 mL of 30 % aqueous H_2O_2 (caution) was added dropwise to destroy residual boron species. The mixture was stirred at 25°C for 1 h, diluted with diethyl ether (50 mL), and extracted with ether (40 mL x 3). The combined organic phases were washed with brine (50 mL), dried over Na_2SO_4, concentrated *in vacuo*, and the resulting oil was purified by column chromatography (PE/EtOAc = 5/1) to afford the desired compound **S3** (5.8 g, 76%).

The spectroscopic Data were in alignment with the reported literature.[1]

Note: *The use of XantPhos Pd G3 precatalys (high stability towards ambient conditions and straightforward handling) with mild base lile aqueous K_3PO_4, instead of $Pd(PPh_3)_4$ or PD-PEPPSI and K_2CO_3, resulted in a more robust (significantly reduced homocoupling in bigger*

---

[1] M. T. Reetz, H. Guo, J. A. Ma, R. Goddard, R. J. Mynott, *J. Am. Chem. Soc.* 2009, *131*, 4136-4142. [2] A. Padwa, M. A. Brodney, M. Dimitroff, B. Liu, T. Wu, *J. Org. Chem.* **2001**,*66*, 3119−3128. [3] Q. Huang, A. Fazio, G. Dai, M. A. Campo, R. C. Larock, *J. Am. Chem. Soc.* **2004**, *126*, 7460−7461

scales) and scalable transformation with higher yields compared to reported work. This improvement is due to the milder generation and stability of the active Pd(0) species, allowing for a scale-up to 5.5 g.

Procedure for the Curtius Rearrangement to S6

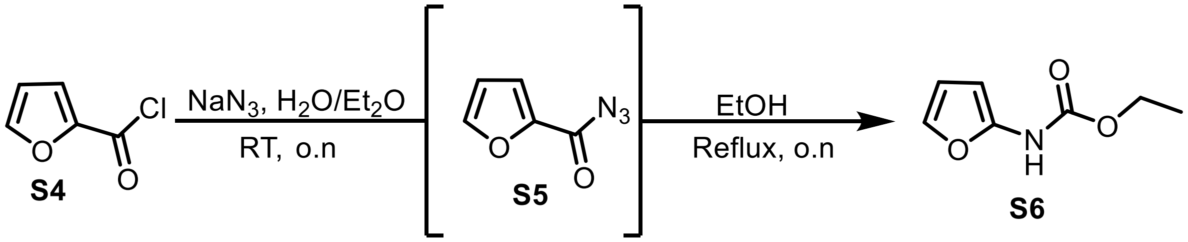

50 mL of an aqueous solution containing 1.2 equiv. NaN_3 (10.0 g, 153 mmol) was added dropwise to a solution of to 1.0 equiv. 2-Furoylchloride **S4** (16.7 g, 128 mmol) in 200 mL of diethyl ether (250 mL) at a temperature of 0 °C. The mixture was vigorously stirred for 15 minutes and allowed to gradually increase to room temperature, and the mixture was stirred overnight to allow the reaction to proceed to completion. After separation, the organic phase was washed with Brine (20 mL x 3), dried with Na_2SO_4, and concentrated *in vacuo,* giving a crude white powder of 2-Furoylazide **S5**. The crude mixture was dissolved in EtOH (200 mL) and refluxed overnight in the presence of a protective shield (shock and heat sensitivity).[3,4] The solution was concentrated under reduced pressure, and the residue was purified by silica gel chromatography to give the furan-2-ylcarbamic acid ethyl ester **S6** (15.9 g, 80%) as a yellow oil.[2]

The spectroscopic data aligned with the literature.[3]

---

[2] A. Padwa, M. A. Brodney, K. Satake, C. S. Straub, *J. Org. Chem.* **1999**, *64*, 4617
4626. [4] A. Padwa, K. R. Crawford, P. Rashatasakhon, M. Rose, *J. Org. Chem.* **2003**, *68*, 2609-2617.

Procedure for the Synthesis of the Cyclization Precursor (A)

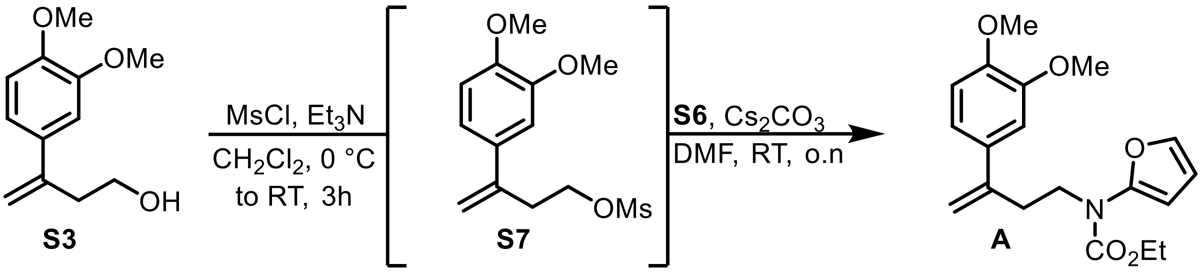

To a solution of 1.0 equiv. of **S3** (5.0 g, 27 mmol), Et_3N (5.0 mL) in dry CH_2Cl_2 (100 mL), MsCl (2.3 mL, 29.5 mmol) was added dropwise at 0 °C. After addition, the reaction was allowed to warm to room temperature and stirred for 3 h. After completion, as monitored by TLC, the mixture was diluted with water (10 mL), extracted with CH_2Cl_2 (50 mL x 3), washed with brine, dried over $MgSO_4$, and concentrated *in vacuo,* giving 7.0 g of crude mesylate **S7**, which was used directly without purification. Next, a stirred suspension of **S7** and 1.5 equiv. of Cs_2CO_3 (13 g, 40.5 mmol) in DMF (100 mL), received dropwise addition of 1.5 equiv. of **S6** (6.3 g, 40.5 mmol**)** in THF (5 mL) and was stirred overnight. After dilution with H_2O and diethyl ether extraction, the mixture was dried over $MgSO_4$ and concentrated *in vacuo*. purification using silica gel chromatography (PE : EtOAC = 5 : 1), yielded carbamate **A** (5.6 g, 60%) of as a yellow oil. Spectroscopic data aligned with the literature.[3]

[3] A. Padwa, M. A. Brodney, K. Satake, C. S. Straub, *J. Org. Chem.* **1999**, *64*, 4617

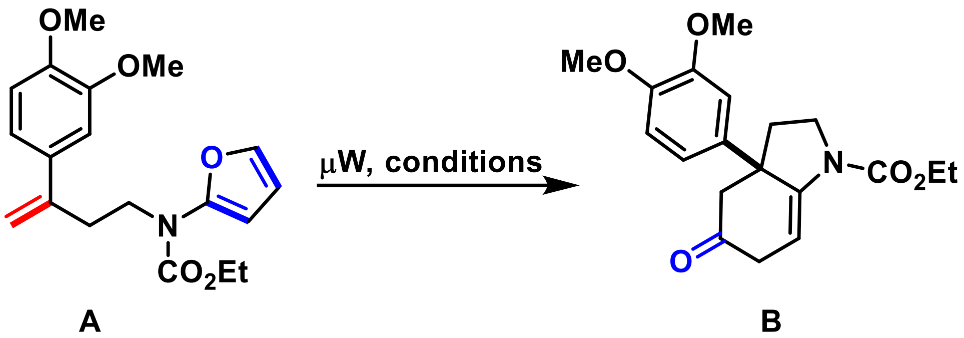

Table S1. Optimization of the microwave-assisted IMDAF reaction [a]

Entry	Solvent	c[mM]	T [°C]	T[h]	Yield[%][b]	ε_r	tan δ
1[c]	Toluene	200	180	2	5	2.38	0.04
2[c]	Toluene	200	220	2	3	2.38	0.04
3[c]	Toluene	200	220	3	3	2.38	0.04
4[c]	Toluene	200	220	5	8	2.38	0.04
5[c]	DMSO	200	200	2	-[d]	46.7	0.825
6[c]	DMF	200	200	2	-[d]	36.7	0.161
7[c]	MeCN	200	150	2	-[d]	37.5	0.062
8[c]	*o*-DCB	200	170	0.5	35	10.13	0.042
9[c]	*o*-DCB	200	180	2	52	10.13	0.042
**10**	***o*-DCB**	**20**	**200**	**2**	**58**	**10.13**	**0.042**
11	EtOH	20	150	2	-	24.3	0.941
12	THF	20	130	2	<1	7.58	0.047
13	CF_3Ph	20	150	2	8	9.18	0.061

[a] Relative permittivity (ε_r) and dielectric loss tangent (tan δ) values are given at 20 °C.

[b] Yields are isolated yields. [c] black tar formation. [d] complex mixture.

Procedures for the Synthesis of Hexahydroindole (B)

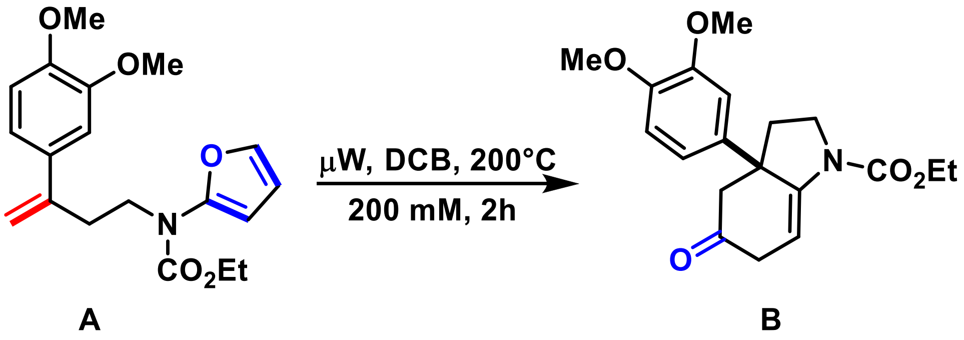

Microwave, 20 mM optimized variant: In a sealed 25 mL microwave pressure vessel equipped with a magnetic stirrer, **A** (86 mg, 0.25 mmol) was dissolved in 1,2-dichlorobenzene (12.5 mL) and was heated under microwave irradiation at 200 °C for 2h, resulting in a reddish solution. The high-boiling solvent was removed under high vacuum at 90°C using a Schlenk line equipped with a liquid nitrogen cold trap. The brown crude oil was purified using silica gel chromatography (PE : EtOAC = 3:1), yielding the hexahydroindole **B** (52 mg, 58%) as a yellow oil.

Spectroscopic data aligned with the literature.[3]

Microwave, scale-oriented variant: In a sealed 25 mL microwave pressure vessel equipped with a magnetic stirrer, **A** (2.2 g, 6.38 mmol) was dissolved in 1,2-dichlorobenzene (15 mL) and was heated under microwave irradiation at 200 °C for 2h, resulting in a reddish solution and insoluble black residues. The high-boiling solvent was removed under high vacuum at 90°C using a Schlenk line equipped with a liquid nitrogen cold trap. The brown crude oil was purified using silica gel chromatography (PE : EtOAC = 3:1), yielding the hexahydroindole **B** (1.1 g, 51%) as a yellow oil.

Spectroscopic data aligned with the literature.[3]

---

[3] A. Padwa, M. A. Brodney, K. Satake, C. S. Straub, *J. Org. Chem.* **1999**, *64*, 4617

Procedure for the Synthesis of Octahydroindole (C)

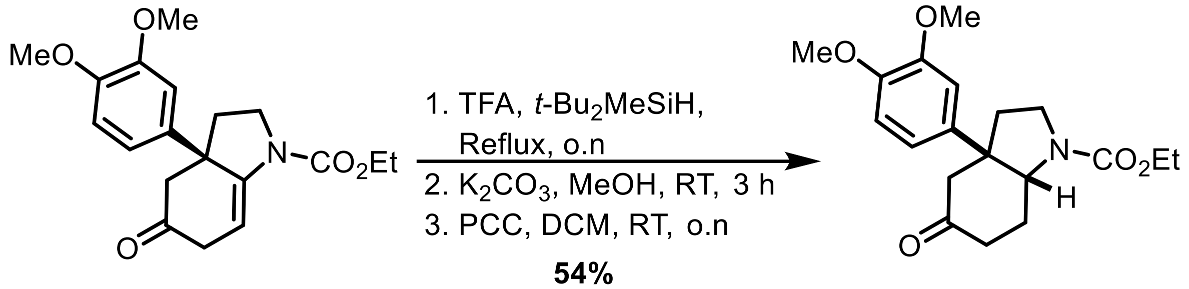

B **C**

A mixture of 1.0 equiv. keto-enecarbamate **B** (2.5 g, 7.24 mmol) (2.0 mmol) and (2.3 g, 14.5 mmol) of bis(*tert*-butyl)methylsilane in trifluoroacetic acid (22 mL) was heated at reflux for 3 h under an argon atmosphere. After allowing the mixture to cool to room temperature, it was concentrated *in vacuo*, and the residue was purified by short silica gel chromatography (PE : EtOAC = 3 : 1 to 1 : 1) to yield a mixture of diastereomers. The mixture was treated with K_2CO_3 in 50 mL of MeOH and stirred at room temperature for 1 hour. The mixture was concentrated under reduced pressure, diluted with water (20 mL), and extracted with ether (20 mL x 3). The combined organic layers were dried over $MgSO_4$ and concentrated *in vacuo*, and the resulting alcohol was used in the next step without further purification. A crude mixture containing the alcohol was subjected to PCC (3.1 g, 14.5 mmol), and 2.5 g of silica gel in 50 mL of CH_2Cl_2, and was stirred at room temperature overnight. Removal of the solvent under reduced pressure followed by flash silica gel chromatography gave (1.4 g, 54%) of the title compound as a colorless oil with trans isomer < 1% according to the NMR spectra:

^{1}H NMR (700 MHz, CDCl3) δ: 6.79 (m, 3H), 4.36 (s, 1H), 4.11 (m, 2H), 3.83 (m, 6H), 3.45 (m, 2H), 2.70 (m, 2H), 2.39 – 2.05 (m, 5H), 2.01 (m 1H), 1.23 (s, 3H).**^{13}C NMR (176 MHz, $CDCl_3$) δ:** 210.7, 155.2, 149.1, 147.9, 137.6, 117.7, 111.2, 109.1, 61.2, 61.0, 56.0, 55.9, 50.9, 49.2, 44.8, 37.8, 36.0, 26.4, 14.8. **HRMS (ESI)** calcd. for $C_{19}H_{25}N_1O_4$ [M + H]+ 348,1805 found 348,1807.

Note:[3] According to Padwa et al., the *cis-isomer shows a multiplet between δ 6.7−6.9 ppm for the three aromatic protons. In contrast, the NMR spectrum of the trans-substituted isomer generally shows three distinct signals at δ 6.8, 7.3, and 7.5*.[3]

General Procedure for the Fischer Indole Synthesis for Classes 2 and 3

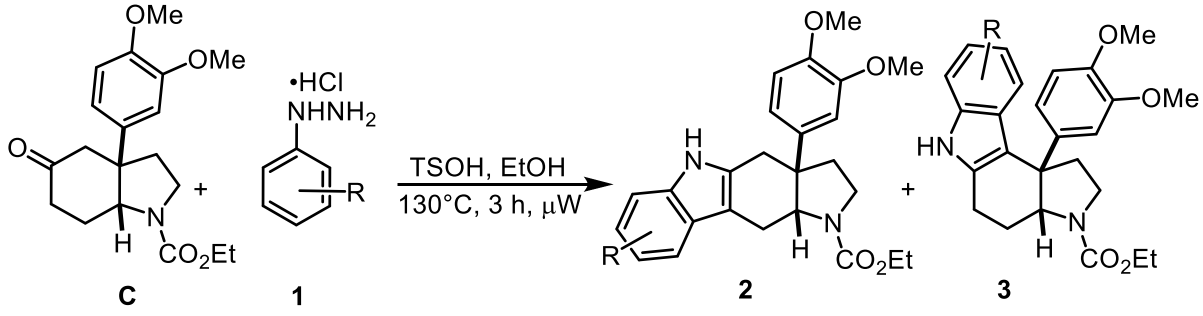

To a solution of ketone **C** (25 mg, 0.07 mmol, 1 eq.) in EtOH (2.0 mL) in a 10 mL microwave pressure vessel was added 1.5 equiv. phenylhydrazine hydrochloride derivatives **1** (0.108 mmol), and 1.0 equiv. TsOH (0.07 mmol). The reaction mixture was placed in a microwave at 130 °C and stirred for 3 h. After complete consumption of the starting material, the resulting solution was cooled to room temperature, treated with sat. aq. $Na_2CO_{3(aq.)}$ (10 mL) and extracted with EtOAc (2 x 10 mL). The combined organic extracts were dried over Na_2SO_4 and concentrated. The crude product was purified by preparative reversed-phase HPLC (C18, MeCN/H_2O gradient, 0.1% TFA), and lyophilized to afford **2** (32%-38%) and **3** (15-30%).

General Procedure for Gold-Catalyzed Pyridine Synthesis for Class 7

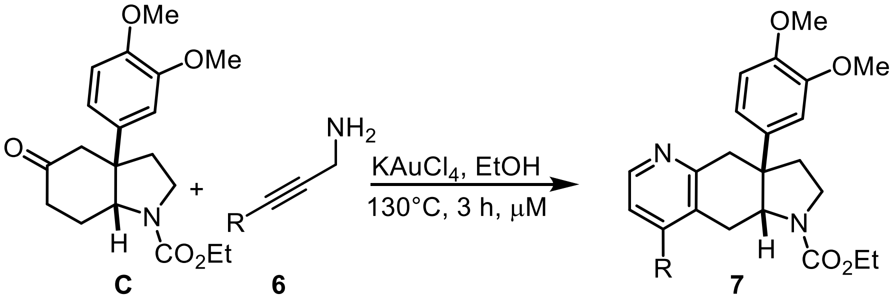

[3] A. Padwa, M. A. Brodney, K. Satake, C. S. Straub, *J. Org. Chem.* **1999**, *64*, 4617

To a solution of ketone **C** (25 mg, 0.07 mmol, 1 eq.) in EtOH (2.0 mL) in a 10 mL microwave pressure vessel added 1.5 equiv. of the propargylamine derivative **6** (0.108 mmol), and 0.1 equiv. of $KAuCl_4$ (0.007 mmol). The reaction mixture was placed in a microwave at 130 °C and stirred for 4 h under an inert argon atmosphere. The resulting suspension was concentrated *in vacuo*, dissolved in EtOAC, washed with Brine, and dried with Na_2SO_4. After concentration *in vacuo,* the crude product was purified by preparative reversed-phase HPLC (C18, MeCN/H_2O gradient, 0.1% TFA), and lyophilized to afford **7** (55%-62%).

Ethyl (3a,10a)-3a-(3,4-dimethoxyphenyl)-3,3a,4,5,10,10a-hexahydropyrrolo[3,2-*b*]carbazole-1(2*H*)-carboxylate (2a) and ethyl 10c-(3,4-dimethoxyphenyl)-1,3a,4,5,6,10c-hexahydropyrrolo[2,3-*c*]carbazole-3(2*H*)-carboxylate (3a)

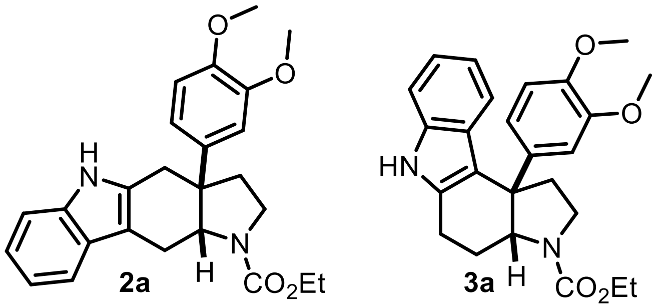

Following the general Fischer indole procedure, **2a** (18 mg, 68**%)** and **3a** (8.0 mg, 25%) were obtained as amorphous white solids in form of a TFA salt.

Data for **2a**

^{1}H NMR (500 MHz, $CDCl_3$) δ: 7.78 (1H, s, br), 7.48 (1H, d, *J* = 7.53 Hz), 7.30 (1H, *J* = 7.16 Hz), 7.10-7.16 (2H, m), 6.90-6.95 (2H, m), 6.82 (1H, d, *J* = 8.49 Hz), 4.67-4.71 (1H, m), 4.10-4.27 (2H, m), 3.87 (s, 3H), 3.85 (s, 3H), 3.34-3.58 (1H, m), 3.29-3.34 (1H, m), 3.21 (1H, d, *J* = 16.3 Hz), 3.07 (1H, d, *J* = 16.3 Hz), 2.71-2.07 (1H, m), 2.35-2.40 (1H, m), 2.31 (1H, dd, *J* = 9.3, 9.5 Hz), 1.24-1.29 (3H, m) **^{13}C NMR (176 MHz, $CDCl_3$) δ:** 155.6, 140.0, 147.6, 139.0, 136.6, 131.0, 127.2, 121.6, 119.5, 118.0, 117.5,

111.2, 110.5, 109.0, 106.3, 61.4, 59.0, 55.92, 55.86, 48.0, 43.4, 34.6, 33.8, 24.7, 14.7 **HRMS (ESI)** calcd for $C_{25}H_{28}N_2O_4$ [M + H]+ 421,2122 found 421,2107.

Data for **3a**

^{1}H NMR (700 MHz, $CDCl_3$) δ 7.89 (s, 1H), 7.33 (d, *J* = 8.1 Hz, 1H), 7.11 (dd, *J* = 7.9 Hz, 2H), 6.95 (dd, *J* = 7.4 Hz, 1H), 6.90 (d, *J* = 2.3 Hz, 1H), 6.86 – 6.80 (m, 1H), 6.73 (d, *J* = 8.6 Hz, 1H), 4.38 – 3.89 (m, 4H), 3.84 (s, 3H), 3.74 (s, 3H), 3.01 – 2.88 (m, 1H), 2.87 – 2.29 (m, 5H), 1.96 – 1.74 (m, 1H), 1.33 – 1.23 (m, 3H). **^{13}C NMR (176 MHz, $CDCl_3$) δ** 156.2, 148.8, 147.8, 136.5, 136.2, 135.9, 130.0, 127.8, 126.3, 121.4, 120.5, 120.1, 119.3, 111.0, 110.8, 66.2, 61.5, 56.0, 56.0, 50.8, 47.1, 36.0, 22.3, 18.6, 14.9. **HRMS (ESI)** calcd for $C_{25}H_{28}N_2O_4$ [M + H]+ 421.2122, found. 421.2104

Ethyl (3a,10a)-3a-(3,4-dimethoxyphenyl)-8-(trifluoromethoxy)-3,3a,4,5,10,10a-hexahydropyrrolo[3,2-*b*]carbazole-1(2*H*)-carboxylate (2b)

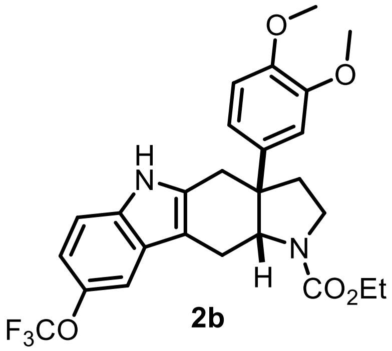

2b

Following the general Fischer indole procedure, **2b** (24 mg, 65%) was obtained as a white solid.

Data for **2b**

^{1}H NMR (600 MHz, $CDCl_3$) δ 8.19 – 7.81 (s, br, 1H), 7.32 (s, 1H), 7.23 (d, *J* = 8.6 Hz, 1H), 7.00 (d, *J* = 8.2 Hz, 1H), 6.96 – 6.86 (m, 2H), 6.81 (d, *J* = 8.4 Hz, 1H), 4.90 – 4.56

(m, 1H), 4.25 – 4.00 (m, 2H), 3.86 (s, 3H), 3.83 (s, 3H), 3.54 – 3.39 (m, 2H), 3.38 – 3.27 (m, 1H), 3.21 (d, *J* = 17.4 Hz, 1H), 3.05 (d, *J* = 17.4 Hz, 1H), 2.97 – 2.72 (m, 1H), 2.44 – 2.35 (m, 1H), 2.30 – 2.25 (m, 1H), 1.33 – 1.20 (m, 3H). **^{13}C NMR (151 MHz, $CDCl_3$) δ:** 155.6, 149.1, 147.0, 143.1, 138.0, 135.0, 133.5, 127.6, 121.0 (q, *J* = 255.3 Hz), 117.6, 115.5, 112.6, 111.1, 110.7, 109.1, 107.1, 61.5, 59.1, 56.0, 56.0, 48.1, 43.6, 34.8, 34.0, 24.9, 14.9. **HRMS (ESI)** calcd for $C_{26}H_{27}F_3N_2O_5$ [M + H]+ 505,1945 found 505,1919.

Ethyl (3a,10a)-8-(benzyloxy)-3a-(3,4-dimethoxyphenyl)-3,3a,4,5,10,10a-hexahydropyrrolo[3,2-*b*] carbazole-1(2*H*)-carboxylate (2c) and ethyl (3a,10c)-9-(benzyloxy)-10c-(3,4-dimethoxyphenyl)-1,3a,4,5,6,10c-hexahydropyrrolo[2,3-*c*]carbazole-3(2*H*)-carboxylate (3c)

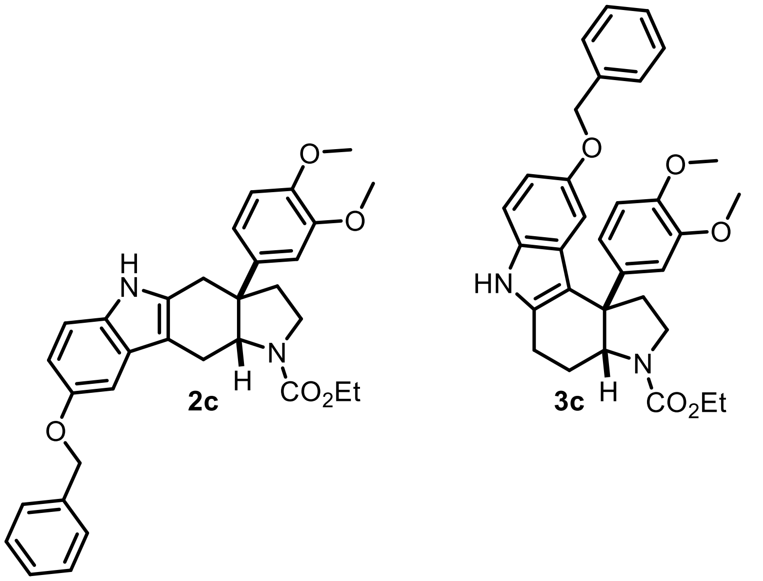

Following the general Fischer indole procedure, **2c** (17 mg, 44%) and **3c** (7.0 mg, 18%) were obtained as amorphous white solids.

Data for **2c**

^{1}H NMR (600 MHz, CDCl3) δ 7.61 (s, br, 1H), 7.48 (d, *J* = 7.4 Hz, 2H), 7.39 (dd, *J* = 7.6 Hz, 2H), 7.32 (dd, *J* = 7.4 Hz, 1H), 7.18 (d, *J* = 8.6 Hz, 1H), 7.04 (d, *J* = 2.3 Hz, 1H), 6.96 – 6.86 (m, 3H), 6.82 (d, *J* = 8.6 Hz, 1H), 5.11 (s, 2H), 4.80 – 4.57 (m, 1H), 4.21 – 4.04 (m, 2H), 3.87 (s, 3H), 3.84 (s, 3H), 3.55 – 3.37 (m, 2H), 3.37 – 3.24 (m, 1H), 3.19 (d, *J* = 17.3 Hz, 1H), 3.04 (d, *J* = 17.3 Hz, 1H), 2.97 – 2.67 (m, 1H), 2.43 – 2.35 (m, 1H), 2.34 – 2.25 (m, 1H), 1.29 – 1.20 (m, 3H). **^{13}C NMR (151 MHz, $CDCl_3$) δ** 153.7, 149.3, 148.0, 139.5, 138.1, 132.5, 132.1, 128.9 (2C), 128.8, 128.1, 128.0 (2C), 127.9 (2C), 117.9, 112.4, 111.5, 111.2, 109.4, 106.8, 102.3, 71.3, 61.4, 59.4, 56.3, 48.5, 43.8, 35.6, 34.1, 25.0, 15.2. **HRMS (ESI)** calcd for $C_{32}H_{34}N_2O_5S$ [M + H]+ 527.2540, found. 527.2516

Data for **3c**

^{1}H NMR (700 MHz, $CDCl_3$) δ 7.75 (s, br, 1H), 7.39 (s, 1H), 7.35 – 7.33 (m, 2H), 7.31 – 7.29 (m, 1H), 7.21 (d, *J* = 8.7 Hz, 1H), 7.02 – 6.99 (m, 1H) ,6.85 – 6.80 (m, 2H), 6.74 – 6.72 (m, 1H), 6.64 – 6.60 (m, 1H), 5.00 – 4.89 (m, 2H), 4.22 – 4.13 (m, 2H), 4.08 – 4.07 (m, 1 H), 3.88 – 3.85 (m, 4H), 3.73 (s, 3H), 2.93 – 2.91 (m, 1H), 2.73 – 2.72 (m, 2H), 2.67 – 2.65 (m, 1H) 2.64 – 2.63 (m, 1H), 2.43 – 2.40 (m, 1H), 2.26 – 2.27 (m, 1H), 1.83-1.82 (m, 1H), 1.27 (s, 3H).**^{13}C NMR (151 MHz, $CDCl_3$) δ** 152.8, 148.8, 147.8, 137.7, 136.7, 136.1, 131.8, 128.8, 128.6, 128.3, 127.9, 127.8, 127.6, 120.4, 116.4, 115.3, 114.6, 111.3, 111.0, 109.2, 104.7, 71.0, 66.3, 61.3, 56.0 (2C), 50.8, 47.0, 35.7, 22.2, 18.7, 14.9 **HRMS (ESI)** calcd for $C_{32}H_{34}N_2O_5S$ [M + H]+ 527.2540, found. 527.2516

Ethyl (3a,10a)-3a-(3,4-dimethoxyphenyl)-6-methoxy-3,3a,4,5,10,10a-hexahydropyrrolo[3,2-*b*]carbazole-1(2*H*)-carboxylate (2d) and Ethyl-3a-(3,4-dimethoxyphenyl)-8-sulfamoyl-3,3*a*,4,5,10,10a hexahydropyrrolo[3,2-*b*]carbazole-1(2*H*)-carboxylate (2d)

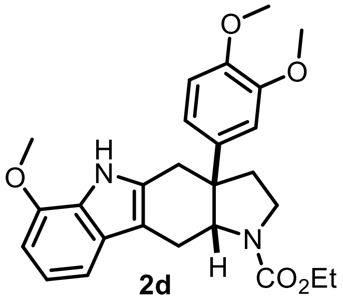

Following the general Fischer indole procedure, **2d** (13 mg, 40%) was obtained as a white amorphous solid.

^{1}H NMR (600 MHz, CDCl$_3$) δ 7.91 (s, br, 1H), 7.10 (d, *J* = 7.7 Hz, 1H), 7.04 – 6.98 (m, 1H), 6.95 – 6.90 (m, 2H), 6.82 (d, *J* = 8.2 Hz, 1H), 6.64 (d, *J* = 7.4 Hz, 1H), 4.69 – 4.62 (m, 1H), 4.19 – 4.10 (m, 2H), 3.94 (s, 3H), 3.87 (s, 3H), 3.84 (s, 3H), 3.51 – 3.43 (m, 2H), 3.34 – 3.27 (m, 1H), 3.21 (d, *J* = 17.2 Hz, 1H), 3.07 (d, *J* = 17.2 Hz, 1H), 2.91 – 2.76 (m, 1H), 2.41 – 2.37 (m, 1H), 2.32 – 2.28 (m, 1H), 1.28 – 1.22 (m, 3H). **^{13}C NMR (151 MHz, CDCl$_3$) δ** 155.6, 148.7, 147.7, 145.8, 139.3, 130.9, 126.8, 121.8, 120.1, 119.7, 117.7, 111.3, 111.0, 109.2, 102.1, 61.2, 59.2, 56.1, 56.0, 55.5, 48.1, 43.5, 35.3, 34.0, 25.2, 14.9. **HRMS (ESI)** calcd. for $C_{26}H_{30}N_3O_6S$ [M + H]+ 451,2227, found 451,2209.

Ethyl-3a-(3,4-dimethoxyphenyl)-8-sulfamoyl-3,3*a*,4,5,10,10a hexahydropyrrolo[3,2-*b*]carbazole-1(2*H*)-carboxylate (2e)

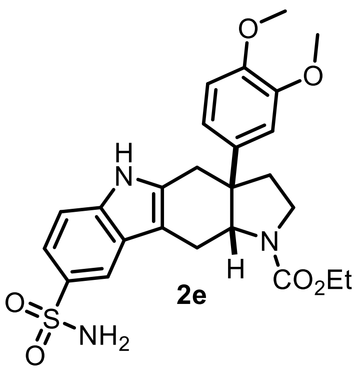

Following the general Fischer indole procedure, **2e** (22 mg, 65%) was obtained as an amorphous white solid.

Data for **2e**

^{1}H NMR (700 MHz, CDCl3) δ 8.75 (s, 1H), 8.15 – 7.93 (m, 1H), 7.68 – 7.50 (m, 1H), 7.26 – 7.17 (m, 1H), 6.92 – 6.81 (m, 2H), 6.78 (d, *J* = 8.3 Hz, 1H), 5.21 (s, 2H), 4.71 – 4.47 (m, 1H), 4.28 – 4.00 (m, 2H), 3.82 (s, 3H), 3.79 (s, 3H), 3.52 – 3.25 (m, 3H), 3.19 (d, *J* = 17.5 Hz, 1H), 3.13 – 2.94 (m, 1H), 2.91 – 2.72 (m, 1H), 2.42 – 2.26 (m, 1H), 2.27 – 2.09 (m, 1H), 1.35 – 1.14 (m, 3H). **^{13}C NMR (176 MHz, $CDCl_3$) δ** 155.7, 149.1, 147.8, 138.8, 138.7, 134.4, 132.7, 126.8, 119.3, 117.6, 117.5, 111.4, 111.2, 109.2, 107.6, 61.5, 58.9, 56.1, 56.0, 47.8, 43.6, 35.4, 33.7, 24.7, 14.9. **HRMS (ESI)** calcd for $C_{25}H_{29}N_3O_6S$ [M + H]+ 500,1850, found 500,1823.

Ethyl-3a-(3,4-dimethoxyphenyl)-8-bromo-3,3*a*,4,5,10,10*a*-hexahydropyrrolo[3,2-*b*]carbazole-1(2H)-carboxylate (2f) and Ethyl 9-bromo-10c-(3,4-dimethoxyphenyl)-1,3a,4,5,6,10c-hexahydropyrrolo[2,3-*c*]carbazole-3(2*H*)-carboxylate (3f)

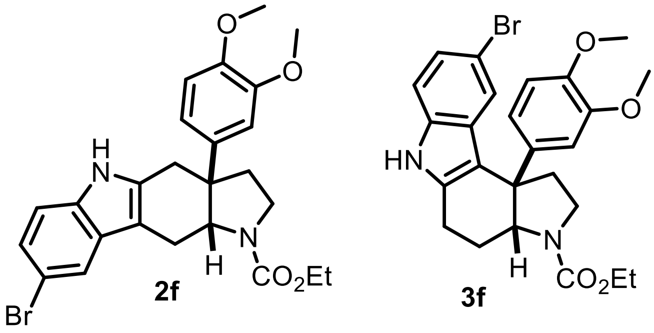

Following the general Fischer indole procedure, **2f** (22 mg, 60%) and **3f** (9.0 mg, 24%) were obtained as white amorphous solids.

Data for **2f**

^{1}H NMR (700 MHz, $CDCl_3$) δ 7.78 (s, br, 1H), 7.60 (s, 1H), 7.22 (d, *J* = 8.0 Hz, 1H), 7.15 (d, *J* = 8.5 Hz, 1H), 6.95 – 6.87 (m, 2H), 6.82 (d, *J* = 8.4 Hz, 1H), 4.83 – 4.55 (m, 1H), 4.25 – 4.05 (m, 2H), 3.87 (s, 3H), 3.84 (s, 3H), 3.50 – 3.45 (m, 2H), 3.35 – 3.28 (m, 1H), 3.20 (d, *J* = 17.3 Hz, 1H), 3.05 (d, *J* = 17.4 Hz, 1H), 2.96 – 2.68 (m, 1H), 2.44 – 2.35 (m, 1H), 2.30 – 2.23 (m, 1H), 1.31 – 1.20 (m, 3H). **^{13}C NMR (176 MHz, $CDCl_3$) δ** 155.7, 149.1, 147.9, 138.9, 135.3, 132.8, 129.2, 124.5, 120.8, 117.6, 112.9, 112.0, 111.3, 109.1, 106.5, 61.4, 59.1, 56.1, 56.0, 47.8, 43.6, 35.3, 33.8, 24.4, 14.9 **HRMS (ESI)** calc for $C_{25}H_{27}BrN_2O_4$ [M+Na]+ calcd. 521.1046 found 521.1043.

Data for **3f**

^{1}H NMR (700 MHz, CDCl3) δ 7.92 (s, br, 1H), 7.19-7.24 (m, 3H), 6.86 (s,1H), 6.78-6.75 (m, 2H), 4.08 – 4.22 (m, 3H), 3.87 (s, 3), 3.76 (s, 3H), 2.89 – 2.76 (m, 1H), 2.74 –

2.66 (m, 4H), 2.44 – 2.42 (m, 2H), 1.83-1.81 (m, 1H), 1.26 (t, J = 7.0 Hz, 3H). **^{13}C NMR (151 MHz, CDCl$_3$) δ** 156.1, 155.6, 152.3, 148.9, 148.0, 137.6, 135.7, 135.1, 125.0, 124.2, 122.4, 120.4, 112.7, 112.2, 110.9, 65.9, 61.3, 56.1, 56.0, 50.7, 47.0, 36.0, 22.1, 18.6, 14.9. (*177.1 = Trifluoracetic acid residue from prep. HPLC conditions*) **HRMS (ESI)** calcd for $C_{25}H_{27}BrN_2O_4$ [M + Na]+ 521.1046 found 521.1048

Ethyl (3a,10a)-6-bromo-3a-(3,4-dimethoxyphenyl)-3,3a,4,5,10,10a-hexahydropyrrolo[3,2-*b*]carbazole-1(2*H*)-carboxylate (2g) and ethyl 9-bromo-10c-(3,4-dimethoxyphenyl)-1,3a,4,5,6,10c-hexahydropyrrolo[2,3-*c*]carbazole-3(2*H*)-carboxylate (3g)

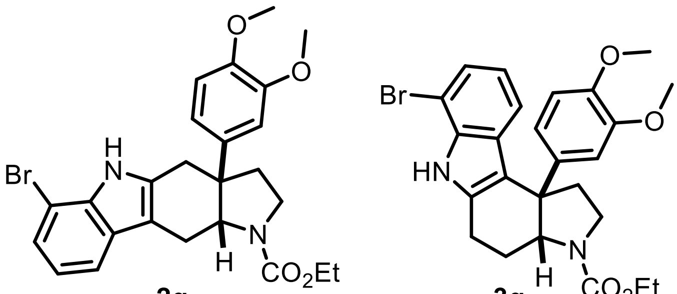

2g **3g**

Following the general Fischer indole procedure, **2g** (16 mg, 42%) and **3g** (8.0 mg, 22%) were obtained as yellow amorphous solids.

Data for **2g**

^{1}H NMR (500 MHz, CDCl$_3$) δ 7.93 (s, br, 1H), 7.41 (d, J = 7.8 Hz, 1H), 7.29 (d, J = 7.6 Hz, 1H), 7.03 – 6.86 (m, 3H), 6.83 (d, J = 8.3 Hz, 1H), 4.73 – 4.56 (m, 1H), 4.23 – 4.08 (m, 2H), 3.87 (s, 3H), 3.85 (s, 3H), 3.53 – 3.42 (m, 2H), 3.38 – 3.29 (m, 1H), 3.25 (d, J = 17.5 Hz, 1H), 3.10 (d, J = 17.5 Hz, 1H), 3.00 – 2.74 (m, 1H), 2.44 – 2.36 (m, 1H), 2.28 (dd, J = 12.7, 8.8 Hz, 1H), 1.33 – 1.18 (m, 3H). **^{13}C NMR (126 MHz, CDCl$_3$) δ** 155.6, 149.2, 147.9, 138.8, 135.3, 132.1, 128.6, 124.1, 120.9, 117.6, 117.3, 111.4, 109.1, 107.7, 104.3, 61.5, 59.1, 56.1, 56.0, 47.8, 43.6, 35.4, 33.8, 24.7, 14.9. **HRMS (ESI)** $C_{25}H_{27}BrN_2O_4$ [M+Na]+ calcd. 499.1127 found 499.1198.

Data for **3g**

^{1}H NMR (500 MHz, CDCl3) δ 8.07 (s, br, 1H), 7.25 (d, *J* = 1.0 Hz, 1H), 7.09 – 6.98 (m, 1H), 6.94 – 6.67 (m, 4H), 4.26 – 3.97 (m, 3H), 3.90 – 3.71 (m, 7H), 3.05 – 2.23 (m, 6H), 1.26 – 1.24 (m, 3H). **^{13}C NMR (126 MHz, $CDCl_3$) δ** 156.1, 148.9, 147.9, 137.0, 135.9, 135.1, 127.6, 124.1, 123.8, 120.6, 120.4, 119.2, 112.5, 110.9, 104.5, 66.1, 61.1, 56.1, 56.0, 50.6, 46.7, 36.2, 21.5, 18.6, 14.9 **HRMS (ESI)** calcd for $C_{25}H_{27}BrN_2O_4$ [M + H]+ 499.1196, found. 499.1227 **Note:** In **3g,** the missing methylene proton signal is completely overlapped by residual HDO at 1.86 ppm. The assignment is supported by HSQC and consistent with the other chemical shifts of the compound series.

Ethyl 8-chloro-3a-(3,4-dimethoxyphenyl)-3,3a,4,5,10,10a-hexahydropyrrolo[3,2-*b*]carbazole-1(2*H*)-carboxylate (2h) and ethyl 9-bromo-10c-(3,4-dimethoxyphenyl)-1,3a,4,5,6,10c-hexahydropyrrolo[2,3-c]carbazole-3(2*H*)-carboxylate (3h)

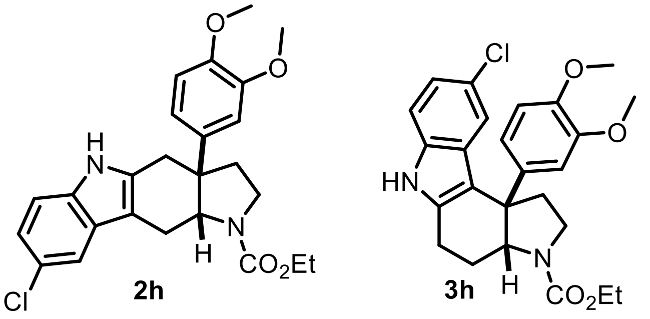

Following the general Fischer indole procedure, **2c** (20 mg, 62%) and **3c** (10 mg, 30%) as obtained as a white amorphous solid.

Data for **2h**

^{1}H NMR (700 MHz, CDCl3) δ 7.85 (s, 1H), 7.48 – 7.37 (m, 1H), 7.18 (d, *J* = 8.5 Hz, 1H), 7.09 (d, *J* = 8.5 Hz, 1H), 6.93 – 6.85 (m, 2H), 6.81 (d, *J* = 8.5 Hz, 1H), 4.77 – 4.50 (m, 1H), 4.28 – 4.07 (m, 2H), 3.86 (s, 3H), 3.83 (s, 3H), 3.52 – 3.36 (m, 2H), 3.38 –

3.26 (m, 1H), 3.19 (d, *J* = 17.4 Hz, 1H), 3.05 (d, *J* = 17.4 Hz, 1H), 2.94 – 2.70 (m, 1H), 2.45 – 2.34 (m, 1H), 2.27 (dd, *J* = 12.7, 8.8 Hz, 1H), 1.33 – 1.19 (m, 3H). **^{13}C NMR (176 MHz, CDCl$_3$) δ** 155.7, 149.1, 147.8, 138.9, 135.0, 132.9, 128.5, 125.3, 121.9, 117.8, 117.6, 111.6, 111.3, 109.1, 106.5, 61.7, 59.1, 56.1, 56.0, 48.1, 43.6, 35.2, 34.0, 24.9, 14.9 **HRMS (ESI)** calcd. for $C_{25}H_{27}ClN_2O_4$ 455,1732 found 455,1705.

Data for **3h**

^{1}H NMR (700 MHz, CDCl$_3$) δ 7.90 (s, 1H), 7.23 (d, *J* = 8.5 Hz, 1H), 7.09 – 7.03 (m, 2H), 6.86 (d, *J* = 2.2 Hz, 1H), 6.79 – 6.72 (m, 2H), 4.27 – 3.98 (m, 3H), 3.89 – 3.72 (m, 7H), 2.98 – 2.33 (m, 6H), 1.85-1.80 (m, 1H), 1.26 – 1.25 (m, 3H). **^{13}C NMR (176 MHz, CDCl$_3$) δ** 155.6, 148.9, 147.9, 137.8, 135.8, 134.8, 127.5, 125.1, 121.7, 121.6, 120.5, 120.3, 119.4, 111.0, 110.9, 66.0, 61.2, 56.1, 56.0, 50.4, 47.0, 35.9, 22.1, 18.7, 14.9. **HRMS (ESI)** $C_{25}H_{27}ClN_2O_4$ calc. 455,1738 found 455,1713

Ethyl (3a,10a)-6,8-dichloro-3a-(3,4-dimethoxyphenyl)-3,3a,4,5,10,10a-hexahydropyrrolo[3,2-*b*]carbazole-1(2*H*)-carboxylate (2i) and ethyl (3a,10c)-7,9-dichloro-10c-(3,4-dimethoxyphenyl)-1,3a,4,5,6,10c-hexahydropyrrolo[2,3-*c*]carbazole-3(2*H*)-carboxylate (3i)

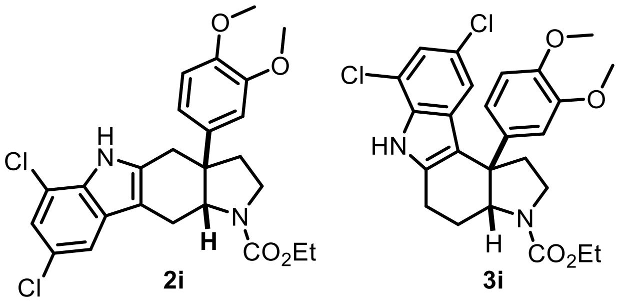

Following the general Fischer indole procedure, **2i** (18 mg, 51%) and **3i** (6.0 mg,18%) was obtained as yellow amorphous solid.

Data for **2i**

^{1}H NMR (600 MHz, $CDCl_3$) δ 8.03 (s, br, 1H), 7.41 – 7.30 (m, 1H), 7.19 – 7.09 (m, 1H), 6.91 – 6.88 (m, 2H), 6.82 (d, J = 8.4 Hz, 1H), 4.75 – 4.53 (m, 1H), 4.26 – 4.06 (m, 2H), 3.86 (s, 3H), 3.84 (s, 3H), 3.54 – 3.28 (m, 3H), 3.22 (d, J = 17.5 Hz, 1H), 3.08 (d, J = 17.5 Hz, 1H), 2.69 – 2.67 (m, 1H), 2.46 – 2.32 (m, 1H), 2.42 – 2.26 (m, 1H), 1.30 – 1.23 (m, 3H). **^{13}C NMR (151 MHz, $CDCl_3$) δ** 154.6, 148.2, 146.9, 137.6, 132.8, 131.4, 128.3, 124.3, 120.1, 116.6, 115.6, 115.4, 110.4, 108.1, 106.7, 60.4, 58.0, 55.1 (2C), 46.8, 42.7, 34.5, 33.1, 23.4, 13.9. **HRMS (ESI)** $C_{25}H_{26}Cl_2N_2O_4$ [M+Na]+ calc. 489.1342 found 489.1314

Data for **3i**

^{1}H NMR (700 MHz, $CDCl_3$) δ 8.11 (s, br, 1H), 7.12 (s, 1H), 6.99 – 6.94 (m, 1H), 6.84 (s, 1H), 6.76 – 6.74 (m, 2H), 4.14 – 3.98 (m, 3H), 3.86-3.76 (m, 7H), 2.89-2.78 (m, 1H), 2.78 – 2.73 (m, 1H), 2.70-2.68 (m, 2H), 2.44 – 2.37 (m, 2H), 1.83 (m, 1H), 1.27 (t, J = 7.2 Hz, 3H). **^{13}C NMR (176 MHz, $CDCl_3$) δ** 149.2, 148.3, 138.8, 135.7, 132.4, 128.5, 125.3, 121.2, 120.6, 120.4, 118.3, 116.9, 112.6, 111.3, 110.1, 66.2, 61.3, 56.2 (2C), 50.7, 46.8, 36.2 22.9, 18.9, 15.1. $C_{25}H_{26}Cl_2N_2O_4$ [M+Na]+ calc. 489.1342 found 489.1307

Ethyl (3a,10a)-6,9-dichloro-3a-(3,4-dimethoxyphenyl)-3,3a,4,5,10,10a-hexahydropyrrolo[3,2-*b*]carbazole-1(2*H*)-carboxylate (2j)

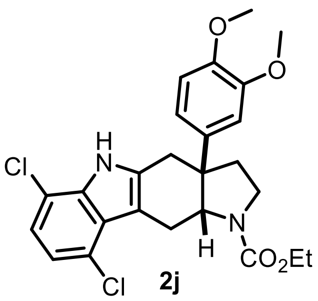

2j

Following the general Fischer indole procedure, **2j** (14 mg, 40%) were obtained as a yellow oil.

Data for **2j**

^{1}H NMR (700 MHz, $CDCl_3$) δ 8.00 (s, 1H), 7.03 – 6.95 (m, 2H), 6.95 – 6.87 (m, 2H), 6.83 (d, *J* = 8.4 Hz, 1H), 4.81 – 4.43 (m, 1H), 4.23 – 4.06 (m, 2H), 3.92 – 3.85 (m, 7H), 3.54 – 3.46 (m, 1H), 3.41 – 3.27 (m, 1H), 3.23 (d, *J* = 17.4 Hz, 1H), 3.20 – 3.01 (m, 2H), 2.43 – 2.27 (m, 2H), 1.31 – 1.22 (m, 3H).**^{13}C NMR (176 MHz, $CDCl_3$) δ** 155.5, 149.2, 147.9, 138.7, 134.6, 133.0, 125.8, 124.6, 121.4, 120.9, 117.7, 114.7, 111.4, 109.1, 108.2, 61.4, 59.0, 56.1, 56.0, 47.2, 43.6, 35.4, 34.4, 26.2, 14.9 **HRMS (ESI)** $C_{25}H_{26}Cl_2N_2O_4$ [M+Na]+ calc. 488.1270 found 511.1165

Ethyl (3a,10a)-3a-(3,4-dimethoxyphenyl)-8-fluoro-3,3a,4,5,10,10a-hexahydropyrrolo[3,2-*b*]carbazole-1(2*H*)-carboxylate (2k)

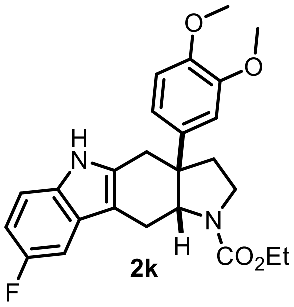

2k

Following the general Fischer indole procedure, **2k** (10 mg, 32%) was obtained as a colorless oil.

Data for **2k**

^{1}H NMR (700 MHz, CDCl3) δ 7.69 (s, br, 1H), 7.19 (dd, *J* = 8.7, 4.2 Hz, 1H), 7.12 (d, *J* = 9.4 Hz, 1H), 6.93 (d, *J* = 8.3 Hz, 1H), 6.91 – 6.85 (m, 2H), 6.82 (d, *J* = 8.4 Hz, 1H), 4.98 – 4.58 (m, 1H), 4.32 – 4.03 (m, 2H), 3.85 (d, *J* = 19.2 Hz, 7H), 3.56 – 3.26 (m, 3H), 3.21 (d, *J* = 17.3 Hz, 1H), 3.06 (d, *J* = 17.3 Hz, 1H), 2.97 – 2.70 (m, 1H), 2.46 – 2.38 (m, 1H), 2.34 – 2.21 (m, 1H), 1.28 – 1.22 (m, 3H). **^{13}C NMR (176 MHz, $CDCl_3$) δ** 158.7, 155.3, 149.1, 147.8, 139.1, 133.4, 133.1, 131.0, 127.8, 117.7, 111.3, 111.0, 109.8, 109.2, 103.4, 61.2, 59.0, 56.1, 56.0, 50.7, 43.6, 35.5, 34.0, 24.7, 14.9. **HRMS (ESI)** calcd for $C_{25}H_{28}FN_2O_4$+ [M + H]+ 439.2028, found 439,2025.

Ethyl (3a,10a)-3a-(3,4-dimethoxyphenyl)-8-(hydroxy-l2-methoxy)-3,3a,4,5,10,10a-hexahydropyrrolo[3,2-*b*]carbazole-1(2H)-carboxylate (2l)

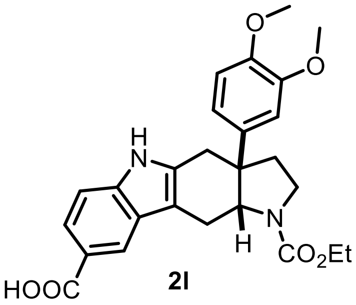

2l

Following the general Fischer indole procedure, **2l** (18 mg, 55%) was obtained as a yellow amorphous solid.

Data for **2l**

^{1}H NMR (600 MHz, $CDCl_3$) δ 8.25 – 8.32 (m, 1H, aromatic H, two isomers), 8.00 – 7.29 (m, 3H, mixture of isomers), 6.94–6.92 (m, 2H), 6.81 (dd, *J* = 8.5, 2.6 Hz, 1H), 4.66 – 4.65 (m, 1H), 4.43–4.14 (m, 2H, each isomer has two protons), 4.14 (m, 1H), 3.87 (m, 3H), 3.84 (m, 3H), 3.53–3.46 (m, 2H), 3.31–3.29 (m, 1H), 3.24 (d, *J* = 17.4 Hz, 1H), 3.08 (d, *J* = 17.4 Hz, 1H), 3.04–2.78 (m, 1H), 2.45–2.36 (m, 1H), 2.35–2.23 (m, 1H), 1.42 – 1.21 (m, 3H, ethyl group, isomers). **^{13}C NMR (151 MHz, $CDCl_3$) δ 168.4** (171.4, 167.9, 166.0), 155.7, 149.1, 147.9, **139.4** (139.9, 139.4, 139.0), 132.7, 131.1, 129.1, 128.8, 127.1, 123.4, 120.9, 117.6, 111.3, 110.2, 109.1, **61.4**, 60.8, 59.0, 56.1, 56.0, 47.9, 43.6, 34.8, 24.7, **14.6** (14.9, 14.6, 14.4). **HRMS (ESI)** calcd for $C_{26}H_{29}N_2O_6$+ [M + H]+ 465,2020, found 465,1998

Note: The carboxylic acid shows intramolecular interaction with an ethylcarbamate rotamer, indicated by the broadened methylene signal at 61.4 (^{13}C NMR) and three distinct methyl group signals. The carbonyl (168.4) and aromatic carbons (139.4) have similar signal ratios, appearing in triplicate, likely due to slow conformer dynamics. In the 1H-NMR, the protons of the ethyl group and the carboxylic acids corresponding aryl protons also appear in triplicate. The Carboxylic acid proton was not detected in the ^{1}H-NMR.

Ethyl (7a,10a)-10a-(3,4-dimethoxyphenyl)-7a,9,10,10a,11,12 hexahydrobenzo[a]pyrrolo[2,3-*h*]carbazole-8(7*H*)-carboxylate (2m) and ethyl (3a,12c)-12c-(3,4-dimethoxyphenyl)-1,3a,4,5,6,12 hexahydrobenzo[*a*]pyrrolo[3,2-*g*]carbazole-3(2*H*)-carboxylate (3m)

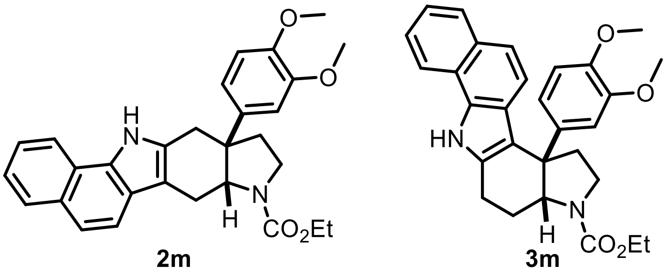

2m **3m**

Following the general Fischer indole procedure, **2m** (14 mg, 40%) and **3m** (11 mg, 33%) were obtained as a yellow oil.

Data for **2m**

^{1}H NMR (500 MHz, CDCl3) δ 8.48 (s, br, 1H), 7.93 (dd, *J* = 8.1 Hz, 2H), 7.61 (d, *J* = 8.6 Hz, 1H), 7.57 – 7.44 (m, 2H), 7.43 – 7.34 (m, 1H), 7.05 – 6.90 (m, 2H), 6.83 (d, *J* = 8.4 Hz, 1H), 4.80 – 4.53 (m, 1H), 4.25 – 4.04 (m, 2H), 3.86 (s, 6H), 3.54 (d, *J* = 17.1 Hz, 3H), 3.33 (d, *J* = 17.1 Hz, 1H), 3.18 – 3.16 (m, 1H), 3.09 – 2.80 (m, 1H), 2.51 – 2.19 (m, 2H), 1.32 – 1.17 (m, 3H). **^{13}C NMR (126 MHz, $CDCl_3$) δ** 155.5, 149.1, 147.8, 144.3, 139.4, 131.0, 130.4, 129.5, 129.2, 125.5, 123.6, 122.9, 121.6, 120.4, 119.3, 118.6, 117.7, 111.3, 109.2, 61.2, 59.3, 56.1, 56.0, 48.0, 43.6, 34.1, 29.9, 25.2, 14.9 **HRMS (ESI)** calcd. for $C_{29}H_{30}N_2O_4$+ [M + H]+ 471,2278, found 471,2282

Data for **3m**

^{1}H NMR (600 MHz, CDCl3) δ 8.83 – 8.67 (m, 1H), 8.00 (d, *J* = 8.2 Hz, 1H), 7.87 (d, *J* = 8.1 Hz, 1H), 7.51 (ddd, *J* = 8.2, 6.9, 1.2 Hz, 1H), 7.44 – 7.31 (m, 2H), 7.26 – 7.17 (m, 1H), 6.92 (d, *J* = 2.2 Hz, 1H), 6.86 (d, *J* = 8.3 Hz, 1H), 6.74 (d, *J* = 8.4 Hz, 1H), 4.30 – 3.95 (m, 3H), 3.84 (s, 3H), 3.70 (s, 3H), 2.94 – 2.64 (m, 4H), 2.60 – 2.32 (m, 3H), 1.92 – 1.80 (m, 1H), 1.40 – 1.19 (m, 3H). **^{13}C NMR (151 MHz, $CDCl_3$) δ** 156.2, 149.0, 148.0, 136.8, 134.4, 131.0, 130.2, 129.3, 125.8, 124.0, 122.3, 121.8, 120.6, 120.4, 120.2, 119.5, 113.3, 111.4, 111.1, 66.5, 61.5, 56.3, 56.2, 51.1, 47.2, 36.9, 22.6, 18.4, 13.7 HRMS **(ESI)** $C_{29}H_{30}N_2O_4$ [M+H]+ calc. 471,2278 found 471,2244

Ethyl 10c-(3,4-dimethoxyphenyl)-9-methoxy-1,3a,4,5,6,10c-hexahydropyrrolo[2,3-*c*]carbazole-3(2*H*)-carboxylate (3n)

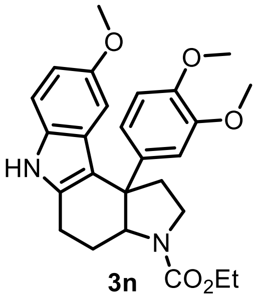

3n

Following the general Fischer indole procedure, **3o** (5.0 mg, 15%) was obtained as an orange oil.

Data for **3n**

^{1}H-NMR (700 MHz, CDCl3) δ 7.75 (s, br, 1H), 7.21 (d, *J* = 8.7 Hz, 1H), 6.99 – 6.68 (m, 4H), 6.56-6.54 (m, 1H), 4.24 – 3.99 (m, 3H), 3.91 – 3.79 (m, 4H), 3.78 – 3.62 (m, 6H), 3.08 – 2.26 (m, 6H), 1.97 – 1.67 (m, 1H), 1.31 – 1.22 (m, 3H). **^{13}C NMR (176 MHz, CDCl3) δ** 155.8, 153.6, 148.8, 147.8, 136.9, 136.2, 131.6, 129.1, 128.0, 127.0, 120.6,

111.3, 110.9, 110.5, 103.1, 66.3, 61.4, 56.1, 56.0, 51.0, 47.3, 46.8, 35.8, 22.4, 18.7, 14.8. **HRMS (ESI)** calcd for $C_{26}H_{30}N_2O_5$ [M + H]+ 451.2227, found. 451.2211

Ethyl (3a,10a)-3a-(3,4-dimethoxyphenyl)-3,3a,4,5,10,10a-hexahydropyrido[2,3-*b*]pyrrolo[2,3-*f*]indole-1(2*H*)-carboxylate (5)

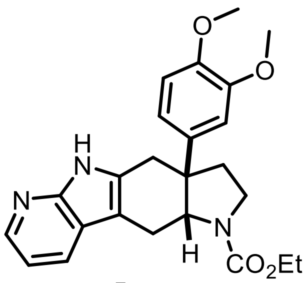

5

To a solution of ketone **C** (25 mg, 0.07 mmol, 1 eq.) in Diethylene glycol (2.0 mL) in a 10 mL microwave pressure vessel was added 1.5 equiv. 2-hydrazinylpyridine hydrochloride **4** (16 mg, 0.105 mmol). The reaction mixture was placed in a microwave at 220 °C and stirred for 4 h. After complete consumption of the starting material, the resulting solution was cooled to room temperature, treated with sat. aq. $Na_2CO_{3(aq.)}$ (10 mL) and extracted with EtOAc (2 x 10 mL). The combined organic extracts were dried over Na_2SO_4 and concentrated *in vacuo*. The crude product was purified by preparative reversed-phase HPLC (C18, MeCN/H_2O gradient, 0.1% TFA), yielding **5** (11 mg, 35%) as a colorless oil.

Data for **5**

^{1}H NMR (700 MHz, CDCl3) δ 8.27 – 8.11 (m, 1H), 8.13 – 8.01 (m, 1H), 7.28 (dd, *J* = 7.8, 5.8 Hz, 1H), 6.89 – 6.84 (m, 2H), 6.81 (d, *J* = 8.2 Hz, 1H), 4.78 – 4.51 (m, 1H), 4.27 – 4.04 (m, 2H), 3.93 – 3.81 (m, 6H), 3.48 (ddd, *J* = 11.1, 8.1, 4.0 Hz, 1H), 3.47 – 3.30 (m, 3H), 3.28 – 2.82 (m, 2H), 2.55 – 2.29 (m, 1H), 2.21 (dt, *J* = 12.6, 8.1 Hz, 1H), 1.35 – 1.20 (m, 3H). **^{13}C NMR (176 MHz, $CDCl_3$) δ** 155.7, 149.2, 148.3, 148.1, 143.7,

137.7, 137.4, 132.7, 117.7, 117.4, 115.7, 114.8, 111.4, 109.0, 61.3, 58.5, 56.1, 56.0, 43.8, 36.4, 34.8, 33.6, 24.0, 14.9. **HRMS (ESI)** $C_{24}H_{27}N_3O_4$ [M+H]+ calc. 422,2080 found 422,2057

Ethyl (3a,9a)-3a-(3,4-dimethoxyphenyl)-2,3,3a,4,9,9a-hexahydro-1H-pyrrolo[2,3-*g*]quinoline-1-carboxylate (7a) and ethyl (3a,9a)-3a-(3,4 dimethoxyphenyl)-8-methyl-2,3,3a,4,9,9a-hexahydro-1*H*-pyrrolo[2,3-*g*]quinoline-1-carboxylate (7b)

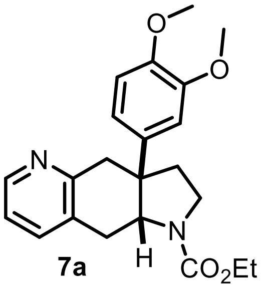

7a

Following the general gold-catalyzed cyclization procedure, **7a** (16 mg, 60%) was obtained as a colorless oil.

Data for **7a**

^{1}H NMR (700 MHz, CDCl$_3$) δ 8.87 – 8.46 (m, 1H), 8.09 – 7.75 (m, 1H), 7.72 – 7.40 (m, 1H), 6.81 (d, *J* = 8.3 Hz, 1H), 6.66 (dd, *J* = 8.4, 2.2 Hz, 1H), 6.62 – 6.61 (m, 1H), 4.31 – 4.05 (m, 3H), 3.90 – 3.73 (m, 7H), 3.53 – 3.22 (m, 3H), 2.85-2.95 (m, 1H), 2.36 – 2.14 (m, 3H), 1.24 (t, *J* = 7.1 Hz, 3H). **^{13}C NMR (176 MHz, CDCl$_3$) δ HRMS (ESI)** 154.9, 152.7, 149.7, 149.0, 146.0, 142.8, 140.2, 135.4, 124.5, 120.0, 111.4, 110.5, 63.9, 61.7, 56.3, 56.1, 52.8, 45.0, 36.7, 24.7, 23.3, 14.8. **$C_{22}H_{26}N_2O_4$** [M+Na]+ calc. 405,1785 found 405.1783

Ethyl-(3a,9a)-3a-(3,4-dimethoxyphenyl)-8-methyl-2,3,3a, 4,9,9a – hexahydropyrrolo-1H-pyrorolo[2,3-*g*]quinoline-1-carboxylate (7b)

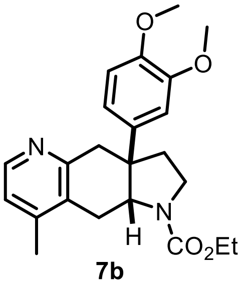

7b

Following the general gold-catalyzed cyclization procedure, **7b** (18 mg, 62%) was obtained as an amorphous white solid.

Data for **7b**

^{1}H NMR (700 MHz, CDCl3) δ 8.44 (d, *J* = 5.7 Hz, 1H), 7.43 – 7.29 (m, 1H), 6.87 – 6.82 (m, 1H), 6.79 – 6.75 (m, 2H), 4.71 – 4.46 (m, 1H), 4.19 – 4.09 (m, 2H), 3.86 (s, 3H), 3.84 (s, 3H), 3.57 – 3.49 (m, 3H), 3.24 – 3.16 (m, 2H), 2.49 (s, 3H), 2.30 – 2.26 (m, 1H), 2.15 – 2.10 (m, 1H), 1.31 – 1.21 (m, 3H). **^{13}C NMR (176 MHz, $CDCl_3$) δ** 155.6, 153.2, 149.5, 148.2, 139.4, 136.7, 133.8, 125.4, 117.7, 116.0, 111.3, 109.4, 61.3, 61.1, 56.2, 56.0, 49.1, 45.4, 38.4, 29.7, 28.5, 20.0, 14.9. **HRMS (ESI)** $C_{23}H_{29}N_2O_4$ (M+H) 397,2127 found 397,2104

Ethyl (3a,9a)-3a-(3,4-dimethoxyphenyl)-8-methyl-2,3,3a,4,9,9a-hexahydro-1*H*-pyrrolo[2,3-*g*]quinoline-1-carboxylate (7c)

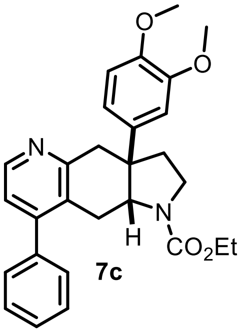

Following the general gold-catalyzed cyclization procedure, **7c** (20 mg, 65%) was obtained as an amorphous white solid.

Data for **7c**

^{1}H NMR (700 MHz, CDCl3) δ 8.84 – 8.27 (m, 1H), 7.71 – 7.43 (m, 4H), 7.40 – 7.33 (m, 2H), 6.86 – 6.74 (m, 3H), 4.64 – 4.37 (m, 1H), 4.17 – 3.97 (m, 2H), 3.88 – 3.84 (m, 6H), 3.69 – 3.06 (m, 6H), 2.46 – 2.02 (m, 3H), 1.29 – 0.97 (m, 3H). **^{13}C NMR (176 MHz, $CDCl_3$) δ** 157.2, 155.4, 154.7, 149.4, 148.2, 138.5, 137.11, 135.3, 133.9, 130.5, 129.4, 129.4, 128.7, 125.4, 117.7, 111.3, 109.3, 109.2, 61.7, 56.0, 50.5, 49.3, 45.4, 39.1, 36.2, 30.1, 26.0, 14.9. **HRMS (ESI)** $C_{28}H_{31}N_2O_4$ (M+H) calc. 459,2284 found 459,2259

.

Ethyl (3a,11a)-7-bromo-3a-(3,4-dimethoxyphenyl)-2,3,3a,4,11,11a-hexahydro-1*H*-pyrrolo[2,3-*b*]acridine-1-carboxylate (9)

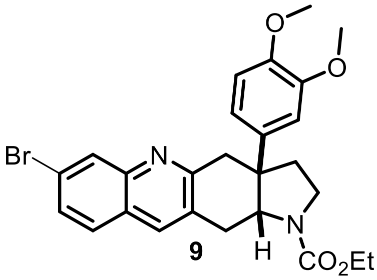

9

Procedure for the Friedländer Synthesis to **9**:

1.0 equiv. Ketone **C** (25 mg, 0.072 mmol) was dissolved in EtOH (2.0 mL) together with 2-amino-5-bromobenzaldehyde (22 mg, 0.108 mmol) and three drops of $HCl_{(conc.)}$ in a 10 mL microwave pressure vessel. The resulting orange solution was stirred in the microwave at 130 °C for 3 h. After consumption of the starting material, 5.0 mL of water was added, and extraction was performed with EtOAc (10 mL × 3), followed by washing with brine (2.0 mL) and subsequent drying with Na_2SO_4. Concentration *in vacuo* resulted in a yellow crude oil, which was purified by preparative reversed-phase HPLC (C18, MeCN/H_2O gradient, 0.1% TFA), yielding **9** (26 mg, 70%) as an amorphous yellow solid.

Data for **9**

^{1}H NMR (700 MHz, $CDCl_3$) δ 8.31 – 8.11 (m, 2H), 8.08 (s, 1H), 7.91 (d, *J* = 9.0 Hz, 1H), 6.90 (s, 1H), 6.88 – 6.75 (m, 2H), 4.79 – 4.61 (m, 1H), 4.35 – 4.08 (m, 2H), 3.92 – 3.75 (m, 7H), 3.62 (d, *J* = 15.6 Hz, 1H), 3.59 – 3.49 (m, 1H), 3.45 – 3.41 (m, 1H), 3.25 – 3.02 (m, 1H), 2.33 – 2.27 (m, 2H), 2.10 – 2.03 (m, 1H), 1.25 (s, 3H). **^{13}C NMR (176 MHz, $CDCl_3$) δ** 159.4, 149.5, 148.3, 139.4, 138.4, 137.6, 135.5, 132.2, 129.7, 128.9, 125.6, 122.7, 117.6, 113.1, 111.2, 109.4, 61.4, 56.1, 56.0, 50.0, 45.3, 40.8, 39.1, 32.9, 29.4, 14.8. **HRMS (ESI)** $C_{26}H_{27}BrN_2O_4$ [M+H]+ calc. 511,1227 found 511,1215

Note: Partial overlap of 2.10 – 2.03 with residual HDO

Ethyl (3a,5,7a)-3a-(3,4-dimethoxyphenyl)-6'-methoxy-2,2',3,3a,3',4',6,7,7a,9'-decahydrospiro[indole-5,1'-pyrido[3,4-*b*]indole]-1(4*H*)-carboxylate (11)

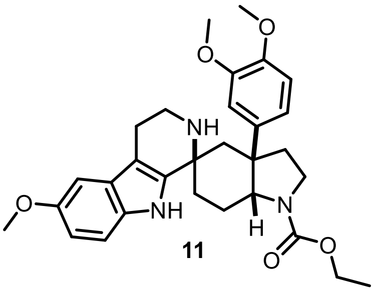

11

Procedure for the Pictet-Spengler Reaction to **11:**

1.0 equiv. Ketone **C** (25 mg, 0.072 mmol) was dissolved in Pyridine (2.0 mL) together with 1.2 equiv. of 5-Methoxy-Tryptamine (16 mg, 0.086 mmol) in a 10 mL microwave pressure vessel and TMSCl with 5 equiv. (46 μL, 0.360 mmol) was added dropwise. The yellow suspension was stirred in the microwave at 130 °C for 4 h. After letting the reaction cool down, 5.0 mL of water were aded and extraction was performed with EtOAc (10 mL × 3), washing with brine (2.0 mL), subsequent drying with Na_2SO_4, and concentration *in vacuo* resulted in a brown crude oil, which was purified by preparative reversed-phase HPLC (C18, MeCN/H_2O gradient, 0.1% TFA) yielding **7a** (26 mg, 70%) as a colorless oil.

Data for **11**

^{1}H NMR (700 MHz, $CDCl_3$) δ 11.34 – 9.05 (m, 1H), 7.58 – 7.31 (m, 1H), 7.00 – 6.65 (m, 6H), 4.72 – 4.38 (m, 1H), 4.34 – 4.02 (m, 2H), 3.89 (s, 3H), 3.84 – 3.74 (m, 6H), 3.73 – 3.62 (m, 1H), 3.53 – 3.30 (m, 3H), 3.20 – 3.05 (m, 1H), 2.95 (d, *J* = 15.9 Hz, 1H), 2.84 (d, *J* = 15.2 Hz, 1H), 2.53 – 2.22 (m, 5H), 2.16 – 2.05 (m, 2H), 1.31 – 1.20 (m, 3H). **^{13}C NMR (176 MHz, $CDCl_3$) δ** 155.3, 154.6, 149.8, 148.5, 138.0, 133.4, 131.1, 126.2, 119.1, 117.8, 117.5, 115.8, 114.1, 111.9, 111.4, 109.7, 106.8, 100.5, 61.4, 57.9, 56.2, 56.1, 56.0, 47.2, 43.6, 38.9, 30.4, 22.9 18.8, 14.8. **HRMS (ESI)** [M+H]+ $C_{30}H_{37}N_3O_5$ calc. 520.2806 found 520.2804

Structure Determination by NMR Analysis

Figure S2. Structure elucidation of Linear Indole **2** using COSY NMR

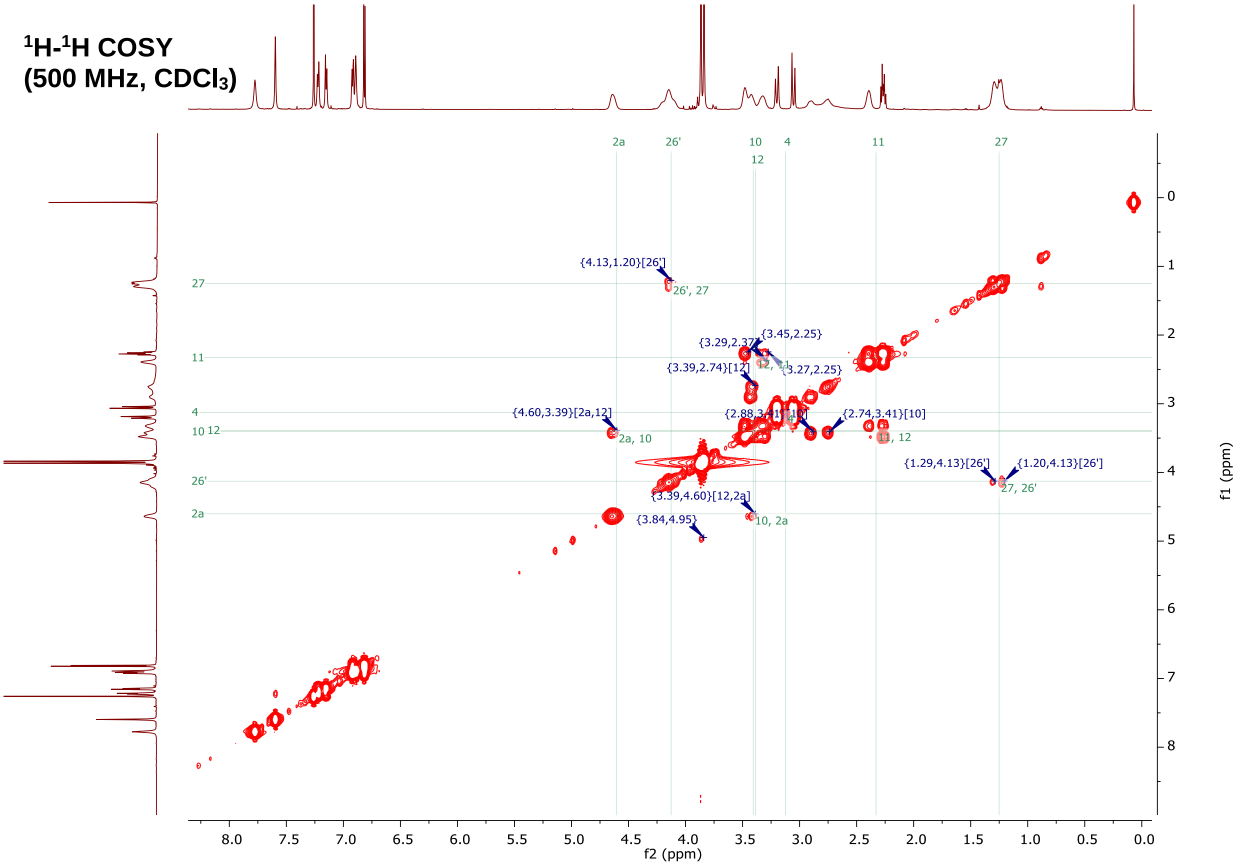

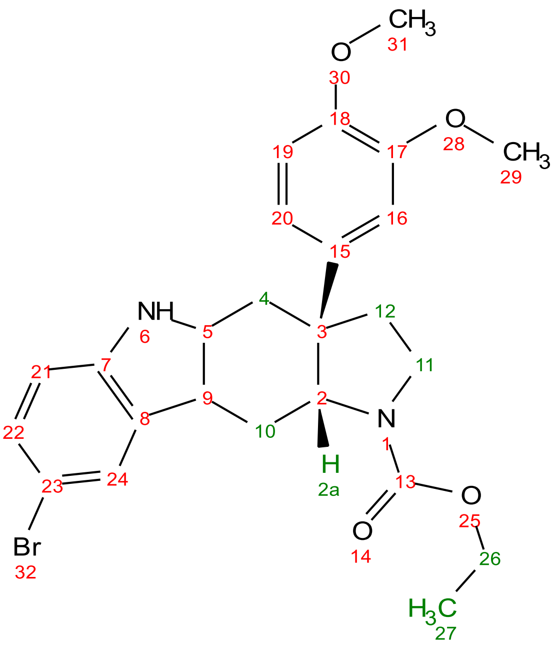

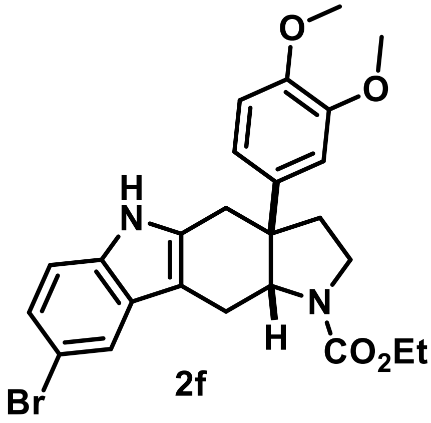

2f

Figure S3. Structure elucidation of Linear Indole **2** using COSY NMR (Zoom)

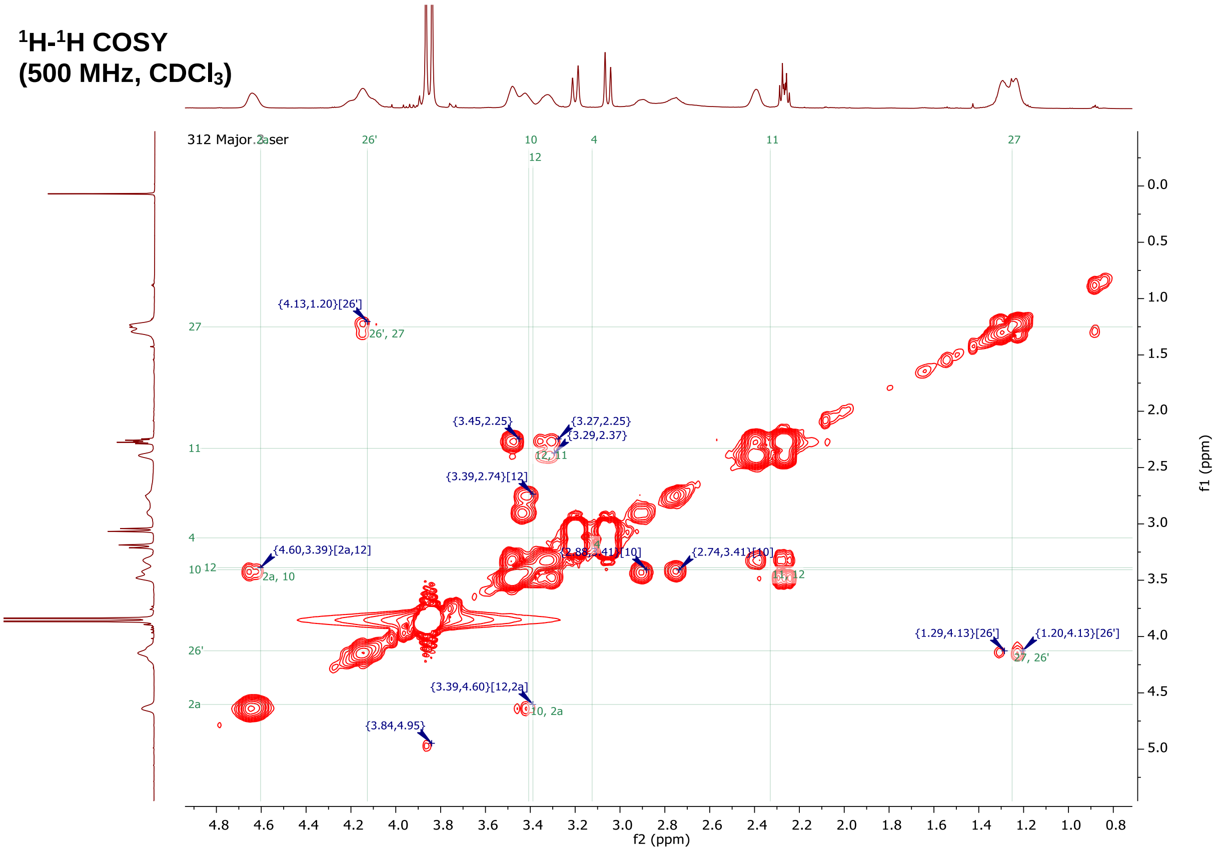

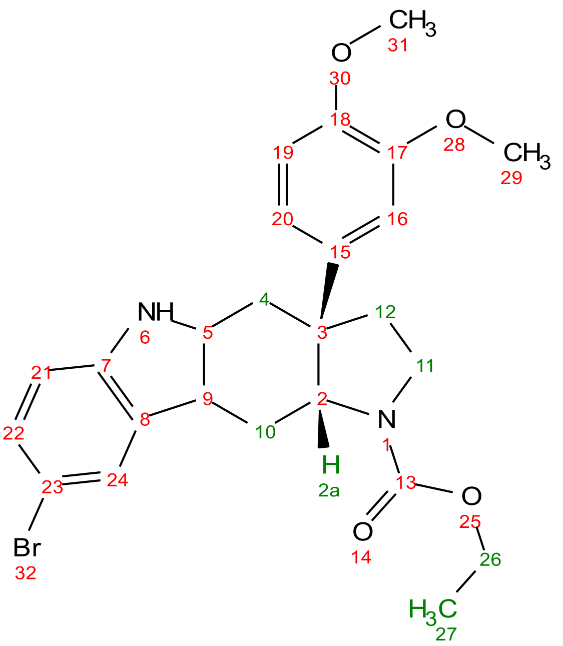

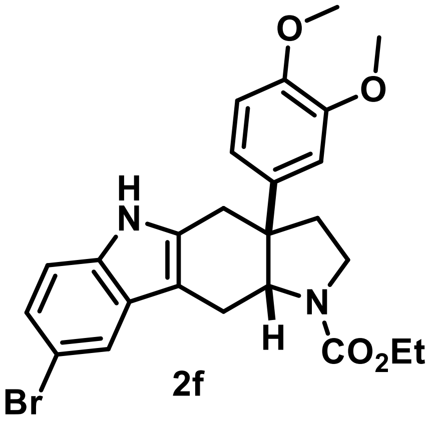

2f

Figure S4. Structure elucidation of Linear Indole **2** using NOESY NMR

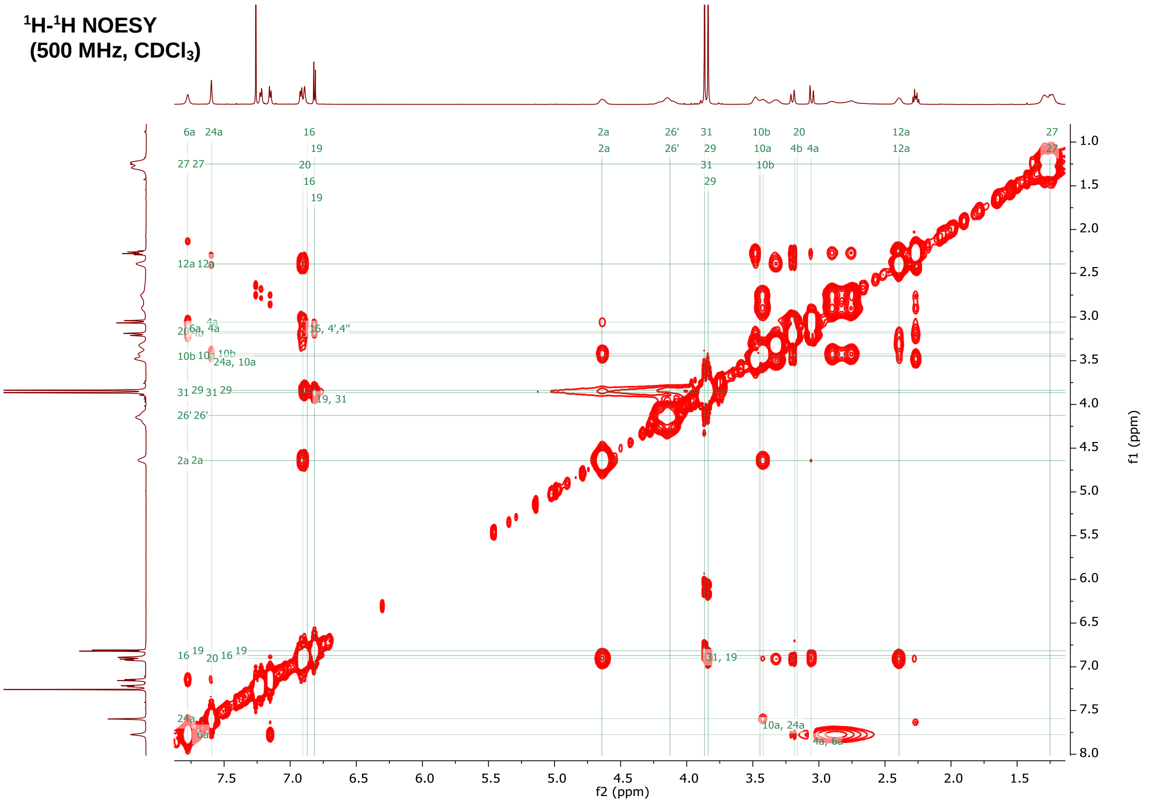

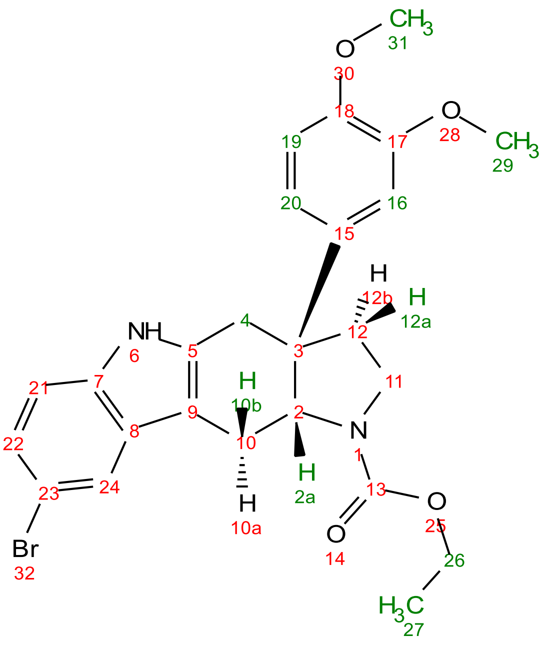

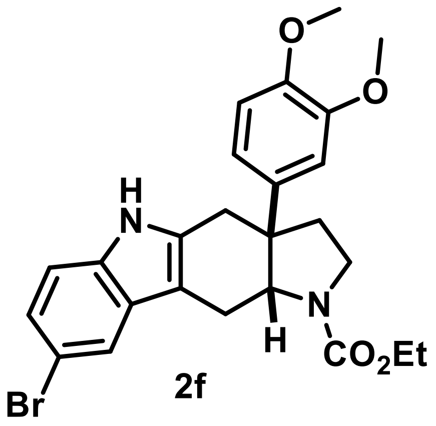

2f

Figure S5. Structure elucidation of Linear Indole **2** using NOESY NMR (zoom)

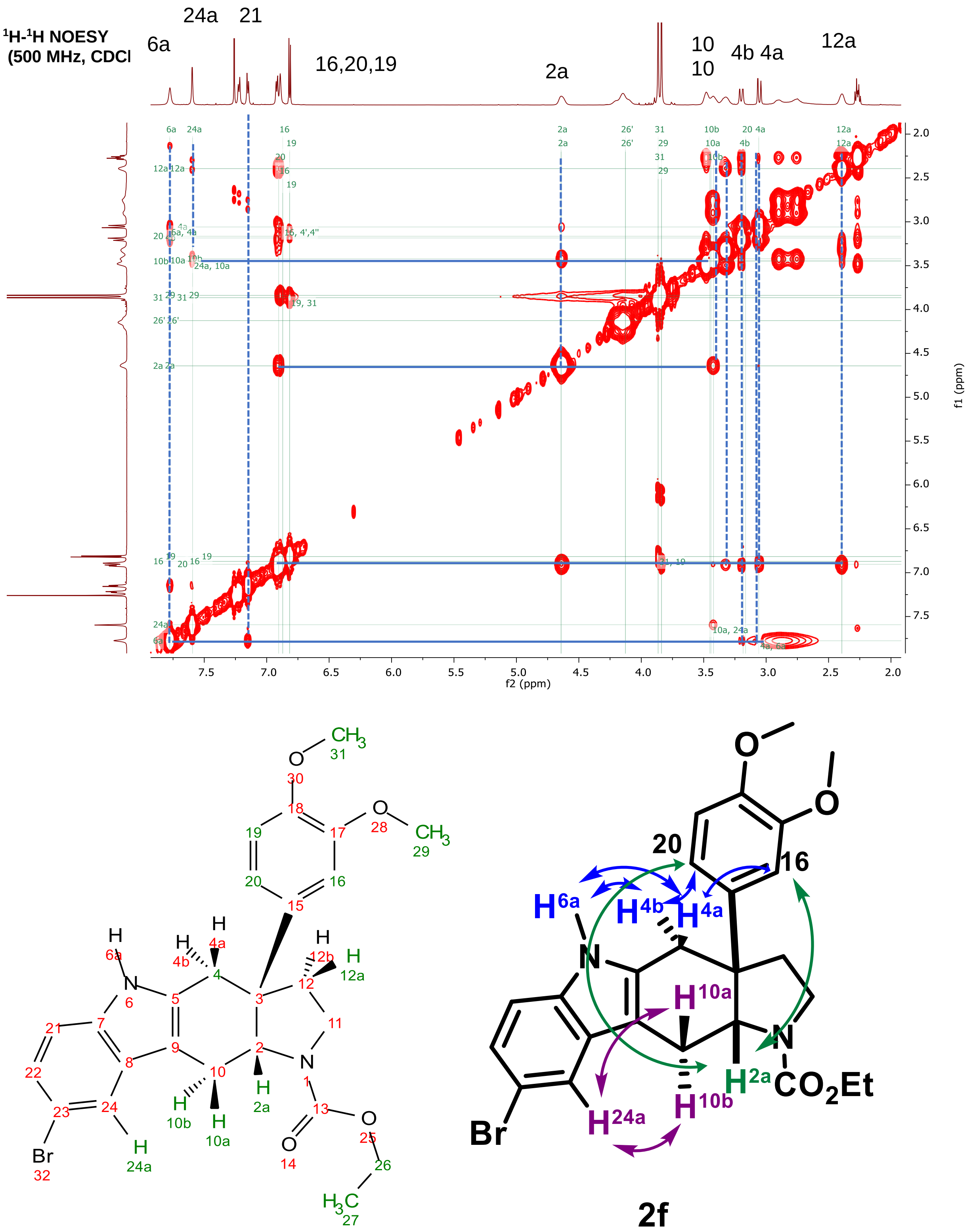

Figure S6. Structure elucidation of fused pyridine **7** using COSY NMR

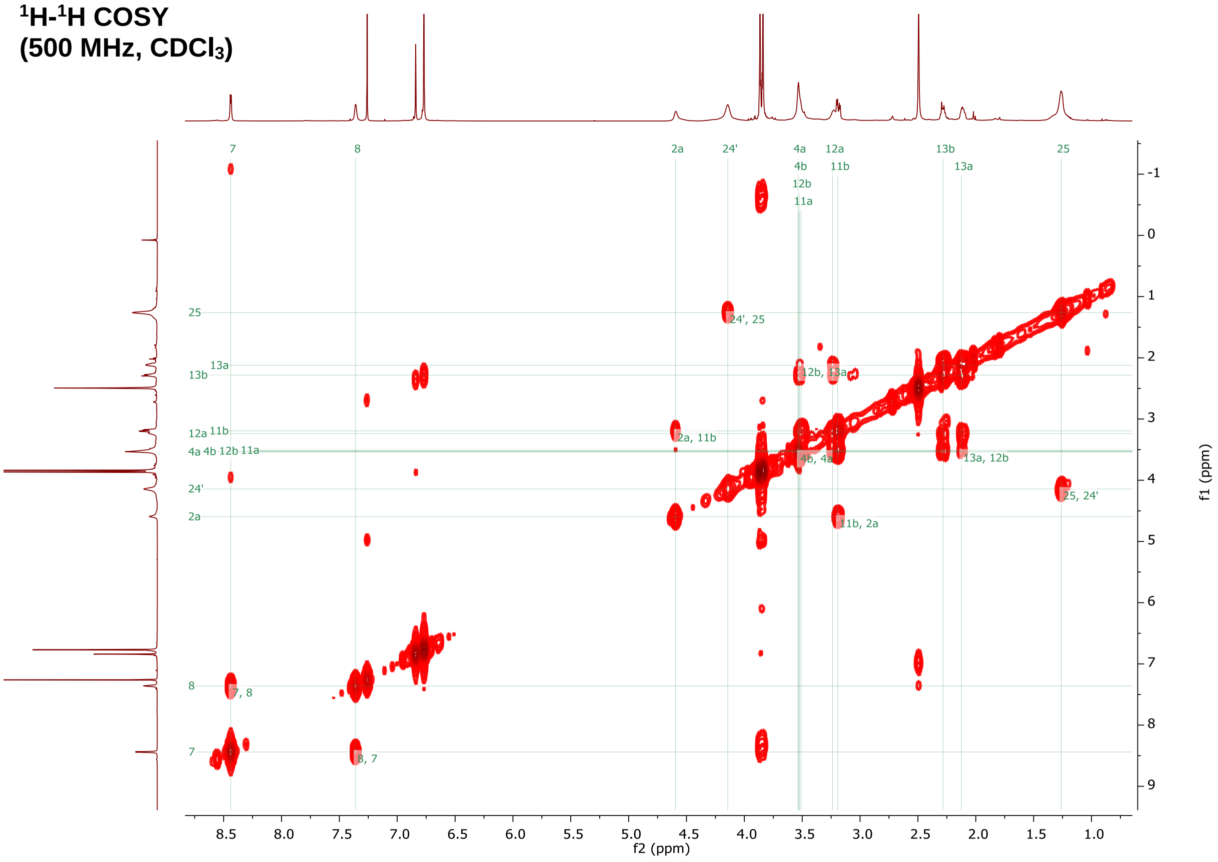

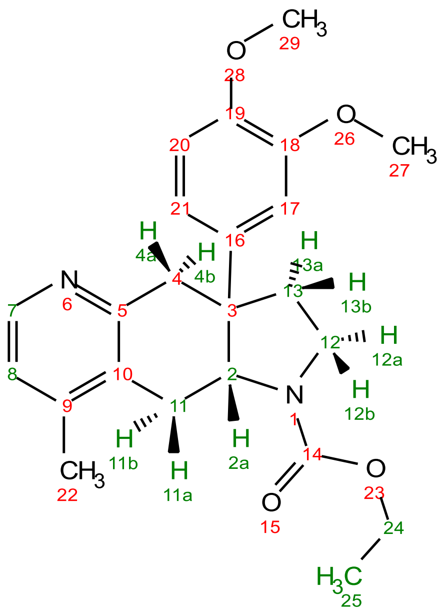

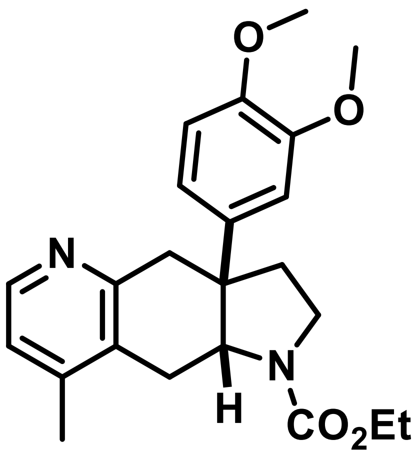

7b

Figure S7. Structure elucidation of fused pyridine **7** using COSY NMR (zoom)

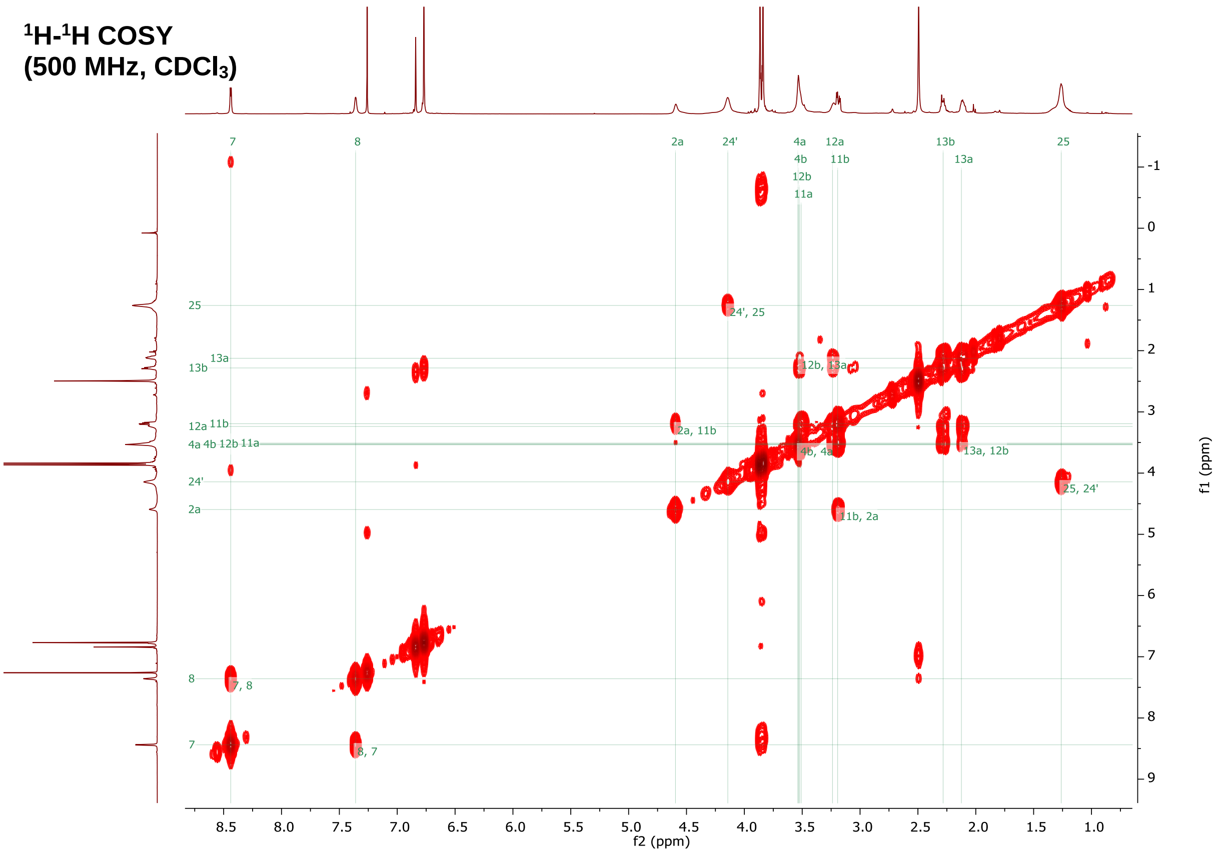

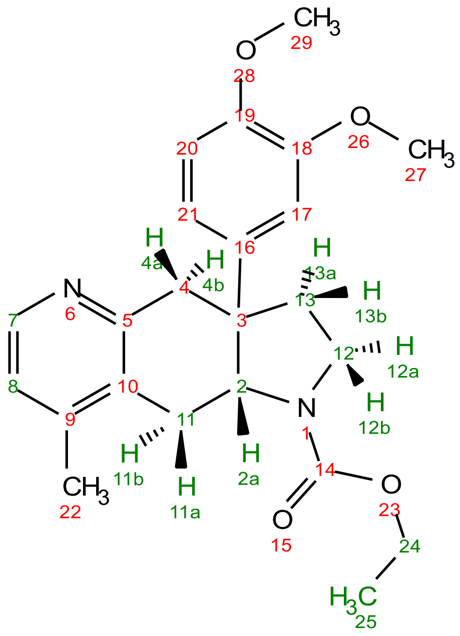

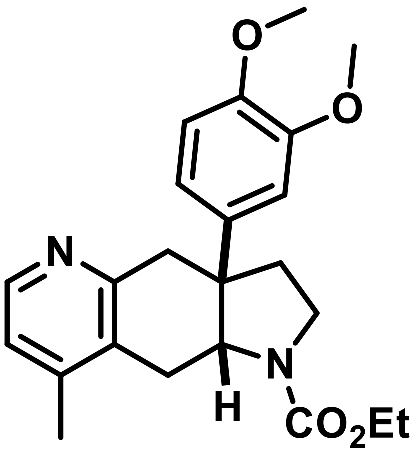

7b

Figure S8. Structure elucidation of fused pyridine **7** using NOESY NMR

^{1}H-^{1}H NOESY
(500 MHz, $CDCl_3$)

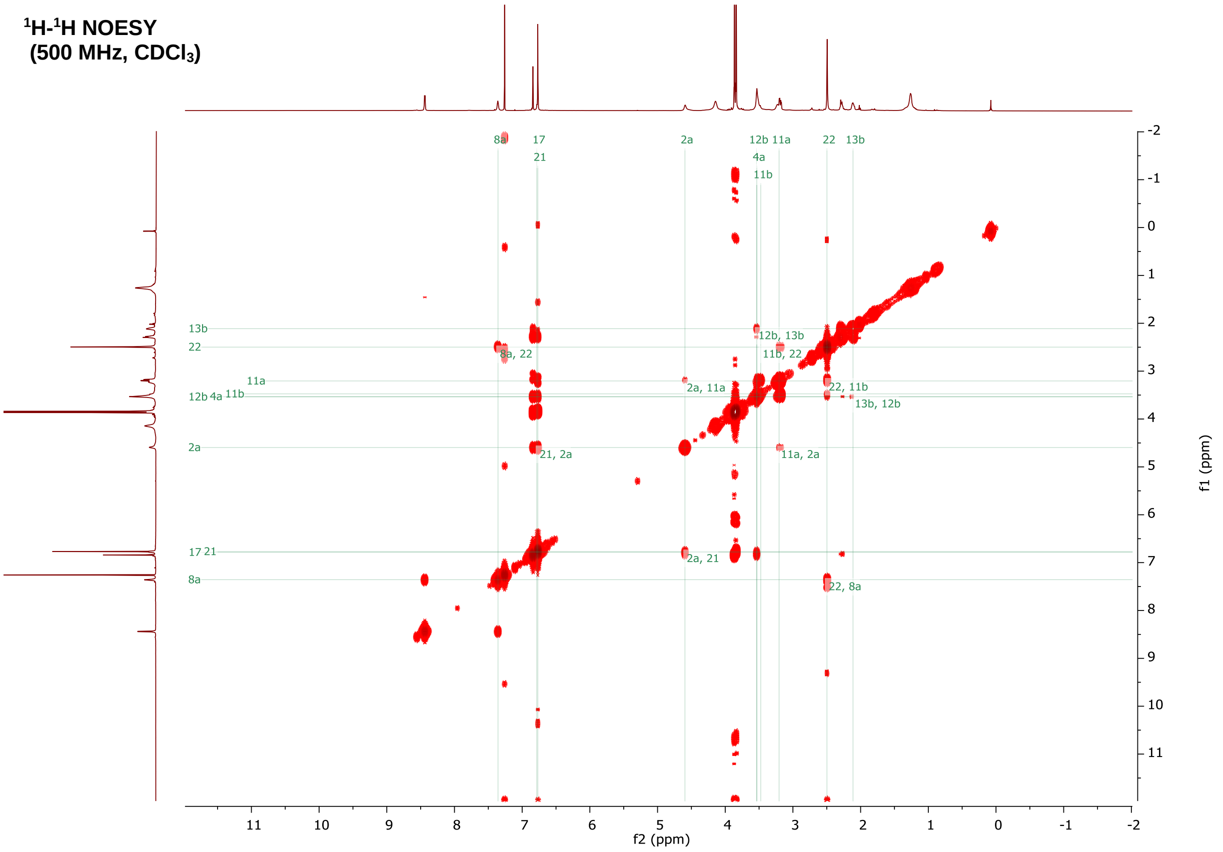

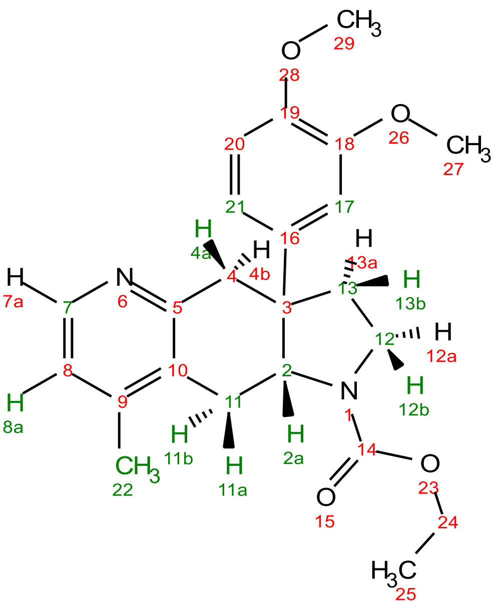

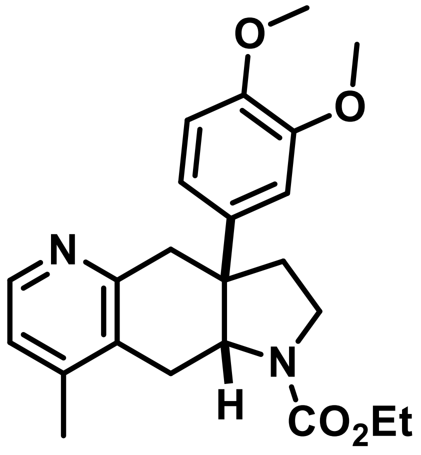

7b

Figure S9. Structure elucidation of fused pyridine **7** using NOESY NMR (zoom)

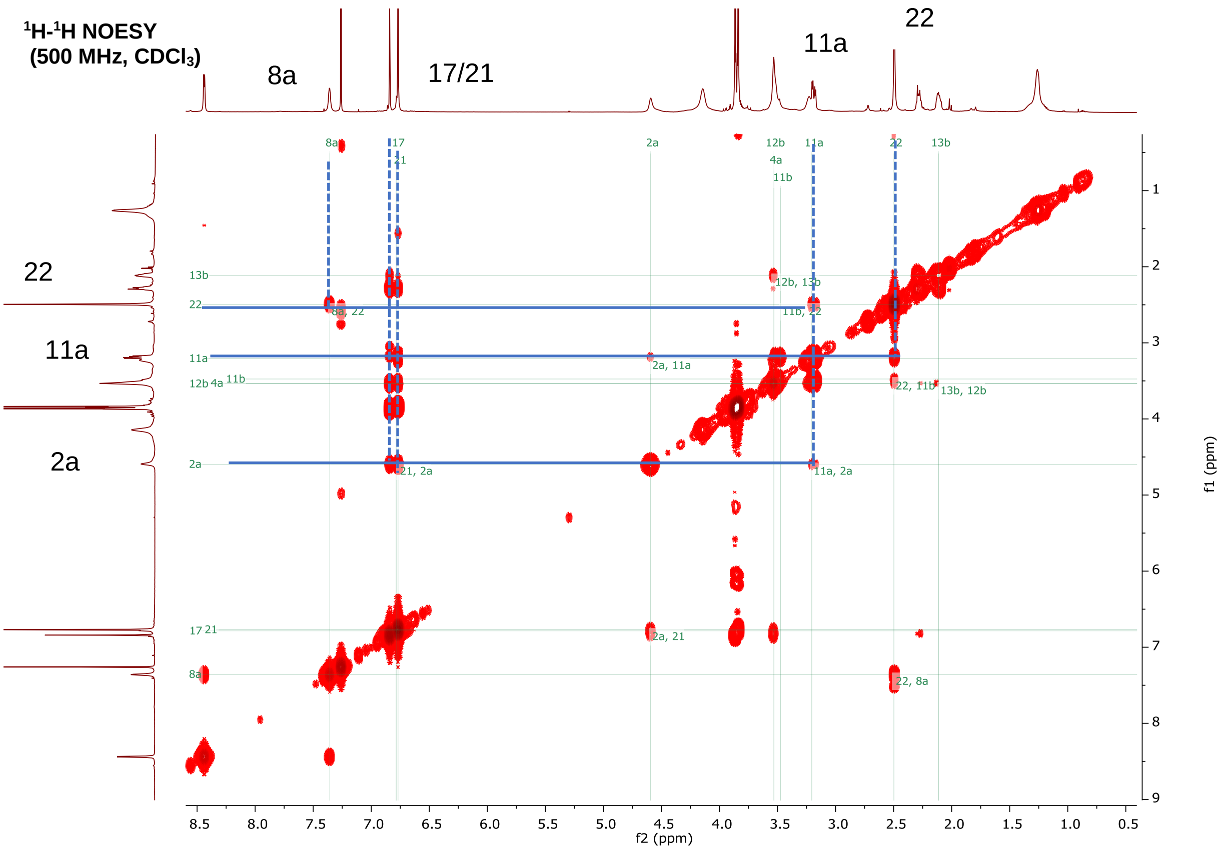

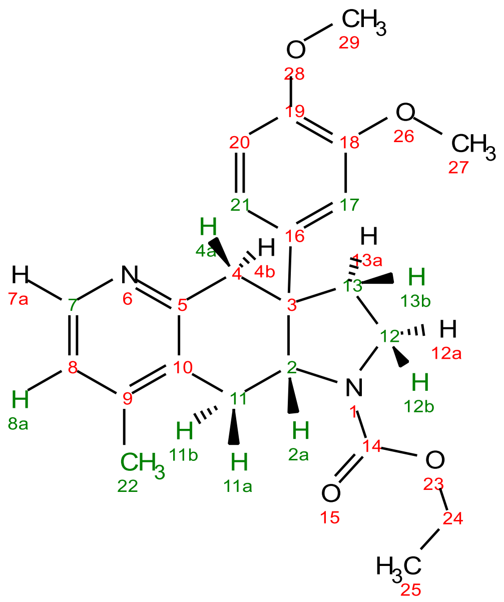

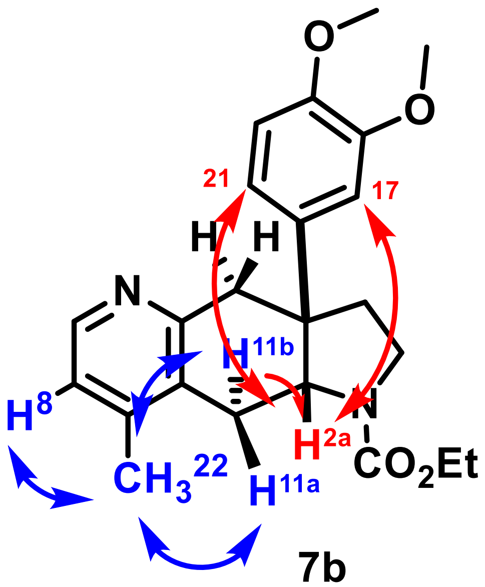

7b

Figure S10. Structure elucidation of angular indole **3** using COSY NMR

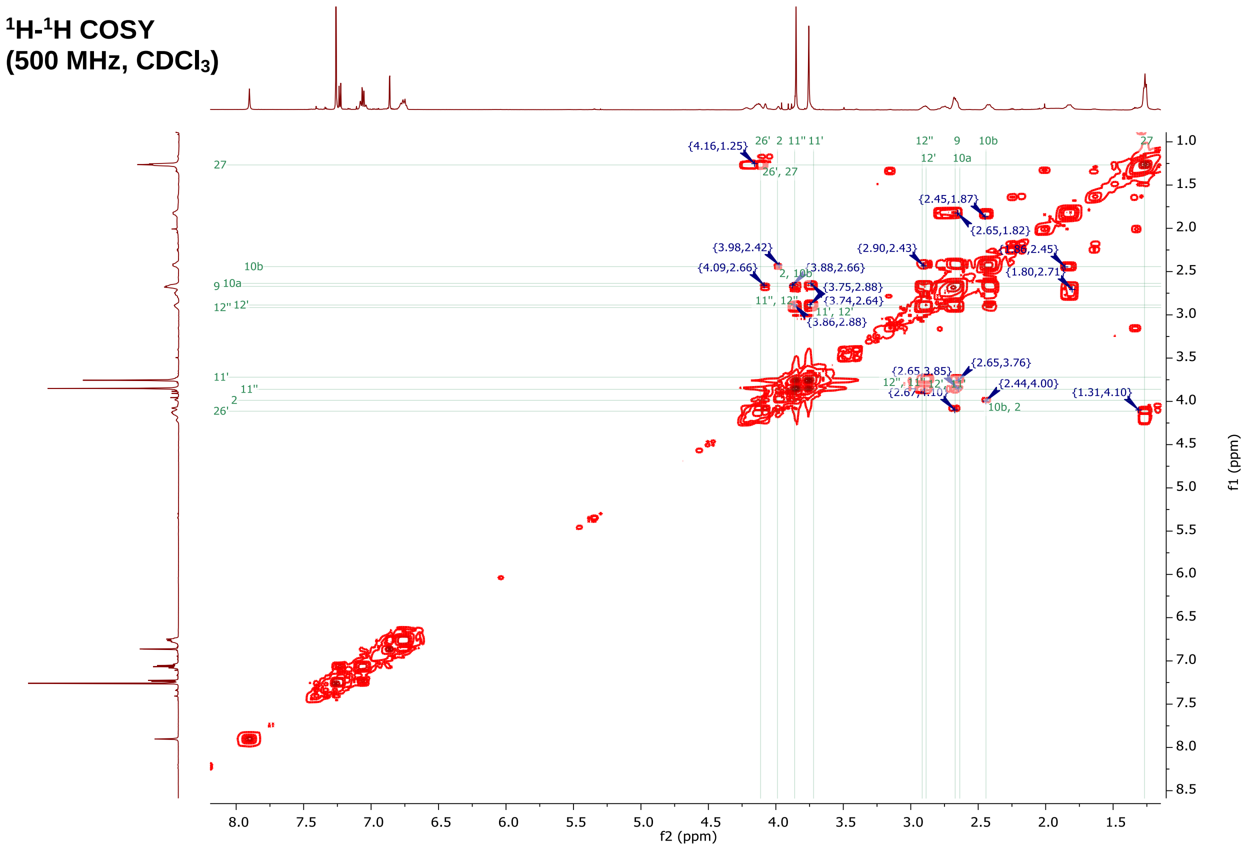

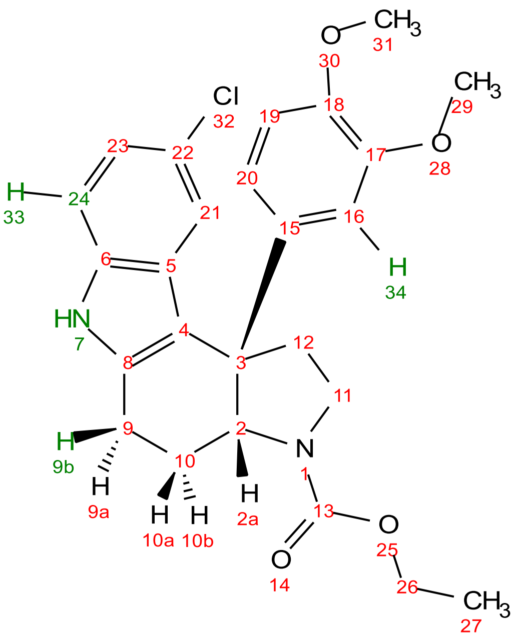

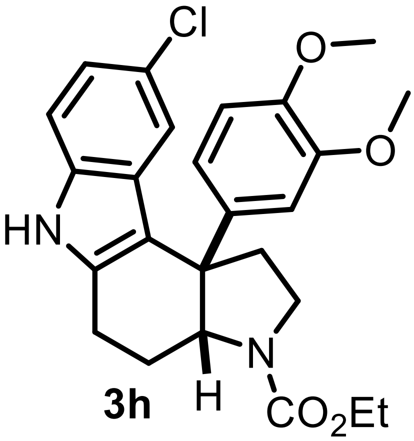

3h

Figure S11. Structure elucidation of angular indole **3** using COSY NMR (zoom)

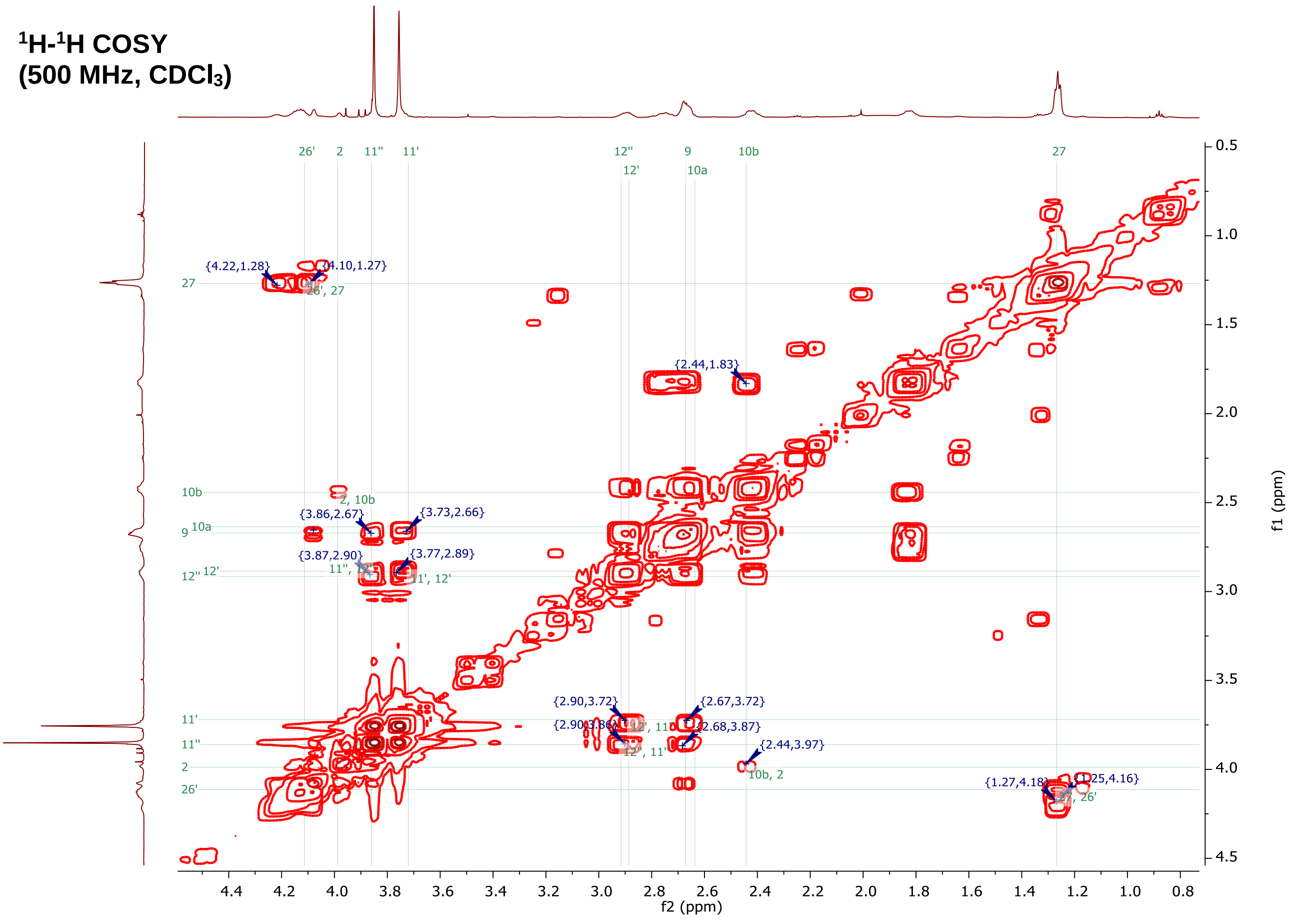

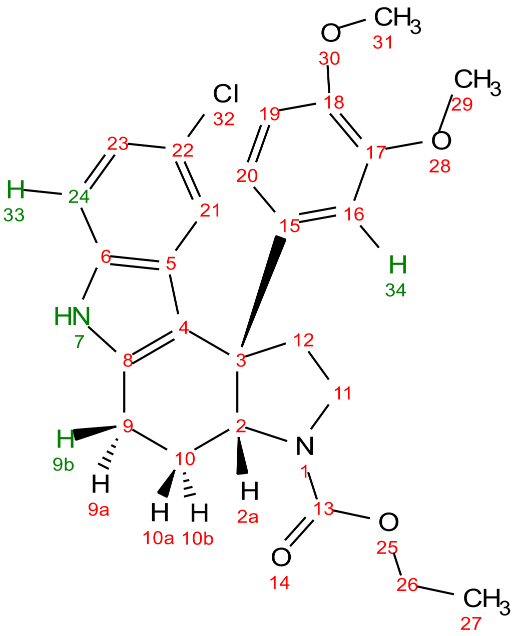

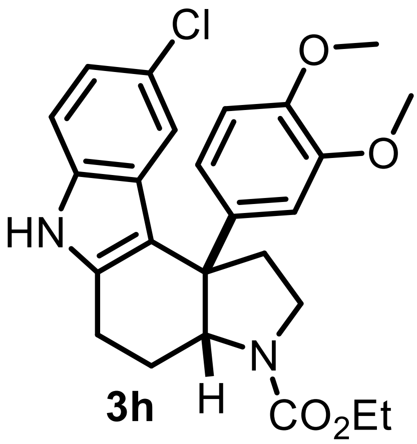

Figure S12. Structure elucidation of angular indole **3** using NOESY NMR

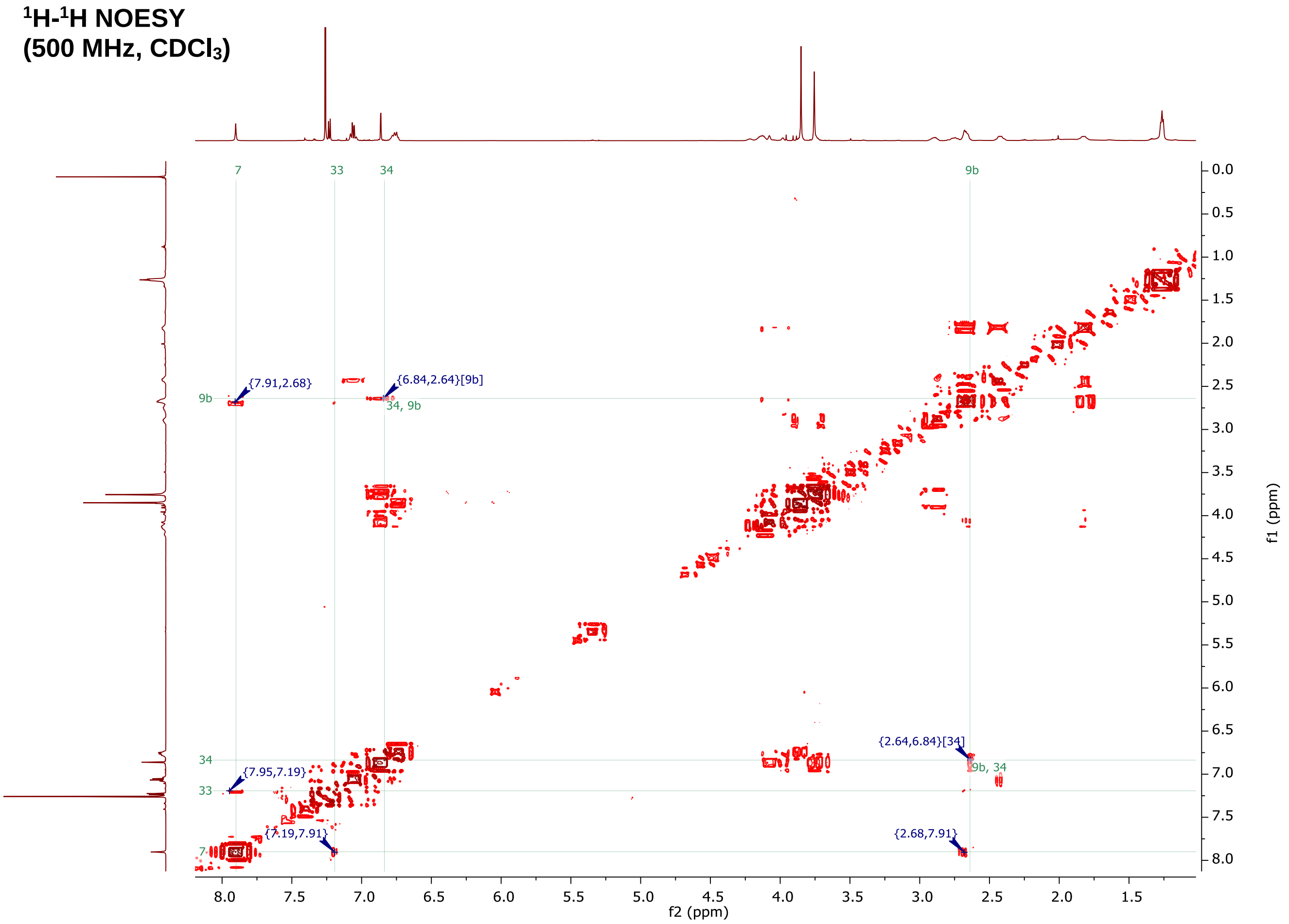

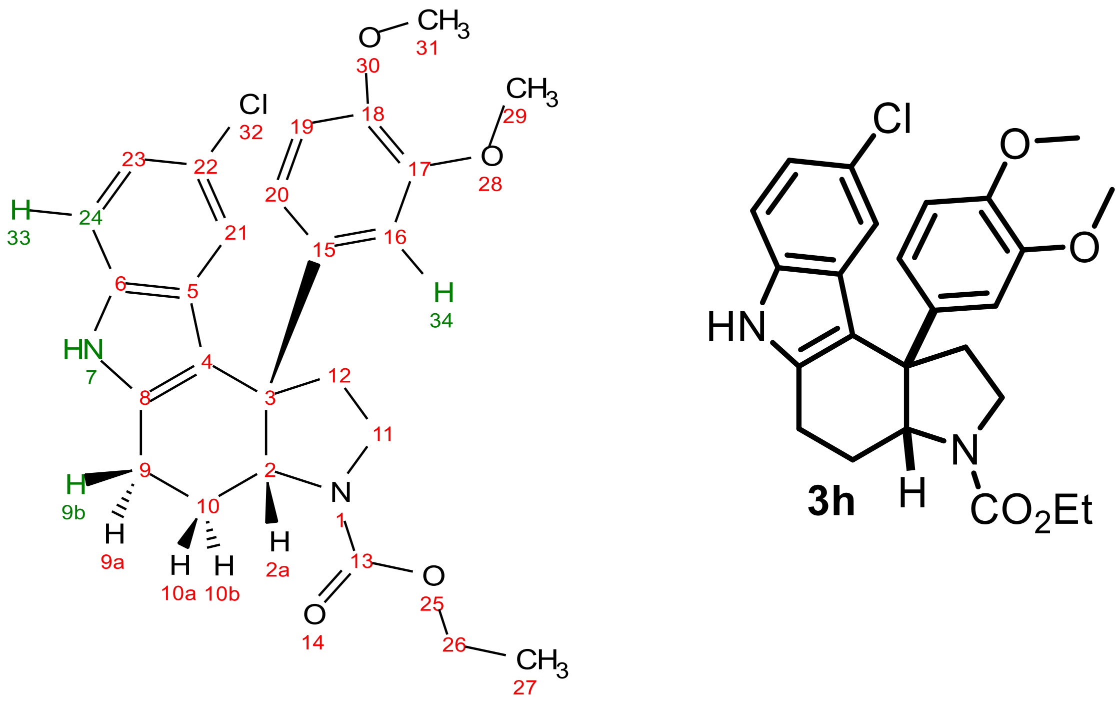

Figure S13. Structure elucidation of angular indole **3** using NOESY NMR (zoom)

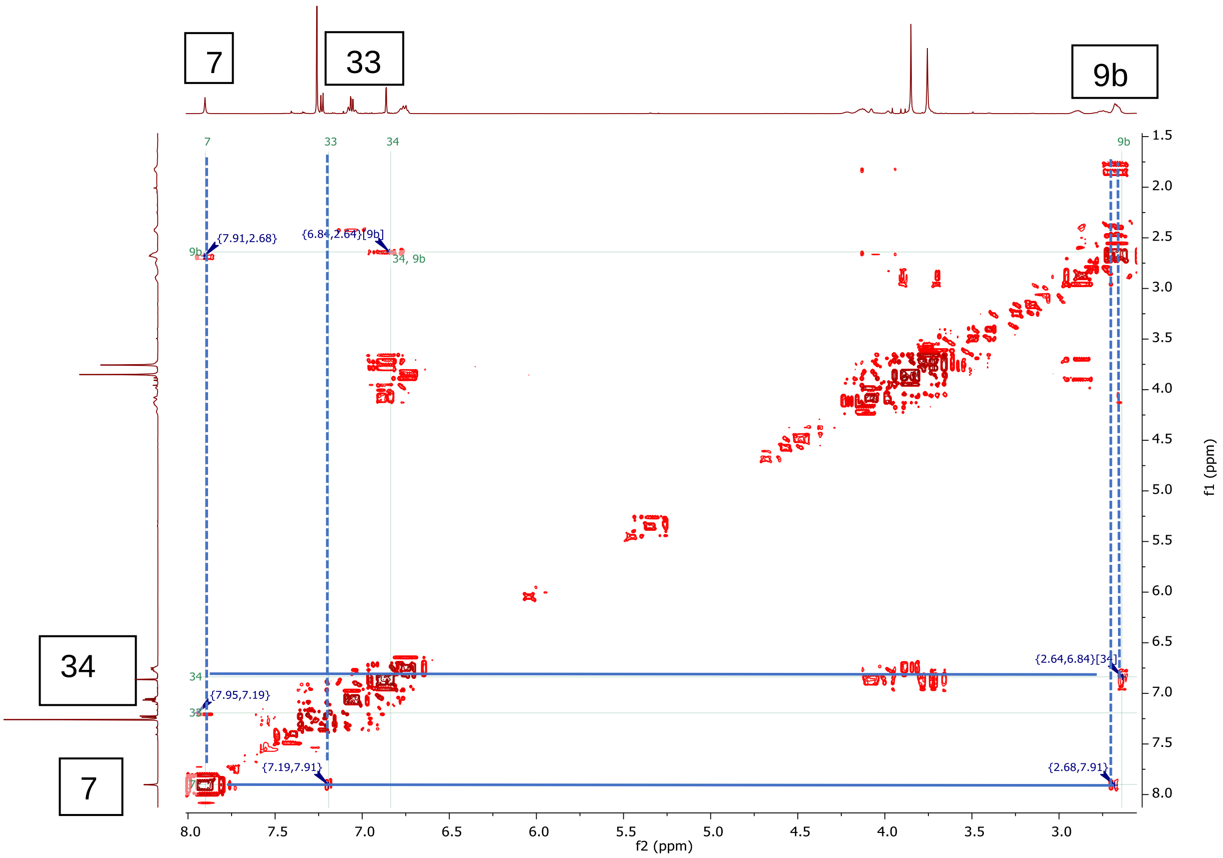

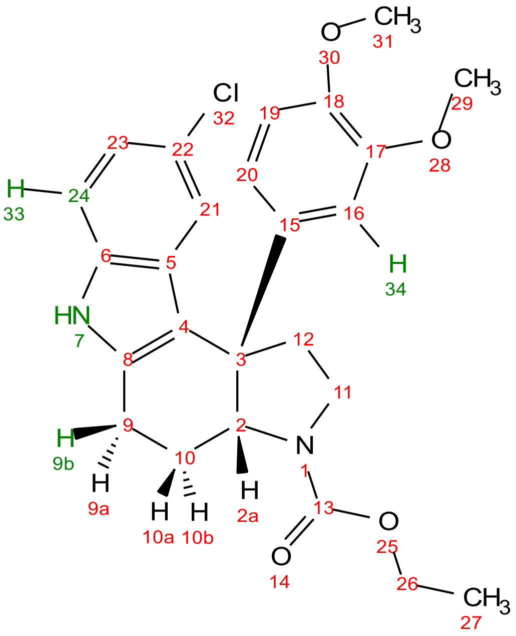 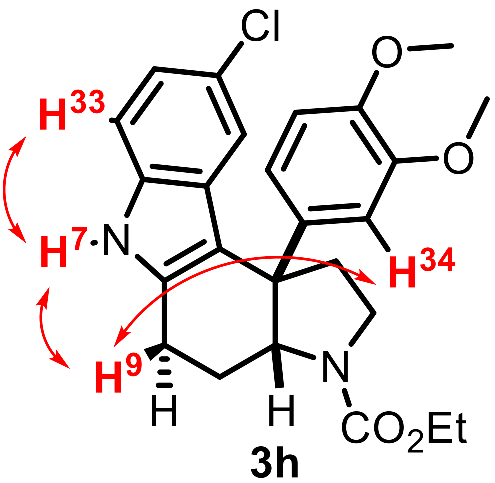

3h

General Chemoinformatic Data

Cheminformatics analyses

Chemical space comparisons were carried out using different molecular features in Python as described below. Compounds having more than 75 heavy atoms were also excluded from analysis.

1. Dataset preparation

DrugBank (version 5.1.13),[4] Enamine Advanced Screening Collection,[5] ChEMBL 35,[6] and COCONUT[7] were used to generate reference datasets. Each compound collection was directly obtained from their respective providers either as a SD file (DrugBank and ChEMBL) or as a database dump (SQLite for ChEMBL and PostgreSQL for COCONUT). A subsample of 50k compounds was randomly selected from Enamine's library for further use, whereas the ChEMBL library was filtered for

compounds flagged as natural products. The selected ChEMBL NPs were submitted to sugar removal using the KNIME Sugar Remover node (CDK; KNIME 5.5.0).[8] Representative *Sceletium*, Amaryllidaceae, and monoterpene indole alkaloids (MIAs) were filtered from ChEMBL by substructure search. Hasubanan alkaloids were retrieved from COCONUT (larger, more complete set).

For all compound libraries, structures were standardized. Compounds containing unusual isotopes and unusual atoms from a medicinal chemistry perspective were removed.

2. Molecular features

A set of 17 molecular descriptors (molecular weight, ring count, number of aromatic rings, number of aliphatic rings, number of Hydrogen bond donors, number of Hydrogen bond acceptors, LogP, topological polar surface area, number of rotatable bonds, halogen fraction, number of bridge atoms, sp^3 fraction, number of heavy atoms, Oxygen count, Nitrogen count, Lipinski's rules violations, and Veber's rules violations) were calculated and used to characterize each compound collection as previously done by Grigalunas et al.[9]. Data analysis was accomplished by means of Principal Component Analysis (PCA) as dimensionality reduction technique, using three principal components.

3. Calculation of drug-like scores

Four different scoring systems were used in this study: NP Likeness score, developed by Ertl et al.,[10] QED (Quantitative Estimate of Druglikeness, developed by Bickerton et al.,[11] Böttcher score, defined by Böttcher,[12] and implemented by Demoret et al. ,[13] and nSPS normalized Spacial Score developed by Krzyzanowski et al..[14]

4. Principal Moments of Inertia

Characterization of the compound collections by molecular shape was accomplished using the Principal Moments of Inertia (PMI) introduced by Sauer and Schwarz.[15] In order to calculate PMIs, compounds were transformed into 3D structures using the RDKit[16] (https://www.rdkit.org) and minimized with the Universal Force Field.[17] A typical shape plot using the first and second normalized PMIs was used for visual analysis.

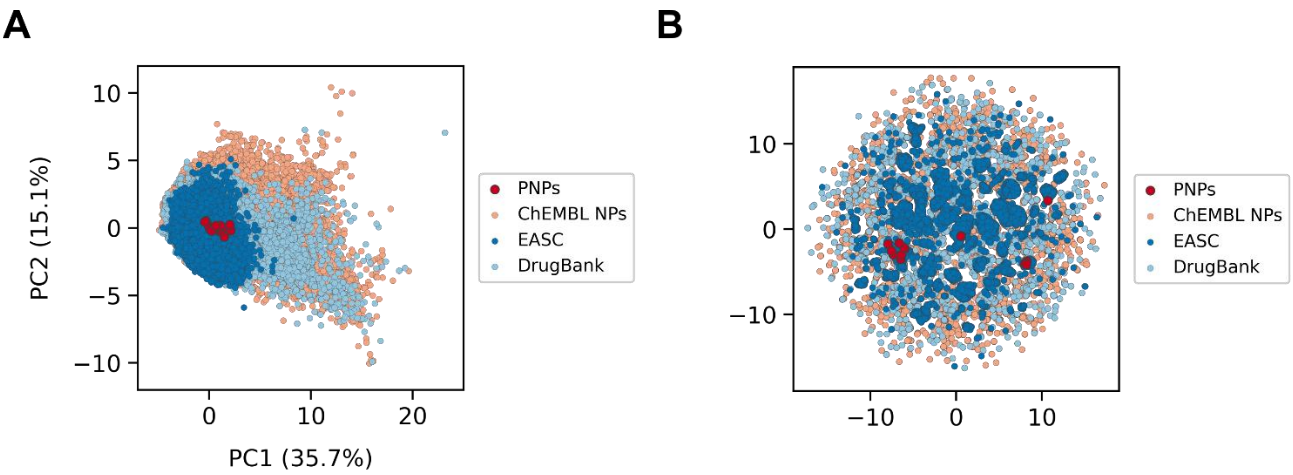

Figure S14. A) Principal Component Analysis (PCA) on 17 molecular descriptors. **B)** Uniform Manifold Approximation and Projection (UMAP) on 17 molecular descriptors

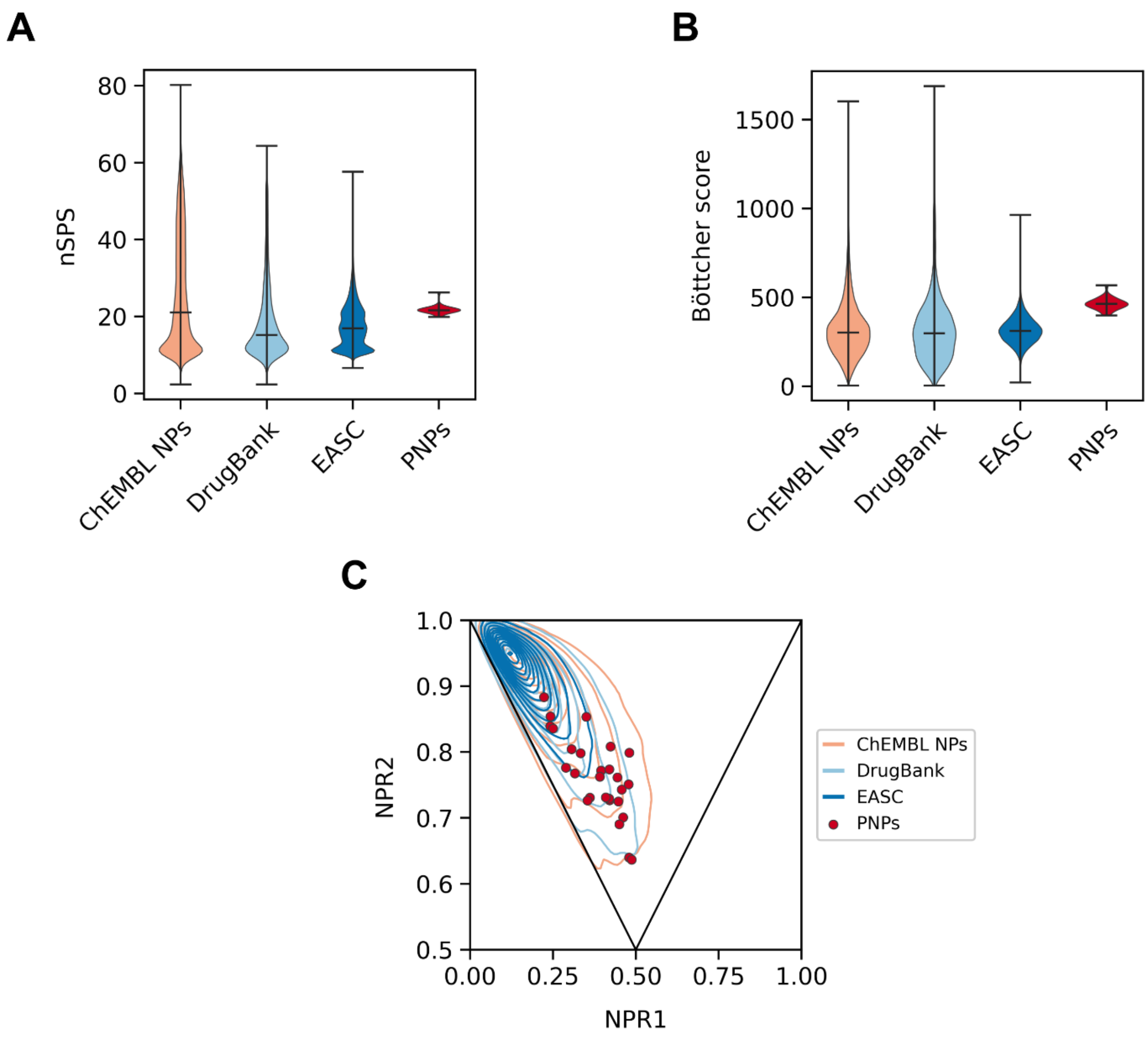

Figure S15. A) normalized Spatial score. **B)** Böttcher-Score. **C)** normalized Principal Moments of Inertia (PMIs). Comparisons are based on 78k natural products registered in ChEMBL, 11k compounds in the DrugBank, and a random sample of 50k compounds from the Enamine Advanced Screening Collection.

Biological Experimental Details

General Biological Experimental Details

**Commercial Kits and reagents**	**Supplier**	**Product Number**
Seahorse XF Calibrate	Agilent	100840-000
Seahorse XF DMEM medium pH 7.4	Agilent	103575-100
Seahorse XFp Mito Stress Test Kit	Agilent	103010-100
**Cell lines**	**Supplier**	
Human U-2OS cells (female)	CLS	Cat# 300364; RRID:CVCL_0042
**Software**		
Seahorse Wave	Agilent	
**Devices**		
Seahorse XFp analyzer	Agilent	

Cell culture

Osteosarcoma U-2OS cells (#300364, RRID: CVCL_0042) were obtained from CLS and were maintained in DMEM Dulbecco's Modified Eagle's medium (DMEM with 4.5 g/L glucose, L-glutamine and 3.7 g/L sodium bicarbonate; PAN Biotech, #P04-03550) supplemented with 10% fetal bovine serum (FBS, Invitrogen, cat# 10500-084), 1 mM sodium pyruvate (PAN, #P04-43100) and 1% MEM-non-essential amino acids (PAN, #P08-32100).

Cell Painting Assay Details

The described assay follows closely the method described by Bray et al.[18] and as recently reported.[19] "Initially, 5 µl U2OS medium were added to each well of a 384-well plate (PerkinElmer CellCarrier-384 Ultra). Subsequently, U2OS cell were seeded with a density of 1600 cells per well in 20 µl medium. The plate was incubated for 10 min at the ambient temperature, followed by an additional 4 h incubation (37 °C, 5% CO_2). Compound treatment was performed with the Echo 520 acoustic dispenser (Labcyte) at final concentrations of 10 µM, 3 µM or 1 µM. Incubation with compound was performed for 20 h (37 °C, 5% CO_2). Subsequently, mitochondria were stained with Mito Tracker Deep Red (Thermo Fisher Scientific, Cat. No. M22426). The Mito Tracker Deep Red stock solution (1 mM) was diluted to a final concentration of 100 nM in prewarmed medium. The medium was removed from the plate leaving 10 µl residual volume and 25 µl of the Mito Tracker solution were added to each well. The plate was incubated for 30 min in darkness (37 °C, 5% CO_2). To fix the cells 7 µl of 18.5 % formaldehyde in PBS were added, resulting in a final formaldehyde concentration of 3.7 %. Subsequently, the plate was incubated for another 20 min in darkness (RT) and washed three times with 70 µl of PBS. (Biotek Washer Elx405). Cells were permeabilized by addition of 25 µl 0.1% Triton X-100 to each well, followed by 15 min incubation (RT) in darkness. The cells were washed three times with PBS leaving a final volume of 10 µl. To each well 25 µl of a staining solution were added, which contains 1% BSA, 5 µl/ml Phalloidin (Alexa594 conjugate, Thermo Fisher Scientific, A12381), 25 µg/ml Concanavalin A (Alexa488 conjugate, Thermo Fisher Scientific, Cat. No. C11252), 5 µg/ml Hoechst 33342 (Sigma, Cat. No. B2261-25mg), 1.5 µg/ml WGA-Alexa594 conjugate (Thermo Fisher Scientific, Cat. No. W11262) and 1.5 µM SYTO 14 solution (Thermo Fisher Scientific, Cat. No. S7576). The plate is incubated for 30 min (RT) in darkness and washed three times with 70 µl PBS. After the final

washing step, the PBS was not aspirated. The plates were sealed and centrifuged for 1 min at 500 rpm. The plates were prepared in triplicates with shifted layouts to reduce plate effects and imaged using a Micro XL High-Content Screening System (Molecular Devices) in 5 channels (DAPI: Ex350-400/ Em410-480; FITC: Ex470-500/ Em510-540; Spectrum Gold: Ex520-545/ Em560-585; TxRed: Ex535-585/ Em600-650; Cy5: Ex605-650/ Em670-715) with 9 sites per well and 20x magnification (binning 2).

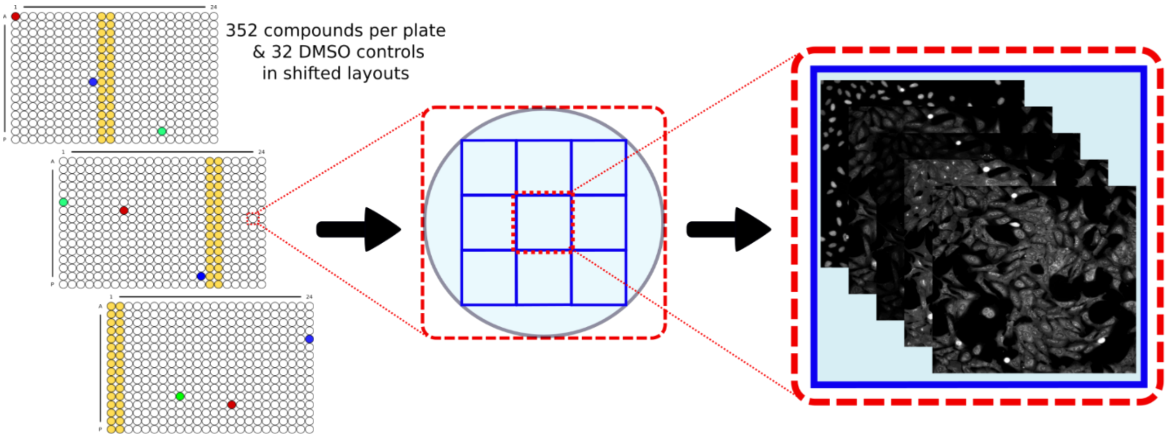

The generated images were processed with the *CellProfiler* package (https://cellprofiler.org/, version 3.0.0) on a computing cluster of the Max Planck Society to extract 1716 cell features per microscope site.[20] The data was then further aggregated as medians per well (9 sites -> 1 well), then over the three replicates.

Further analysis was performed with custom *Python* (https://www.python.org/) scripts using the *Pandas* (https://pandas.pydata.org/) and *Dask* (https://dask.org/) data processing libraries as well as the *Scientific Python* (https://scipy.org/) package (separate publication to follow). From the total set of 1716 features, a subset of highly reproducible and robust features was determined using the procedure described by Woehrmann et al.[21] in the following way: Two biological repeats of one plate containing reference compounds were analysed. For every feature, its full profile over each whole plate was calculated. If the profiles from the two repeats showed a similarity >= 0.8 (see below), the feature was added to the set. This procedure was only

performed once and resulted in a set of 579 robust features out of the total of 1716 that was used for all further analyses.

Determination of reproducible Features

1716 — *Determined by CellProfiler*

↓ *Keep features that have a minimum correlation of 0.80 between repeats for all cpds.*

579 — *Final set of relevant features. Used for all further analyses*

The phenotypic profiles were compiled from the Z-scores of all individual cellular features, where the Z-score is a measure of how far away a data point is from a median value. Specifically, Z-scores of test compounds were calculated relative to the Median of DMSO controls. Thus, the Z-score of a test compound defines how many MADs (Median Absolute Deviations) the measured value is away from the Median of the controls as illustrated by the following formula:

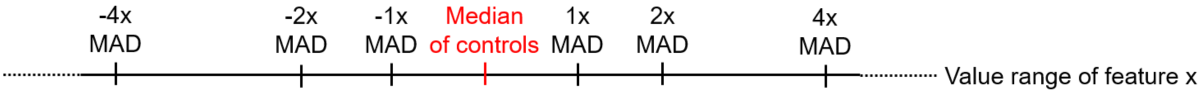

$$z - score = \frac{value_{meas.} - Median_{Controls}}{MAD_{Controls}}$$

The phenotypic compound profile is then determined as the list of Z-scores of all features for one compound. In addition to the phenotypic profile, an induction value was determined for each compound as the fraction of significantly changed features, in percent:

$$Induction\ [\%] = \frac{number\ of\ features\ with\ abs.\ \ values\ >\ 3}{total\ number\ of\ features}$$

Similarities of phenotypic profiles (termed *Biosimilarity*) were calculated from the correlation distances (CD) between two profiles (https://docs.scipy.org/doc/scipy/reference/generated/scipy.spatial.distance.correlation.html):

$$CD = 1 - \frac{(u - \bar{u}) \cdot (v - \bar{v})}{\|(u - \bar{u})\|_2 \|(v - \bar{v})\|_2}$$

where $\bar{x}$ is the mean of the elements of x, $x \cdot y$ is the dot product of x and y, and $\|x\|_2$is the Euclidean norm of x:

$$\|x\|_2 = \sqrt{x_1^2 + x_2^2 + \cdots + x_n^2}$$

The Biosimilarity is then defined as:

$$Biosimilarity = 1 - CD$$

Biosimilarity values smaller than 0 are set to 0 and the Biosimilarity is expressed in percent (0-100).

An example for two compounds with highly similar profiles (96% Biosimilarity):

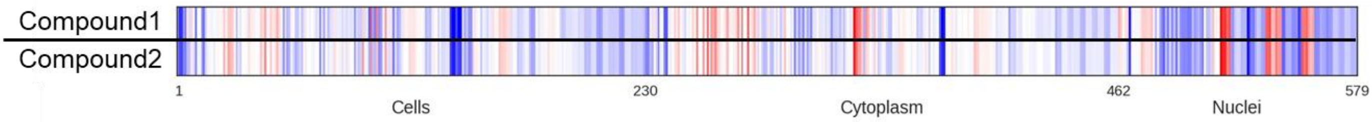

An example for two compounds with low similarity profiles (0% Biosimilarity):

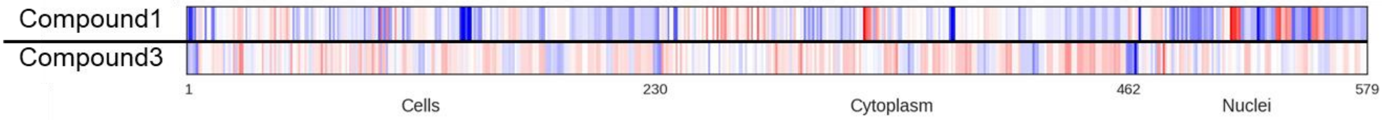

Each colored band represents one Z-score of a feature.”

Subprofile analysis

The subprofile analysis was followed exactly the same as reported by Wang et al.[22]; "Cluster subprofiles were generated as recently described by Pahl et al.[19] For each profile in a set of cluster-defining profiles, the sign for each of the 579 feature values was assessed and the counter for positive or negative values was determined. For all cluster-defining compounds, the maximum of the two counters was determined and divided by the total number of defining profiles. A given feature was added to the cluster profile if its value has the same sign (i.e., positive or negative feature values) for 85% of the defining profiles. Afterwards, a representative median subprofile for the cluster was calculated by taking the median values over all cluster-defining profiles for every given feature and combining them into a new reduced profile. This median (*consensus*) subprofile is then used to calculate the biosimilarity of profiles to the defined cluster subprofiles. As cluster subprofiles are shorter than the full profiles, the cluster biosimilarity threshold was set to 80%."

Seahorse XF Mito Stress Test

The Seahorse XF Mito Stress Test was performed using the Seahorse XFp Analyzer (Agilent, USA) according to the manufacturer's instructions after acute injection of the compound or after pre-incubation of cells with the compounds for 20 h.

MitoStress Test after Acute Injection

$4x10^4$ U-2OS cells were seeded per well into XFp cell culture mini plate prior to incubation overnight at 37°C, 5°% CO_2. The XFp cartridges were hydrated using the XF Calibrant and incubated overnight at 37°C. The cell medium was then replaced with pH 7.4 DMEM-based assay medium (Agilent, USA) containing 2°mM GlutaMAX (ThermoFisher), 1 mM sodium pyruvate (PAN Biotech, Germany) and 25°mM glucose (SigmaAldrich, Germany) and cells were incubated at 37°C without CO_2 for 45 min.

Five baseline recordings were acquired prior to injection of the compounds or DMSO as a control were injected followed by three measurements. Subsequently, oligomycin A, FCCP and rotenone/antimycin A were injected sequentially. Three measurements were performed after each injection. Using the Wave software Version 2.6.0 (Agilent, USA), the background was subtracted from all data. Values were normalized to the last baseline measurement of each condition, which was set to 100°%

MitoStress Test after Preincubation

$2x10^5$ U-2OS cells were seeded per well into XFp cell culture mini plate prior to incubation overnight at 37°C, 5°% CO_2. The XFp cartridges were hydrated using the XF Calibrant and incubated overnight at 37°C. The cell medium was then replaced with cell culture medium containing the compounds or DMSO as a control. After 20 h of compound pre-incubation the medium was exchanged for pH 7.4 DMEM-based assay medium (Agilent, USA) containing 2 mM GlutaMAX (ThermoFisher), 1°mM sodium pyruvate (PAN Biotech, Germany) and 25 mM glucose (SigmaAldrich, Germany) and the compounds, and cells were incubated at 37°C without CO_2 for 45 min. Five baseline recordings were acquired before subsequent sequential injection of oligomycin A, FCCP and rotenone/antimycin A. Three measurements were performed after each injection.

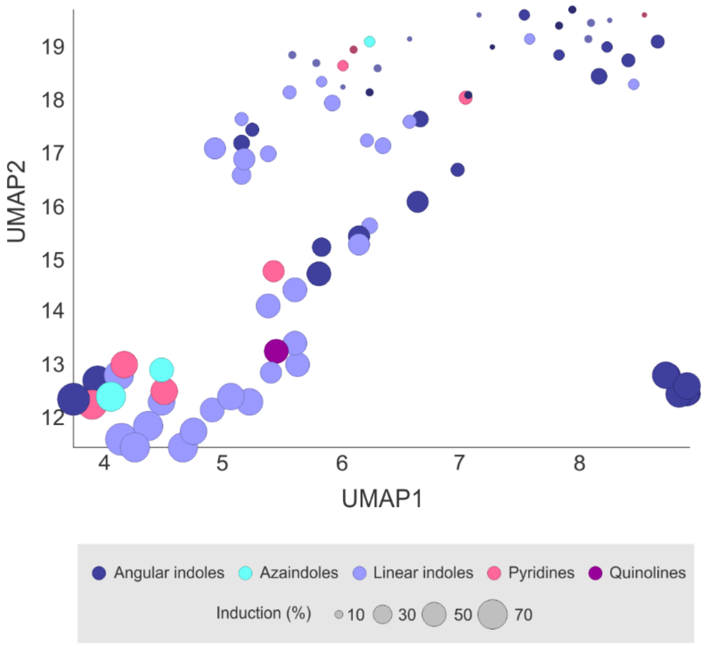

Figure S16. Profile analysis of the PNP collection based on the substructures. UMAP plot using the profiles for all active PNPs at different concentrations. The size of the symbols depicts the induction value. Not normalized, 10 neighbors.

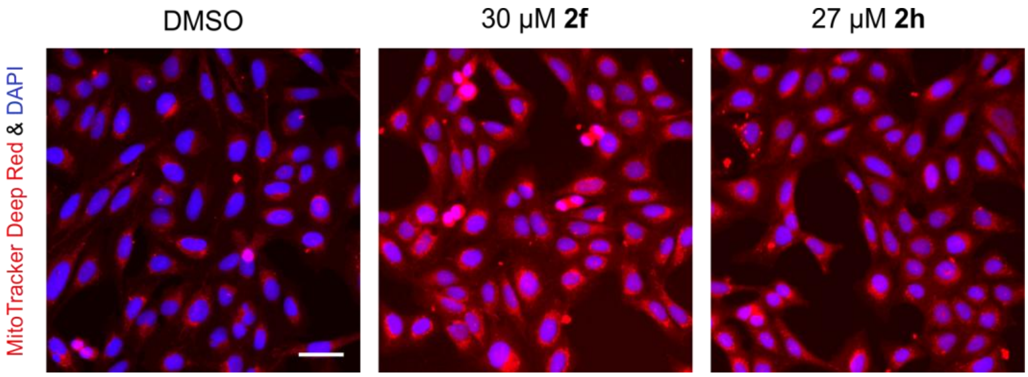

Figure S17. MitoTracker Deep Red and nuclear staining (DAPI) for **2f** and **2h**. Images from CPA are shown. Scale bar: 50 µm.

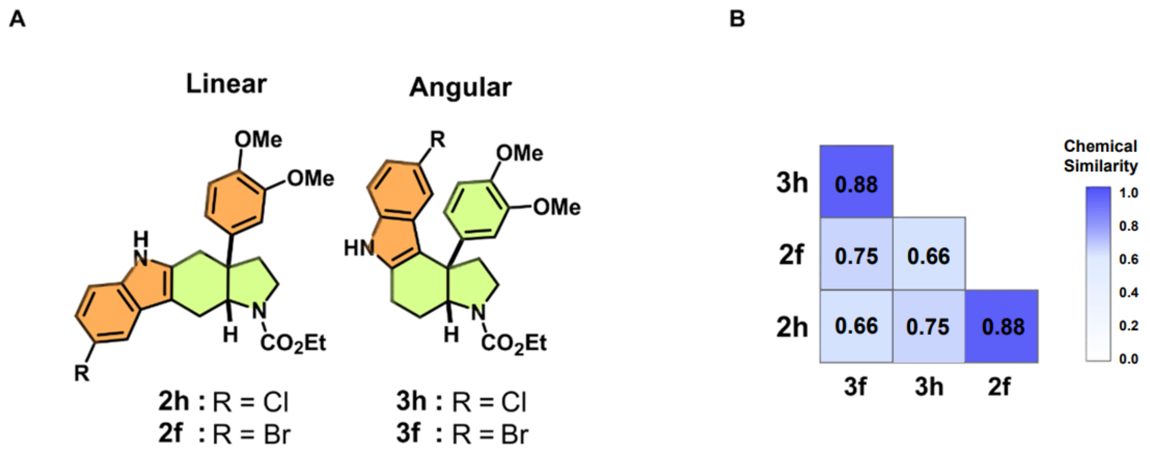

Figure S18. Chemical similarity was determined using the Tanimoto score of the Morgan fingerprints with radius 2, as implemented in the RDKit. (A) Linear and angular indole fused AOHIs. (B) Chemical similarity is shown as AP Tanimoto similarities.

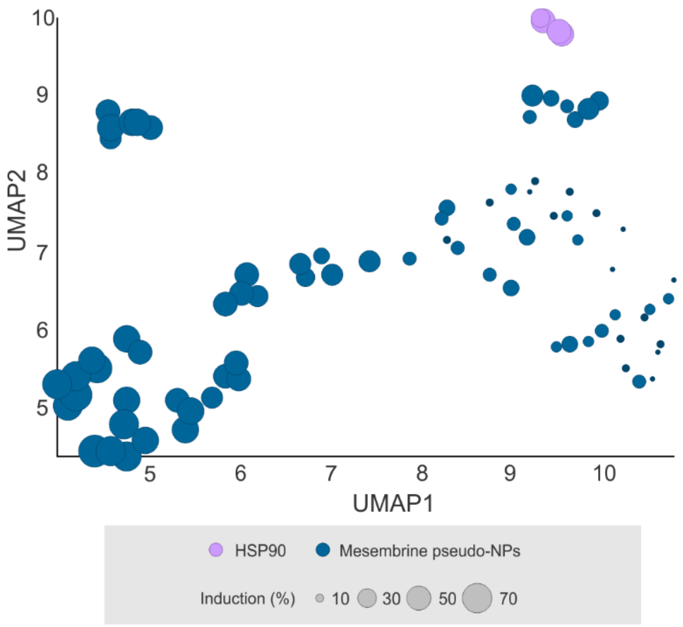

Figure S19. Profile analysis of the PNP collection and HSP90 inhibitors. UMAP plot using the profiles for all active PNPs at different concentrations and the profiles of HSP90 inhibitors. The size of the symbols depicts the induction value. Not normalized, 10 neighbors.

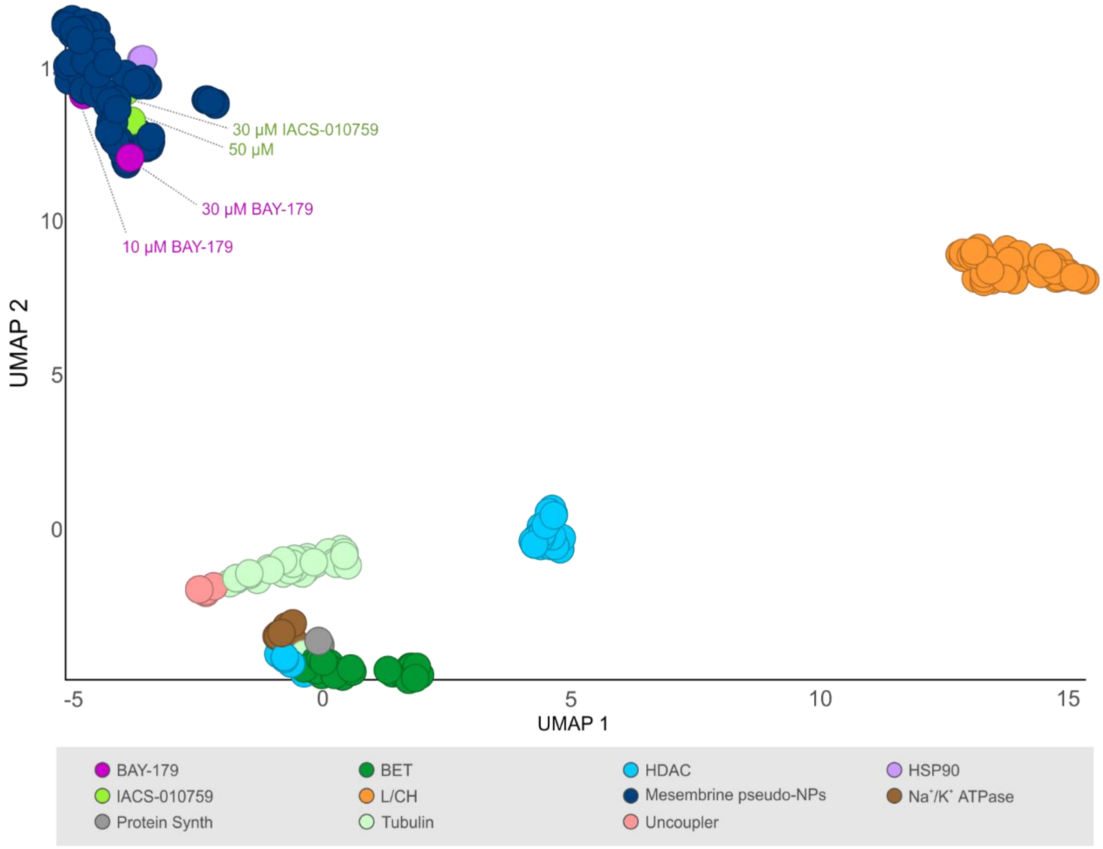

Figure S20. Analysis of the profiles of BAY-179 and IACS-010759. UMAP plot using the profiles of BAY-179 and IACS-010759 at different concentrations and along with selected clusters. Not normalized, 10 neighbors. L/CH: lysosomotropism/cholesterol homeostasis.

A

	Cpd	Conc [µM]	AKT/PI3K/MTOR	Aurora	BET	DNA synthesis	HDAC	HSP90	L/CH	MitoStress	Na^+/K^+ ATPase	Protein synthesis	PYR synthesis	Tubulin	Uncoupling
			**Cluster biosimilarities**												
**complex III**	Myxothiazol	30	63	0	0	83	0	26	0	61	0	0	93	0	0
	Pyraclostrobin	50	67	0	0	77	0	6	0	16	0	0	88	0	0
**complex V**	Oligomycin A	10	16	0	25	0	23	43	35	83	18	28	0	38	25
**uncoupling**	FCCP	10	24	0	60	0	60	3	42	0	40	48	0	53	96
	AG879	10	43	0	45	0	35	19	38	18	25	37	0	55	89

B

complex		I		III		V	uncoupling
		BAY-179 (10 µM)	IACS-010759 (30 µM)	Myxothiazol (10 µM)	Pyraclostrobin (10 µM)	Oligomycin A (10 µM)	FCCP (10 µM)
**complex I**	IACS-010759 (30 µM)	78					
**complex III**	Myxothiazol (10 µM)	0	0				
	Pyraclostrobin (10 µM)	0	0	77			
**complex V**	Oligomycin A (10 µM)	41	53	33	0		
**uncoupling**	FCCP (10 µM)	31	45	0	0	0	
	AG879 (10 µM)	30	48	0	1	31	76

Figure S21. Profile analysis of ETC inhibitors. (A) Cluster biosimilarity heatmap for compound for selected ETC inhibitors. Biosimilarities are given as percentage values. L/CH: lysosomotropism/cholesterol homeostasis; PYR: pyrimidine. (B) Profile biosimilarity cross-correlation for ETC inhibitors.

A

Cpd	Ind [%]	Conc [µM]	Cell count [%]	AKT/PI3K/MTOR	Aurora	BET	DNA synthesis	HDAC	HSP90	L/CH	MitoStress	Na^+/K^+ ATPase	Protein synthesis	PYR synthesis	Tubulin	Uncoupling
**7c**	6	3	108	0	0	44	0	41	16	61	62	15	13	0	39	5
	20	10	105	0	0	28	0	33	34	32	41	23	13	0	34	5
	61	30	99	0	0	58	0	64	39	56	37	45	46	0	59	41
	73	50	81	0	0	72	0	76	38	72	31	48	54	0	70	54

Cluster biosimilarities (colour scale: 100, 90, 80)

B

ETC inhibitors		**7c** (30 µM)	**7c** (50 µM)
**complex I**	BAY-179 (10 µM)	74	80
	BAY-179 (30 µM)	75	89
	IACS-010759 (30 µM)	68	78
	IACS-010759 (50 µM)	79	82
**complex III**	Myxothiazol (10 µM)	0	0
	Pyraclostrobin (10 µM)	0	0
**complex V**	Oligomycin A (10 µM)	55	46
**uncoupling**	FCCP (10 µM)	13	31
	AG879 (10 µM)	18	29

Figure S22. Profile analysis for compound **7c**. (A) Cluster biosimilarity heatmap for compound **7c**. Biosimilarities are given as percentage values. L/CH: lysosomotropism/ cholesterol homeostasis; PYR: pyrimidine. (B) Profile similarity for compound **7c** and ETC inhibitors.

References

[1] M. T. Reetz, H. Guo, J. A. Ma, R. Goddard, R. J. Mynott, *J. Am. Chem. Soc.* **2009**, *131*, 4136-4142.

[2] A. Padwa, M. A. Brodney, M. Dimitroff, B. Liu, T. Wu, *J. Org. Chem.* **2001**,*66*, 3119−3128.

[3] Q. Huang, A. Fazio, G. Dai, M. A. Campo, R. C. Larock, *J. Am. Chem. Soc.* **2004**, *126*, 7460−7461.

[4] a) C. Knox, M. Wilson, C. M. Klinger, M. Franklin, E. Oler, A. Wilson, A. Pon, J. Cox, N. E. L. Chin, S. A. Strawbridge, M. Garcia-Patino, R. Kruger, A. Sivakumaran, S. Sanford, R. Doshi, N. Khetarpal, O. Fatokun, D. Doucet, A. Zubkowski, D. Y. Rayat, H. Jackson, K. Harford, A. Anjum, M. Zakir, F. Wang, S. Tian, B. Lee, J. Liigand, H. Peters, R. Q. R. Wang, T. Nguyen, D. So, M. Sharp, R. da Silva, C. Gabriel, J. Scantlebury, M. Jasinski, D. Ackerman, T. Jewison, T. Sajed, V. Gautam, D. S. Wishart, *Nucleic Acids Res.* **2024**, *52*, D1265-D1275. b) V. C. Nainala, S. R. S. Kanakam, N. Sharma, V. Weißenborn, J. Schaub, C. Steinbeck, K. Rajan, *Zenodo*, **2024**, v1, DOI: 10.5281/zenodo.13382751.

[5] https://enamine.net/hit-finding/compound-collections/screening-collection/advanced-collection; downloaded 07-Dec-2020.

[6] B. Zdrazil, E. Felix, F. Hunter, E. J. Manners, J. Blackshaw, S. Corbett, M. de Veij, H. Ioannidis, D. M. Lopez, J. F. Mosquera, M. P. Magarinos, N. Bosc, R. Arcila, T. Kiziloren, A. Gaulton, A. P. Bento, M. F. Adasme, P. Monecke, G. A. Landrum, A. R. Leach, *Nucleic Acids Res.* **2024**, *52*, D1180-D1192.

[7] M. Sorokina, P. Merseburger, K. Rajan, M. A. Yirik, C. Steinbeck, *J. Cheminform* **2021**, *13*, 2

[8] P. Balazs, T. Jeppe, G. Bajusz, A. Kafer, E. L. Willighagen, *BMC Bioinf.* **2013**, *14*, 257.

[9] M. Grigalunas, A. Burhop, S. Zinken, A. Pahl, J. M. Gally, N. Wild, Y. Mantel, S. Sievers, D. J. Foley, R. Scheel, C. Strohmann, A. P. Antonchick, H. Waldmann, *Nat. Commun.* **2021**, *12*, 1883.

[10] P. Ertl, S. Roggo, A. Schuffenhauer, *J. Chem. Inf. Model.* **2008**, *48*, 68.

[11] G. R. Bickerton, G. V. Paolini, J. Besnard, S. Muresan, A. L. Hopkins, *Nat. Chem.* **2012**, *4*, 90.

[12] T. Bottcher, *J. Chem. Inf. Model,* **2016**, *56*, 462-470.

[13] R. M. Demoret, M. A. Baker, M. Ohtawa, S. Chen, C. C. Lam, S. Khom, M. Roberto, S. Forli, K. N. Houk, R. A. Shenvi, *J. Am. Chem. Soc.* **2020**, *142*, 18599-18618.

[14] A. Krzyzanowski, A. Pahl, M. Grigalunas, H. Waldmann, *J. Med. Chem.* **2023**, *66*, 12739-12750.

[15] W. H. B. Sauer, M. K. Schwarz, *J. Chem. Inf. Comput. Sci*. **2003**, *43*, 987-1003.

[16] RDKit: Open-Source Cheminformatics; http://www.rdkit.org.

[17] A. K. Rappe, C. J. Casewit, K. S. Colwell, W. A. Goddard III, W. M. Skiff; *J. Am. Chem. Soc.* **1992**, *114*, 10024–10035.

[18] Bray, M. A., Singh, S., Han, H., Davis, C. T., Borgeson, B., Hartland, C. *et al*. *Nat. Protoc.* **2016**, *11*, 1757-1774.

[19] A. Pahl, B. Scholermann, P. Lampe, M. Rusch, M. Dow, C. Hedberg, A. Nelson, S. Sievers, H. Waldmann, S. Ziegler, *Cell Chem. Biol.* **2023**, *30*, 839-853 e837.

[20] Carpenter, A. E., Jones, T. R., Lamprecht, M. R., Clarke, C., Kang, I. H., Friman, O. *et al*. *Genome Biol.* **2006**, *7*, R100.

[21] Woehrmann, M. H., Bray, W. M., Durbin, J. K., Nisam, S. C., Michael, A. K., Glassey, E. *et al*. *Mol. Biosyst.* **2013**, *9*, 2604-2617.

[22] L. Wang, F. Yilmaz, O. Yildirim, B. Schölermann, S. Bag, L. Greiner, A. Pahl, S. Sievers, R. Scheel, C. Strohmann, C. Squire, D. J. Foley, S. Ziegler, M. Grigalunas, H. Waldmann, *Adv. Sci.* **2024**, *11*, 2309202.

NMR Spectra

General Notes:

1) Signals of the trifluoracetate anion were not included in the carbon assignment.
2) Rotameric signals of the carbamate group were shown in zoomed regions once or twice as representative examples
3) Depending on the Compound class, the magnitude of the carbamate-induced rotameric signal splitting varied, reflecting differences in topological asymmetry and conformational restriction between the classes.

Unsubstituted **C** $\Delta\delta$ = 0.25 ppm CH_2-N-CO_2Et

Angular splitting **3a** $\Delta\delta$ = 0.48 ppm CH_2-N-CO_2Et

Linear splitting **2a** $\Delta\delta$ = 0.54 ppm CH_2-N-CO_2Et

Linear splitting **7** $\Delta\delta$ = 0.33 ppm CH_2-N-CO_2Et

^{1}H-NMR of C (700 MHz, CDCl$_3$)

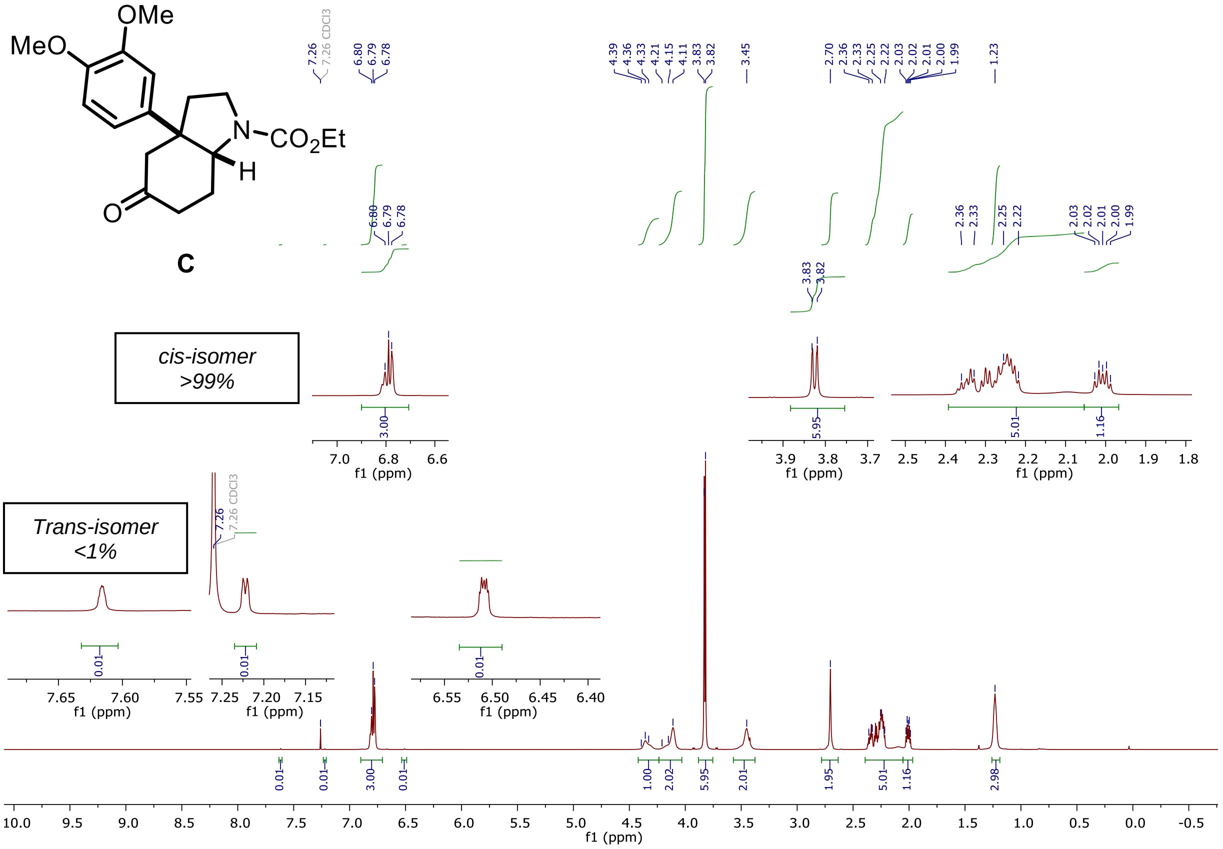

^{13}C-NMR of C (176 MHz, CDCl$_3$)

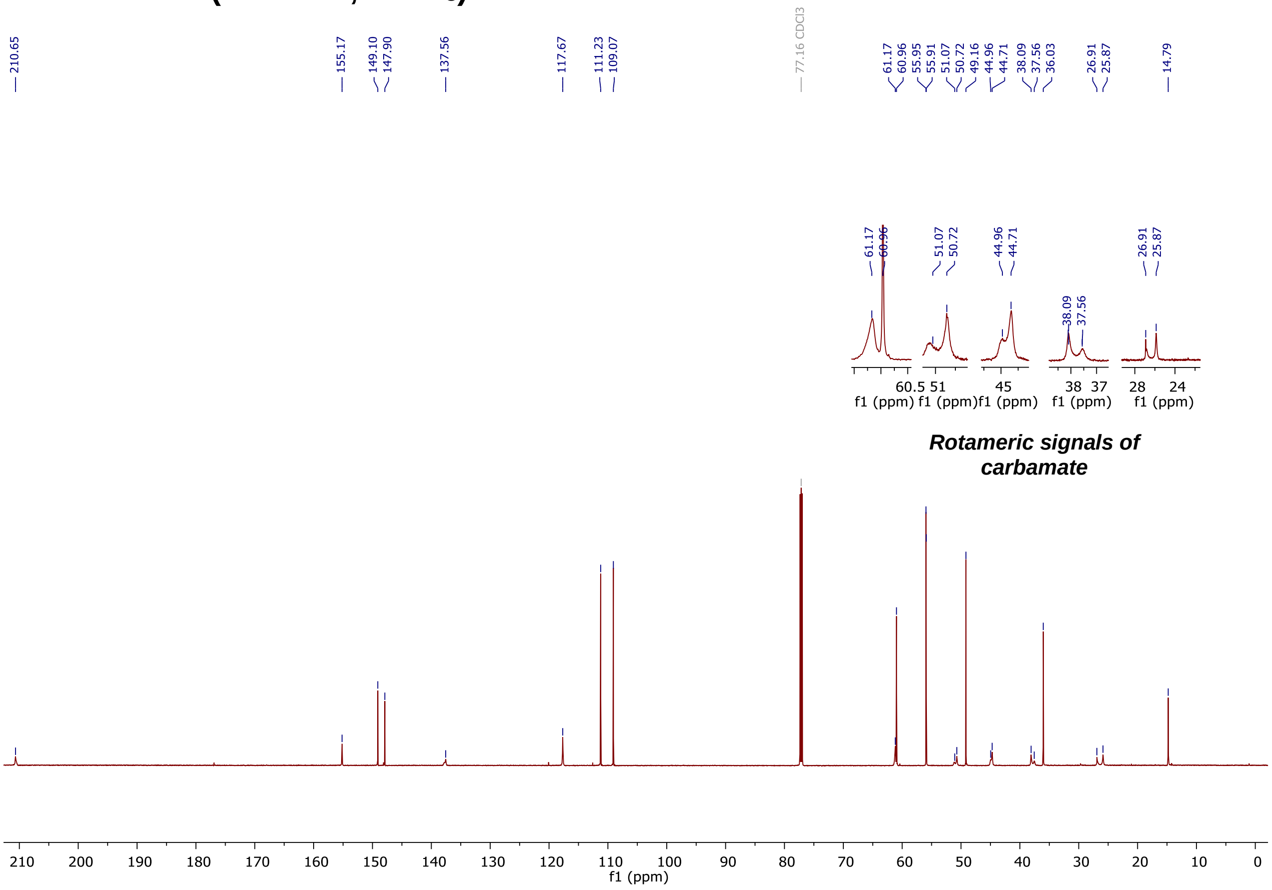

^{1}H-NMR of 2a (500 MHz, $CDCl_3$)

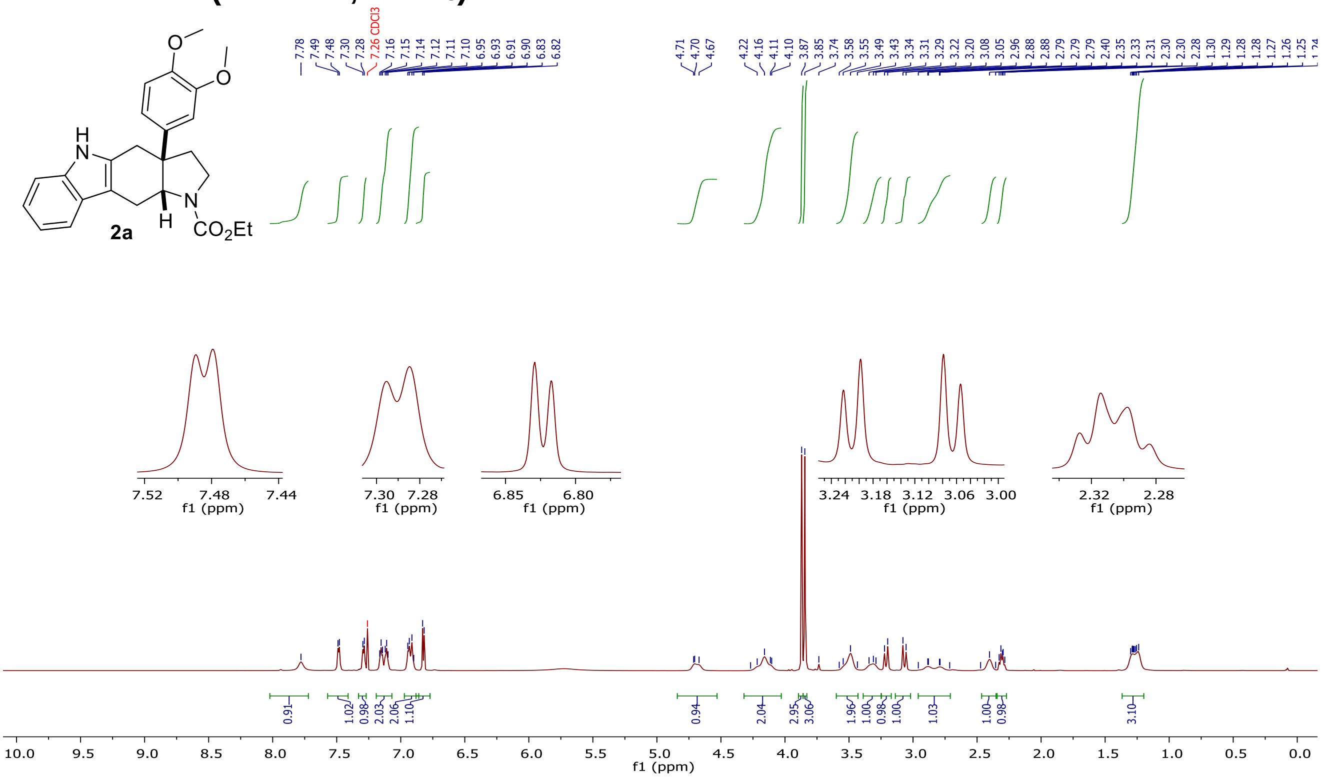

^{13}C-NMR of 2a (176 MHz, $CDCl_3$)

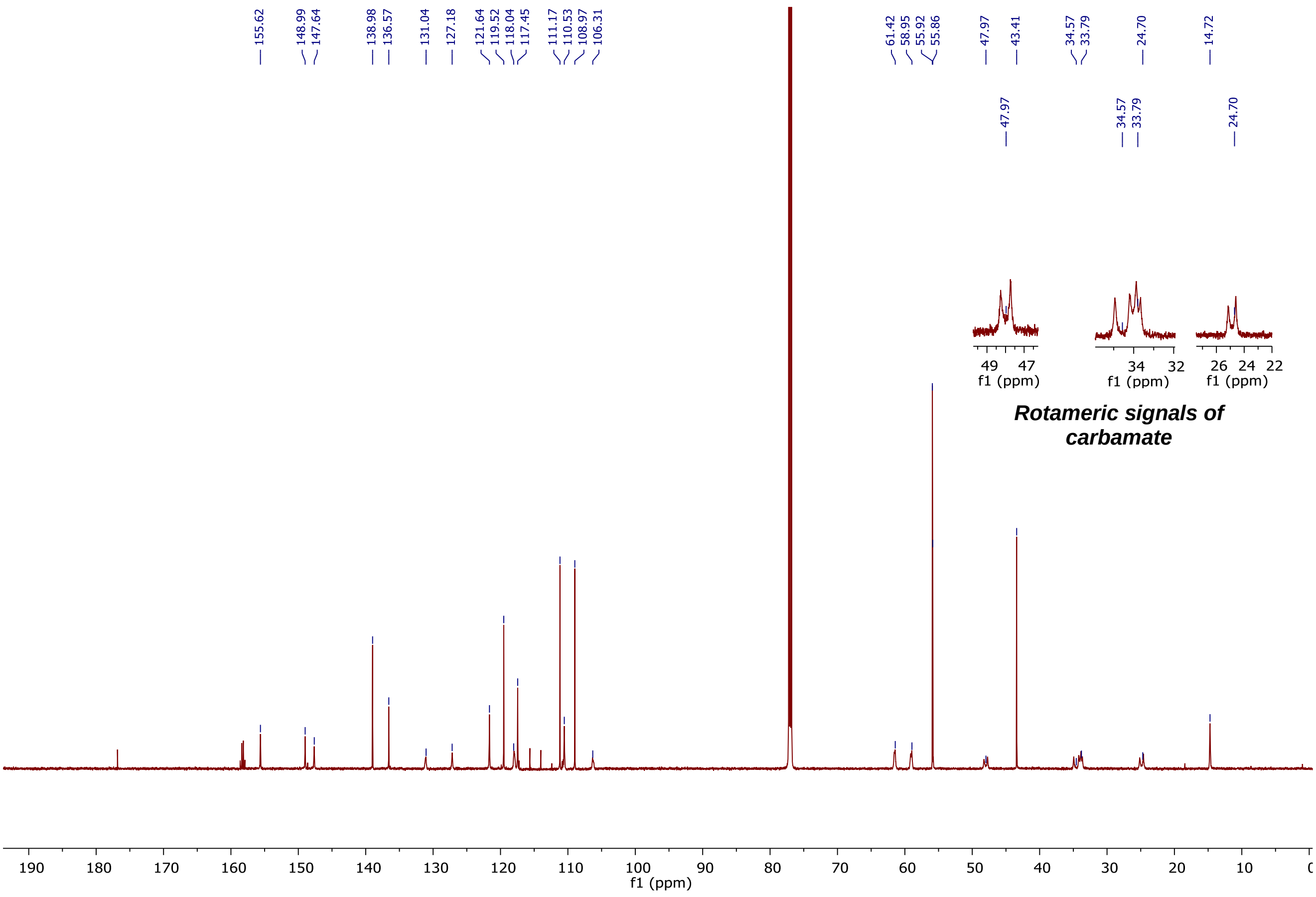

^{1}H-NMR of 2b (600 MHz, $CDCl_3$)

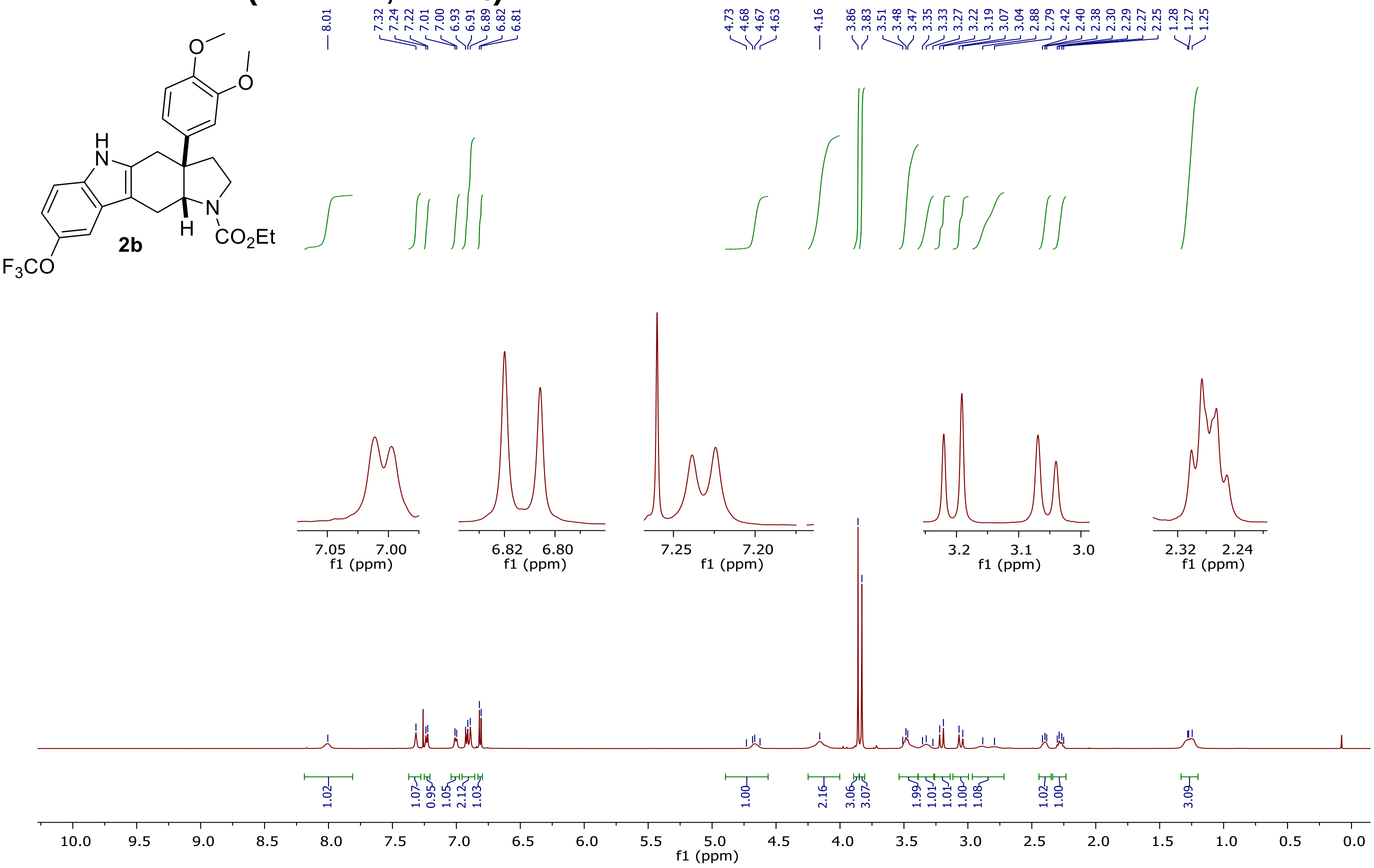

^{13}C-NMR 2b (176 MHz, $CDCl_3$)

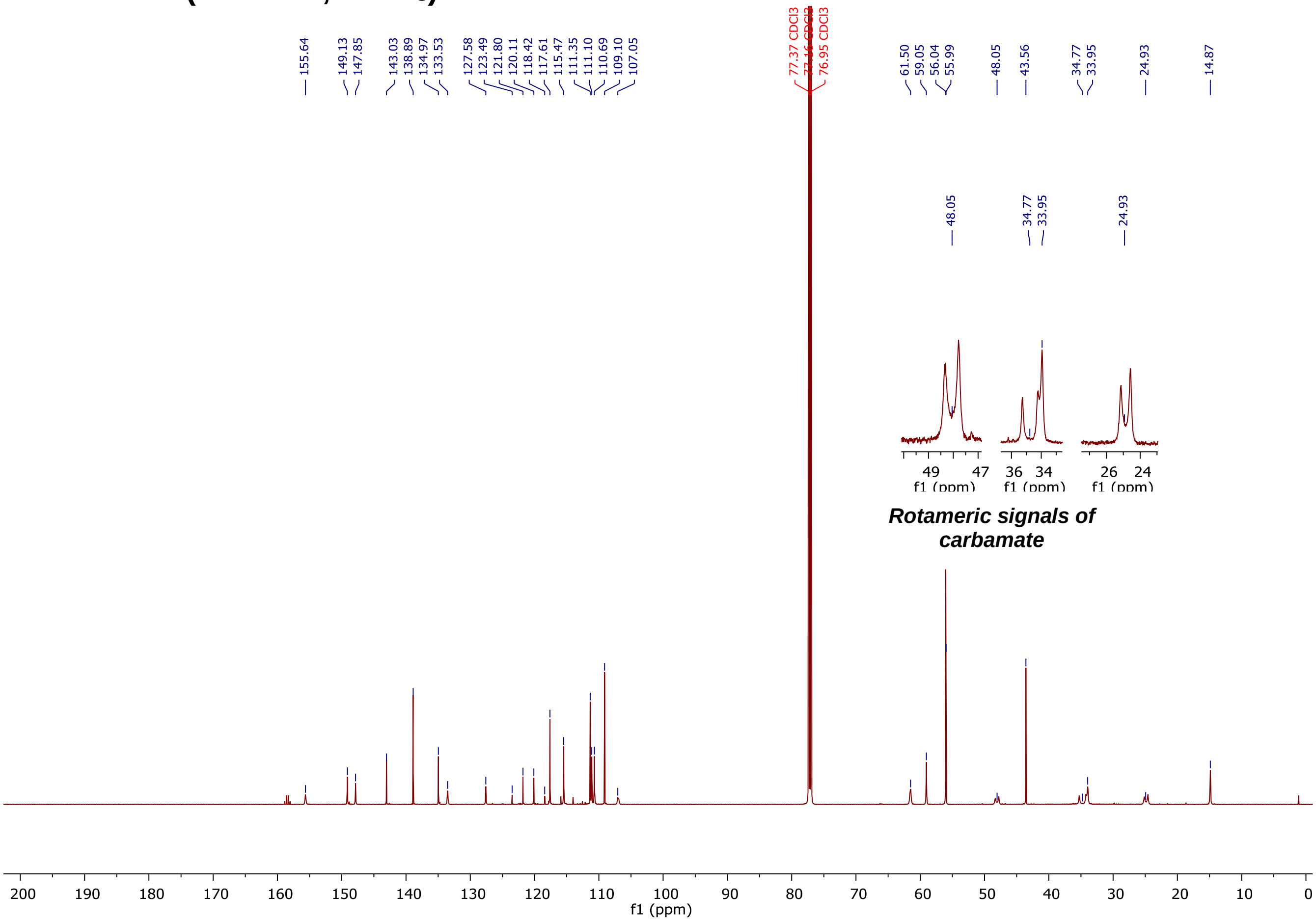

^{1}H-NMR of **2c (600 MHz, CDCl$_3$)**

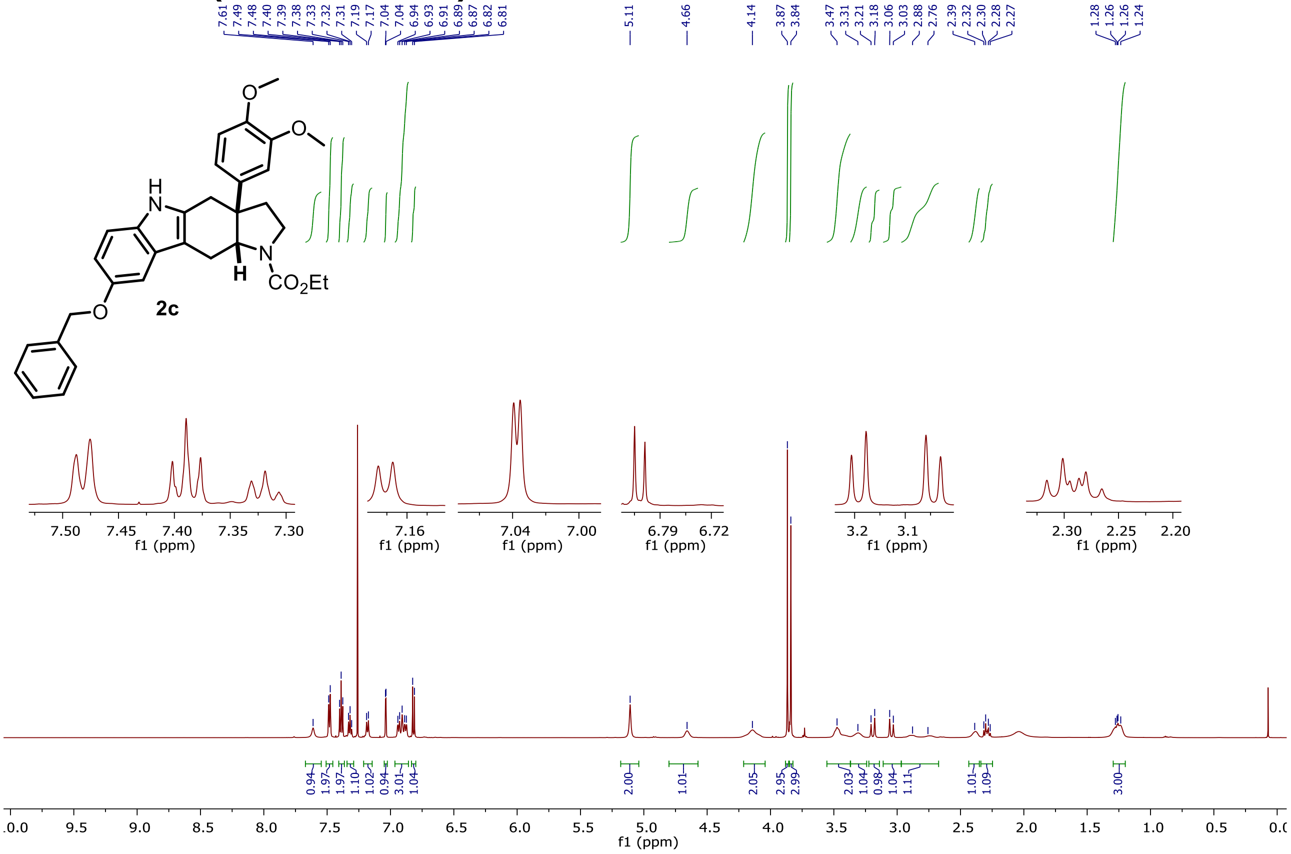

^{13}C-NMR of **2c (151 MHz, CDCl$_3$)**

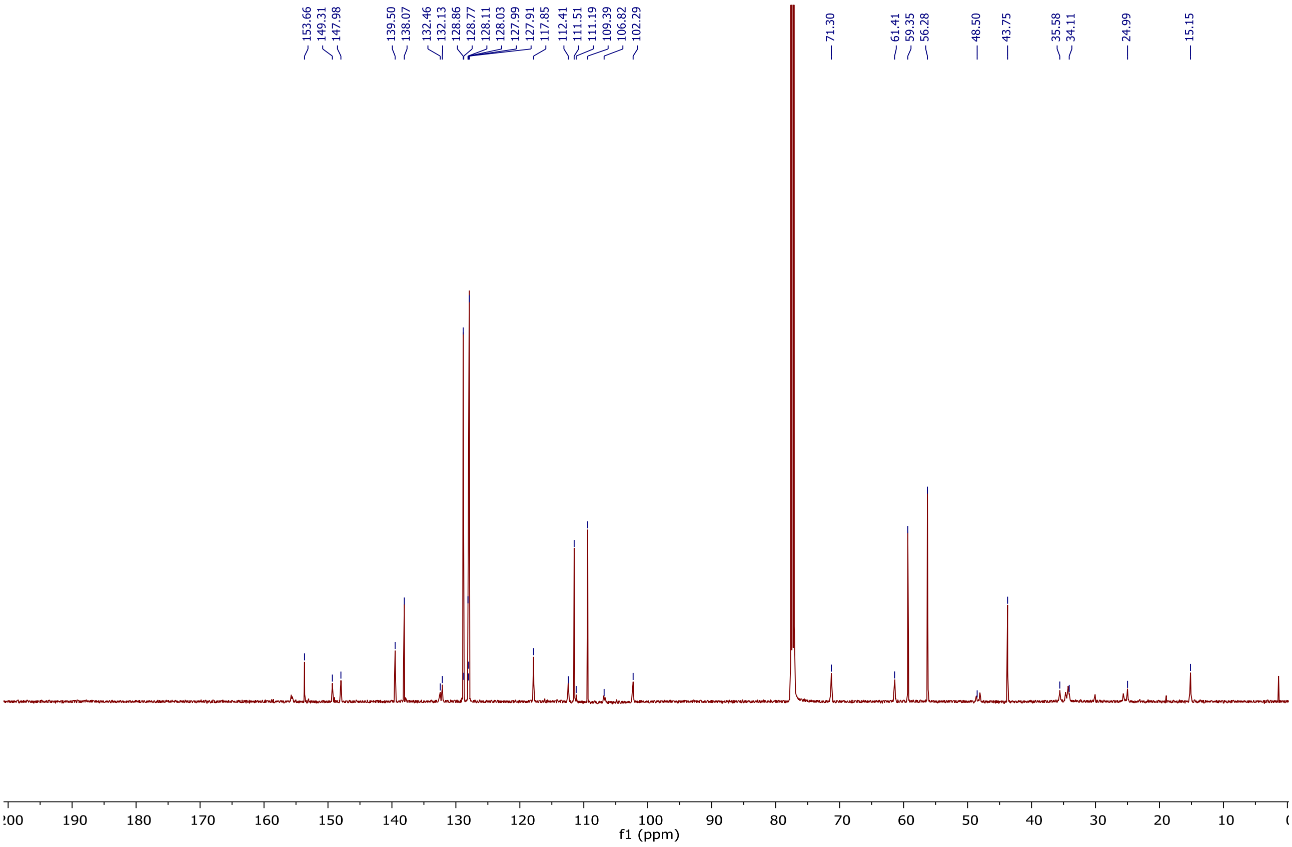

^{1}H-NMR of **2d** (600 MHz, $CDCl_3$)

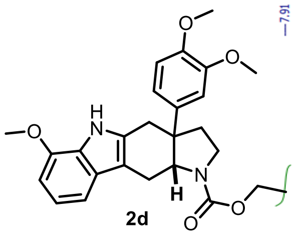

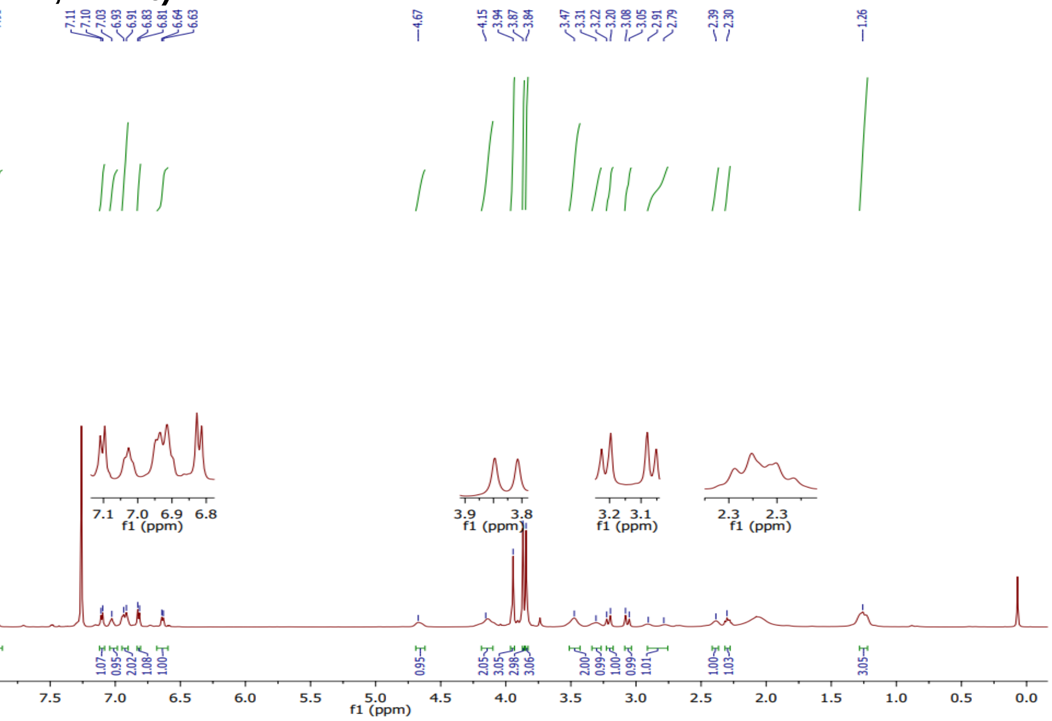

^{13}C-NMR of **2d** (151 MHz, $CDCl_3$)

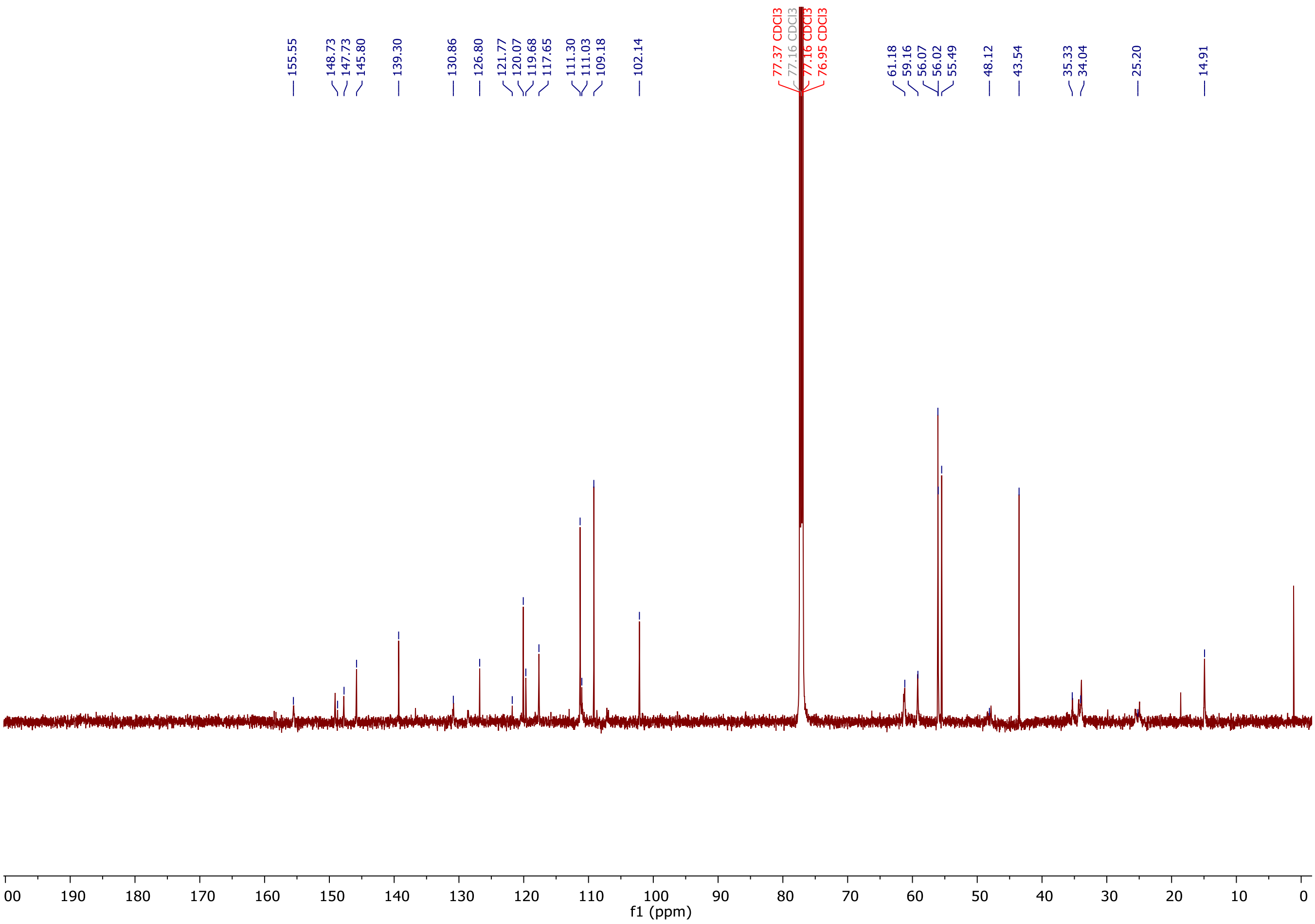

^{1}H-NMR of 2e (700 MHz, $CDCl_3$)

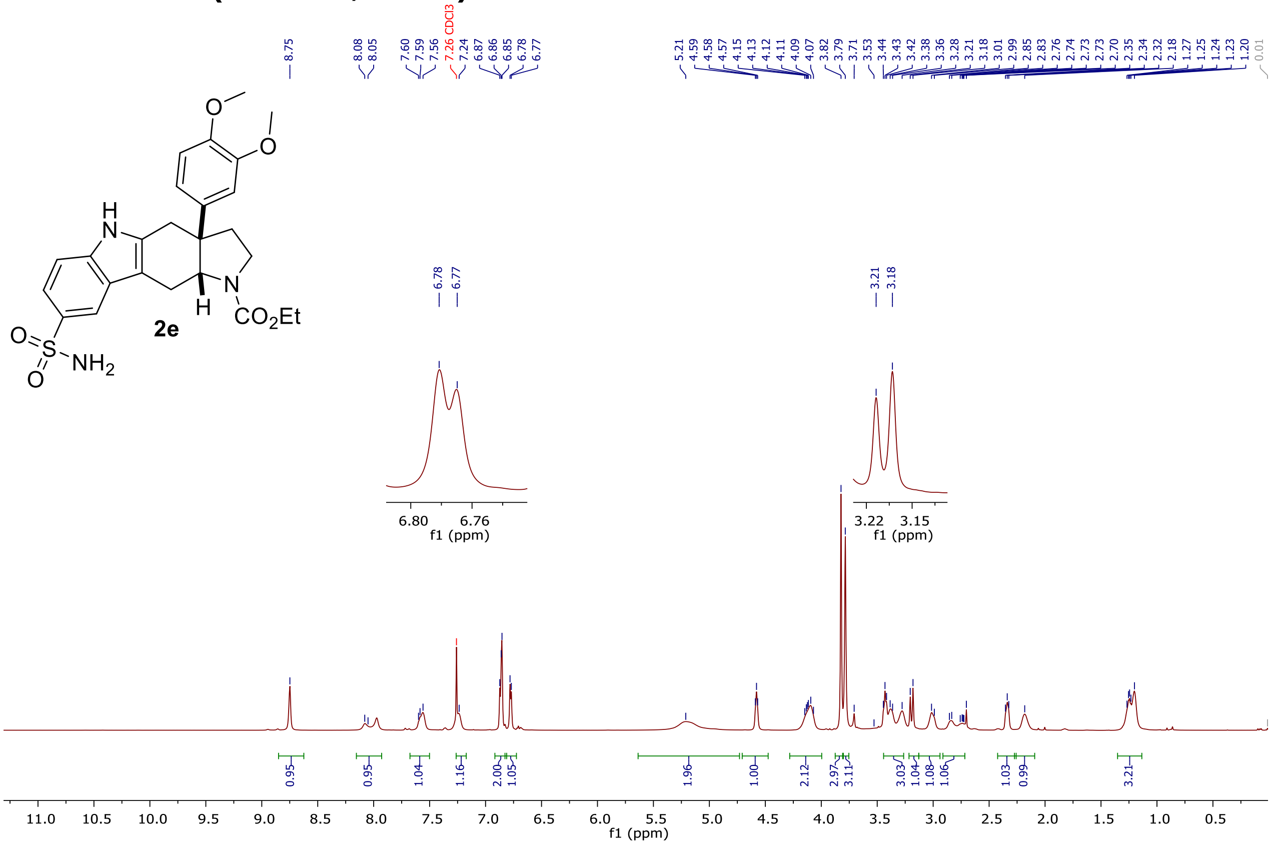

^{13}C-NMR of 2e (176 MHz, $CDCl_3$)

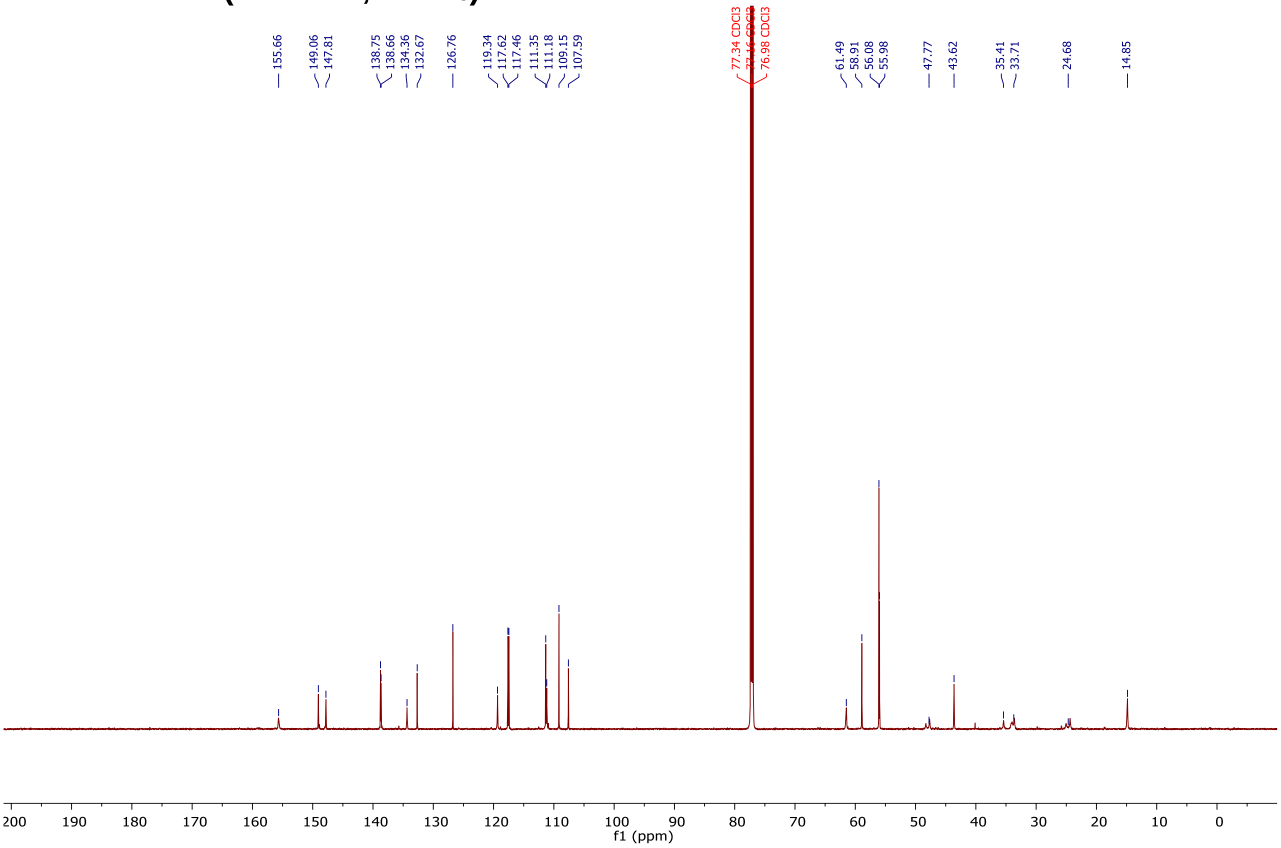

^{1}H-NMR of 2f (700 MHz, $CDCl_3$)

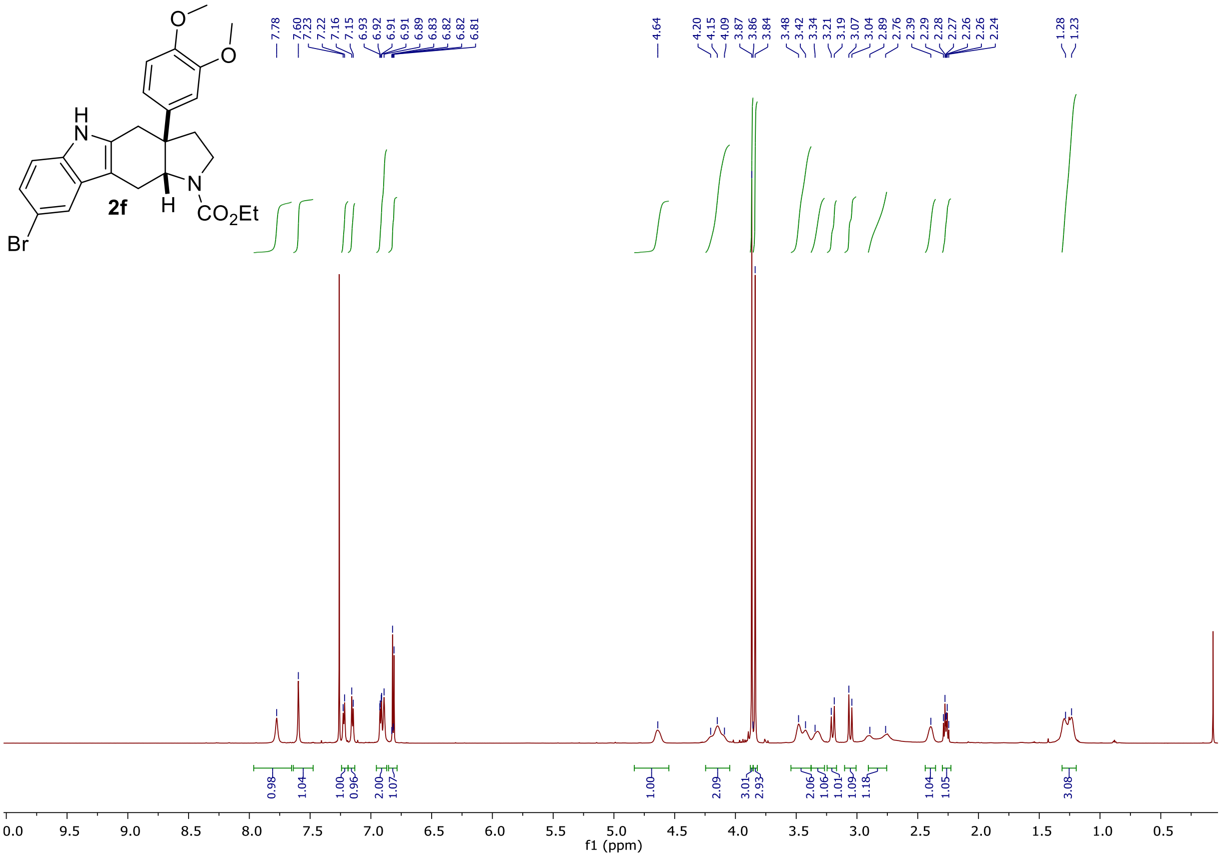

^{13}C-NMR of 2f (176 MHz, $CDCl_3$)

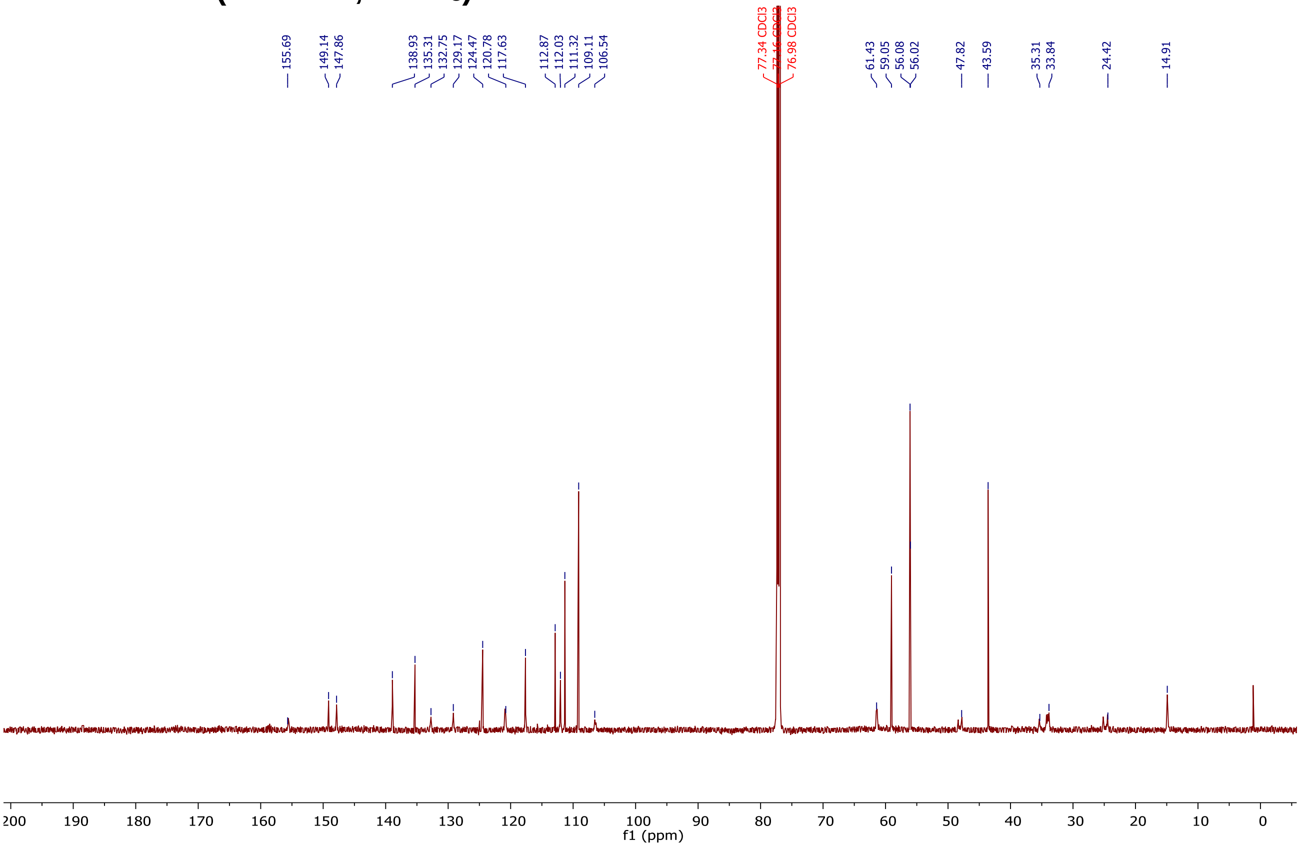

^{1}H-NMR of 2g (500 MHz, CDCl$_3$)

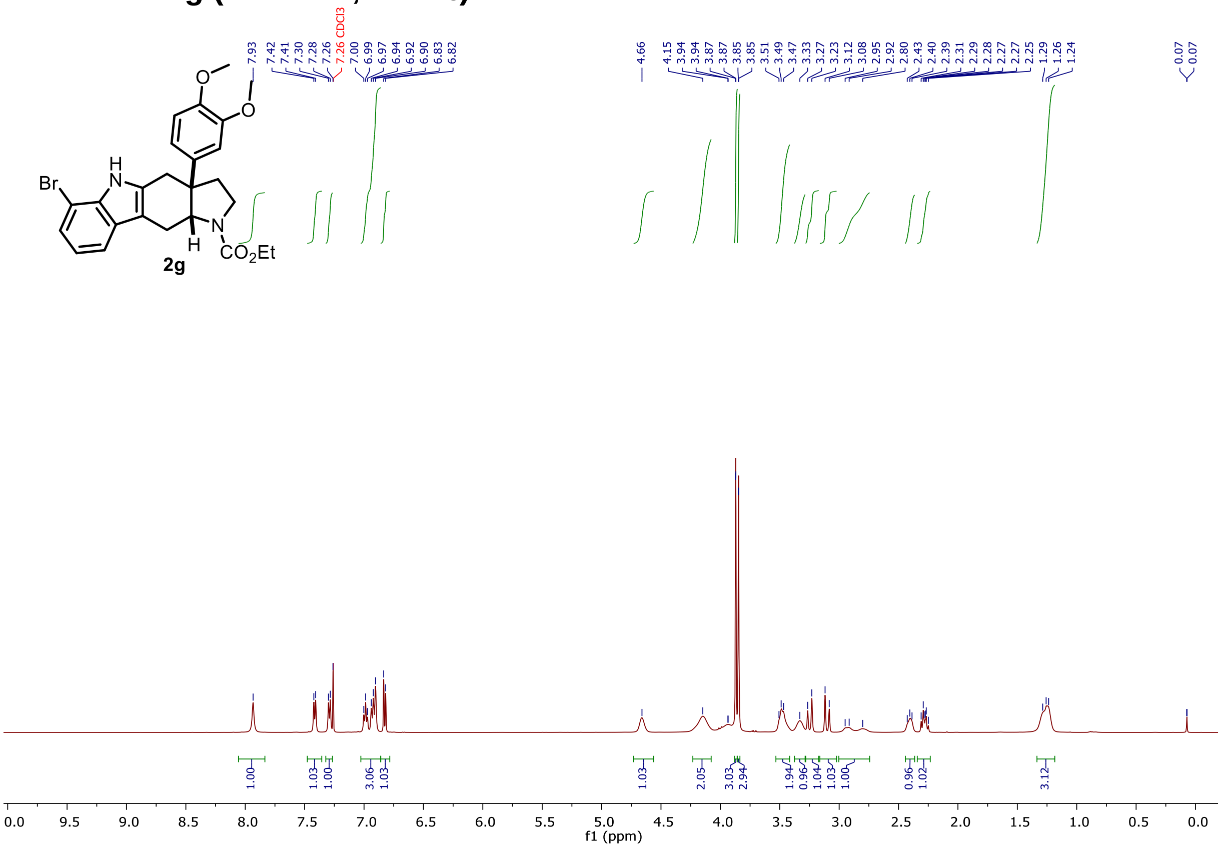

^{13}C-NMR of 2g (126 MHz, CDCl$_3$)

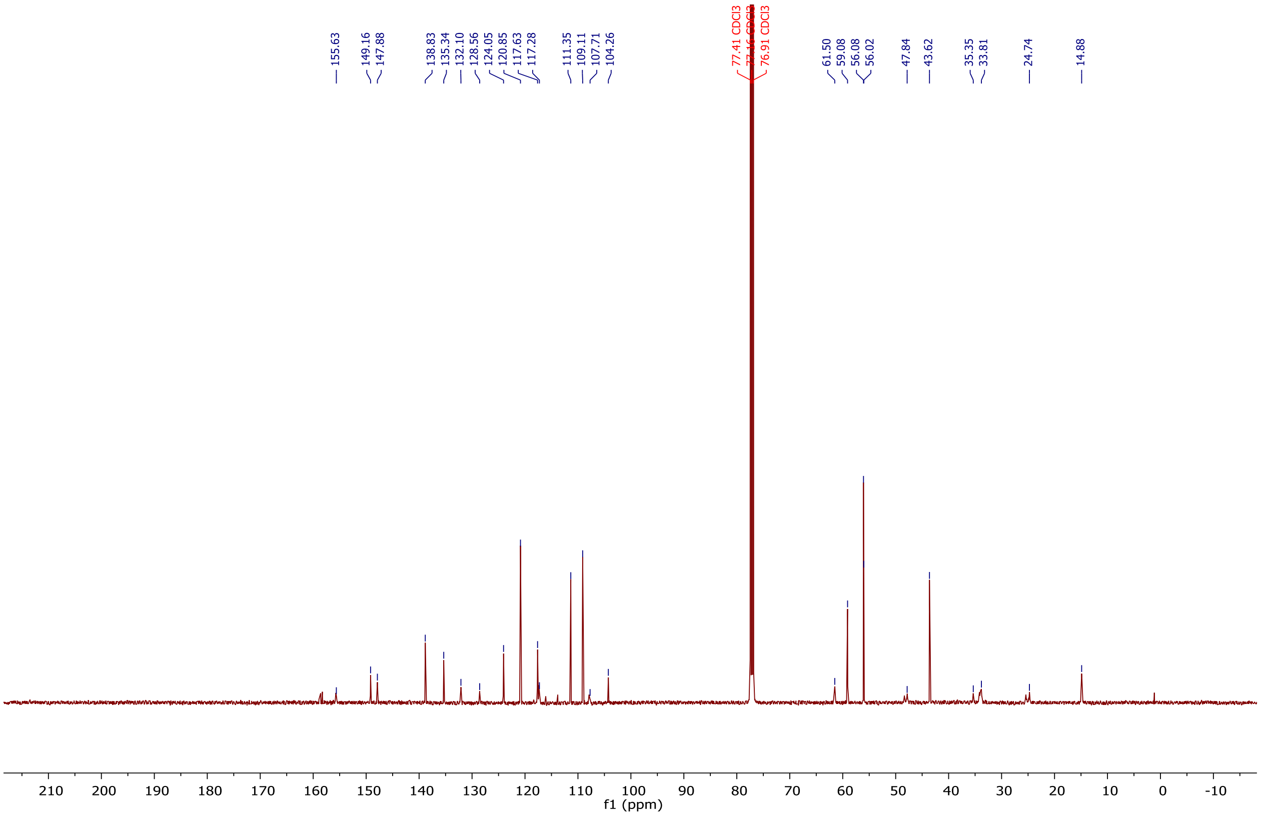

^{1}H-NMR of 2h (700 MHz, $CDCl_3$)

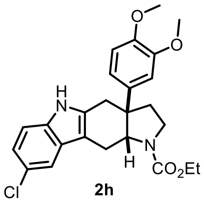

2h

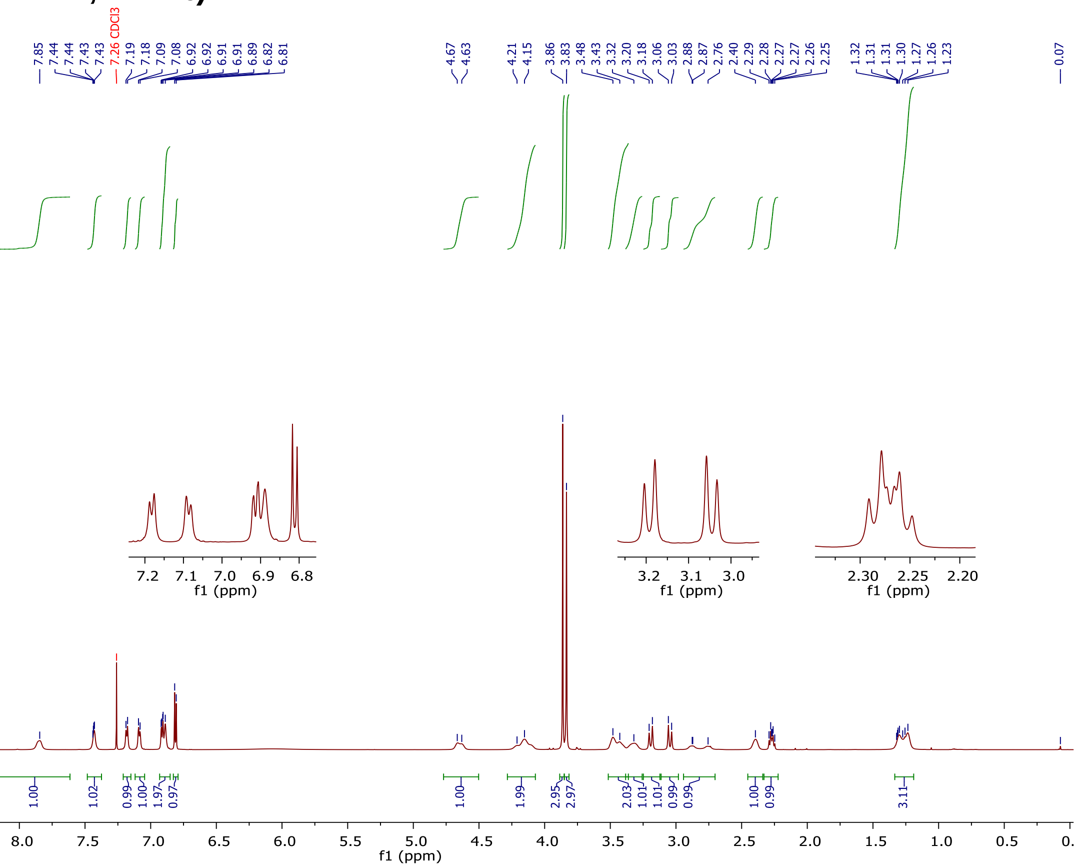

^{13}C-NMR of 2h (176 MHz, $CDCl_3$)

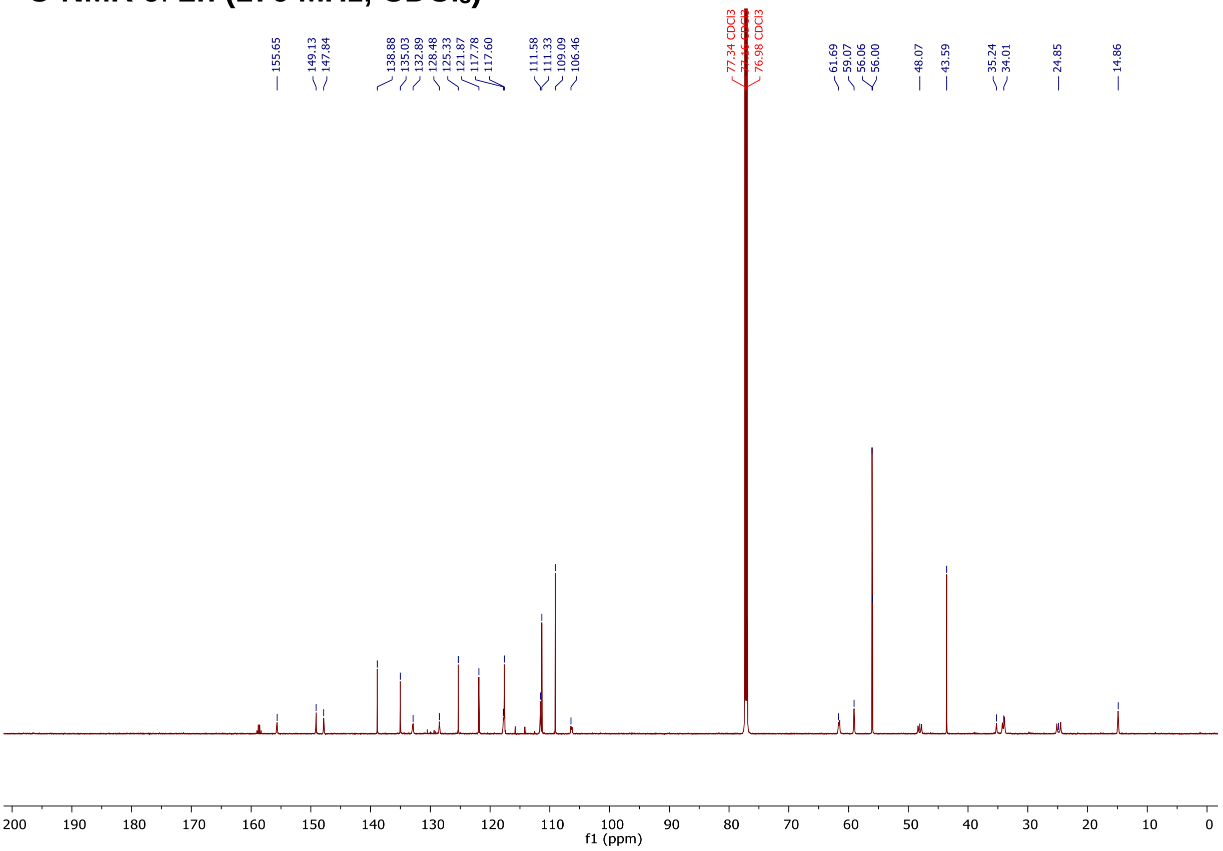

^{1}H-NMR of 2i (600 MHz, $CDCl_3$)

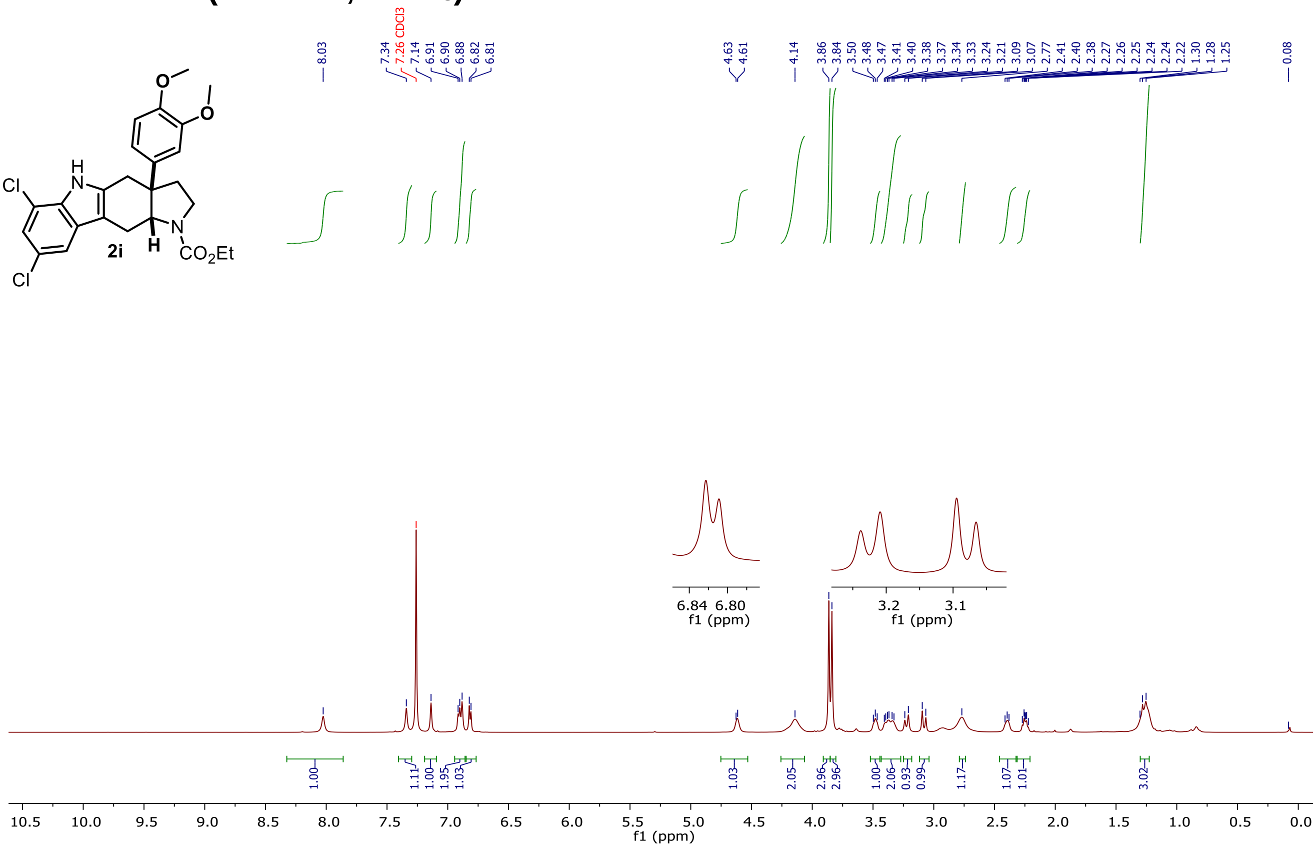

^{13}C-NMR of 2i (151 MHz, $CDCl_3$)

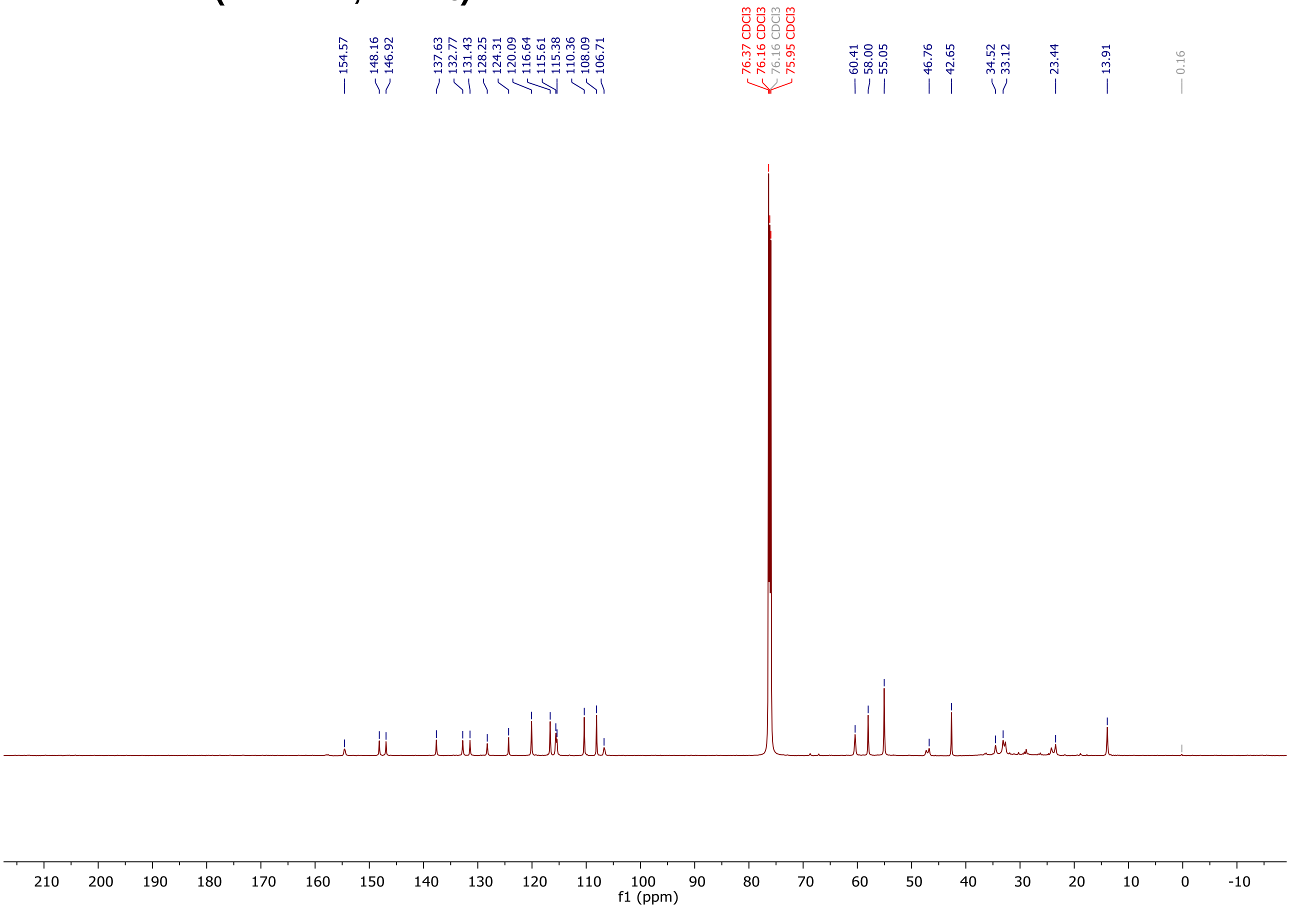

^{1}H-NMR of 2j (700 MHz, CDCl$_3$)

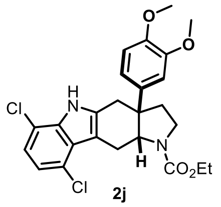

2j

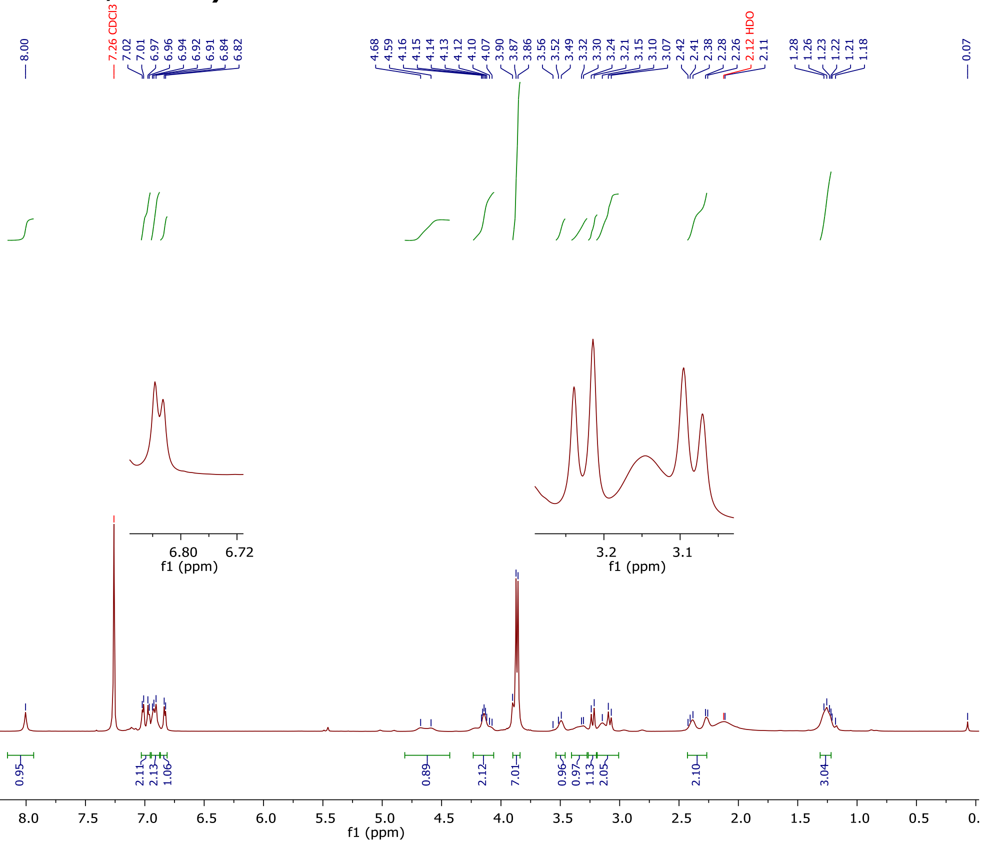

^{13}C-NMR of 2j (176 MHz, CDCl$_3$)

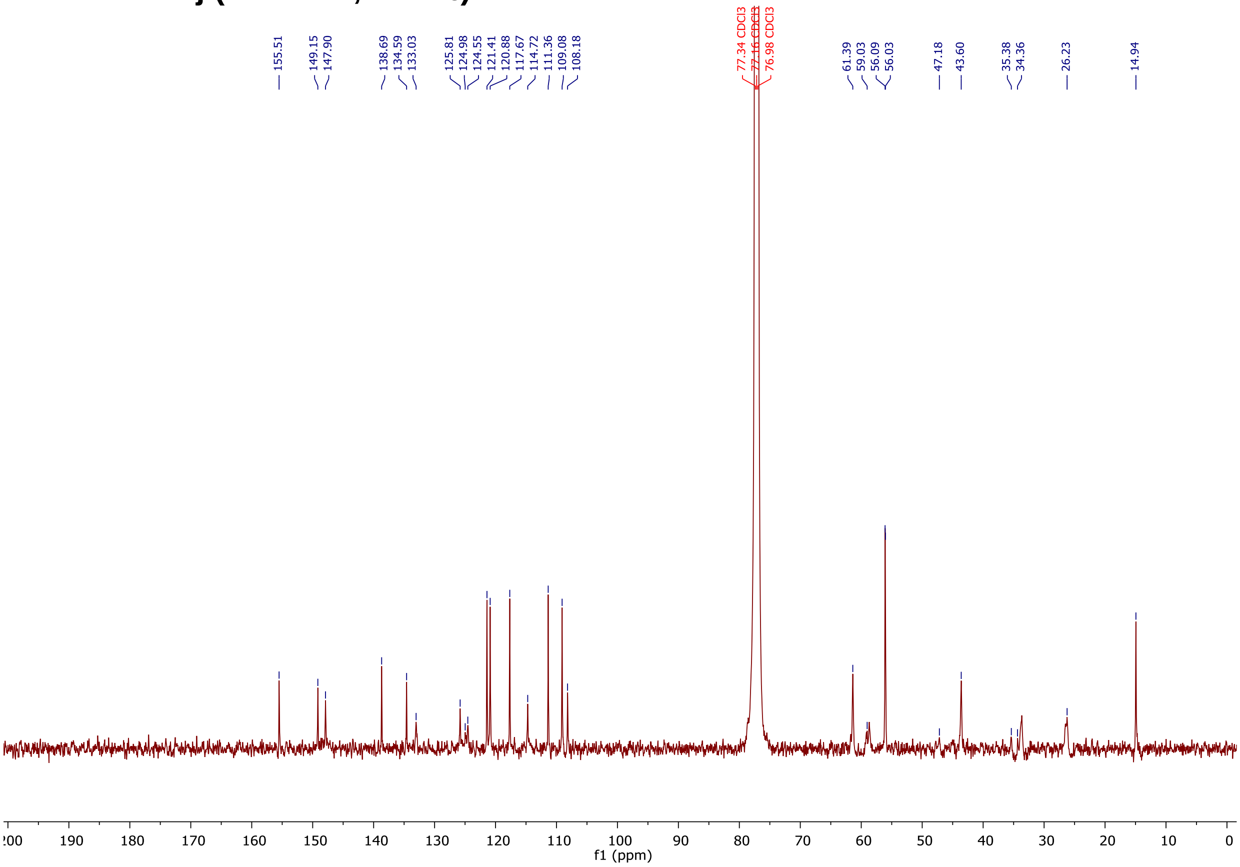

^{1}H-NMR of 2k (700 MHz, $CDCl_3$)

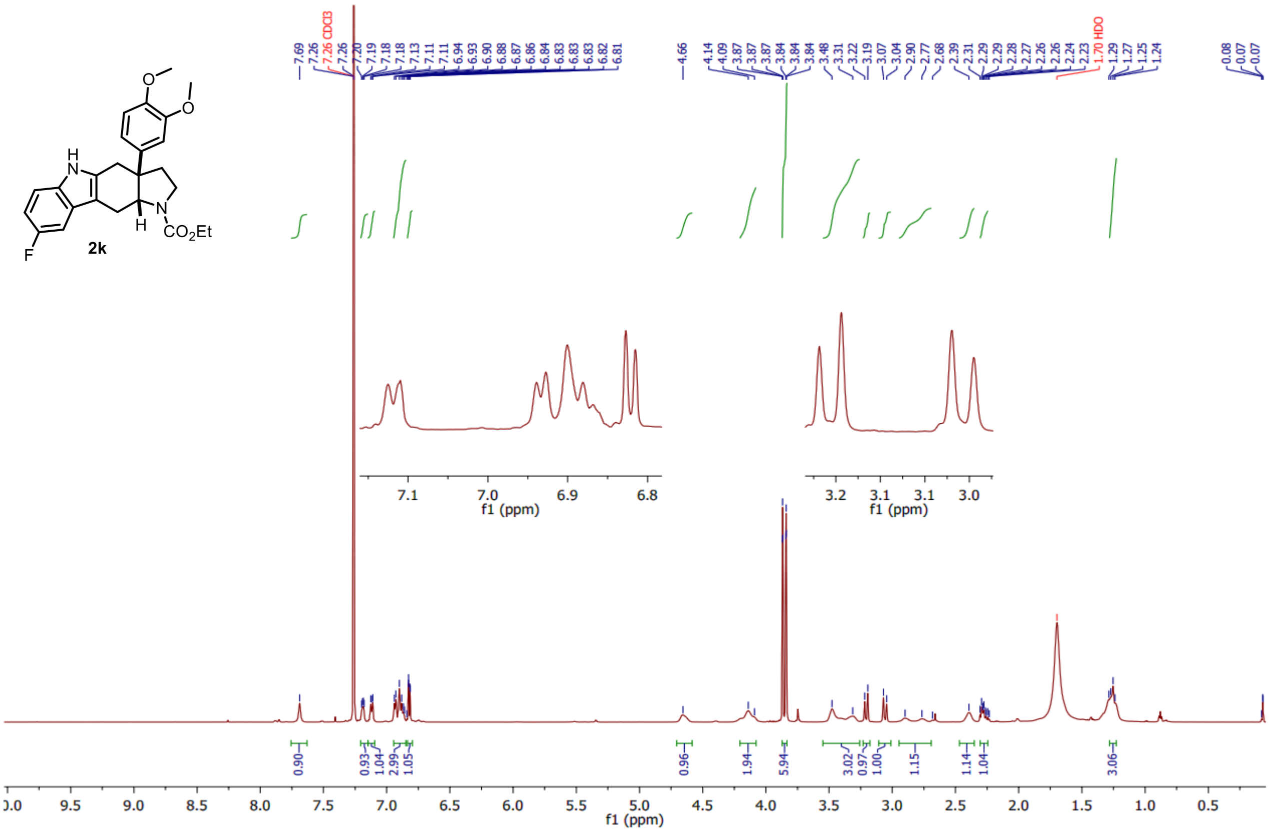

^{13}C-NMR of 2k (176 MHz, $CDCl_3$)

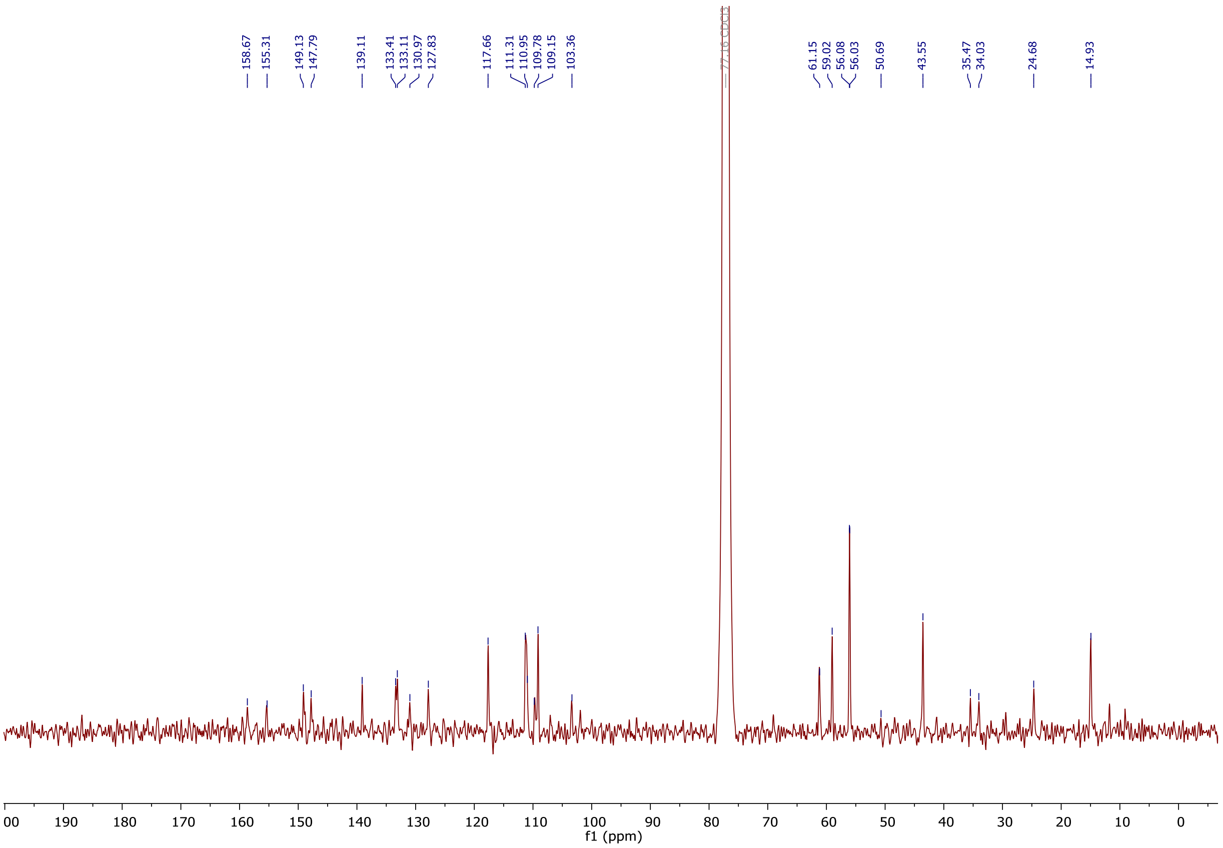

^{1}H-NMR of 2l (600 MHz, $CDCl_3$)

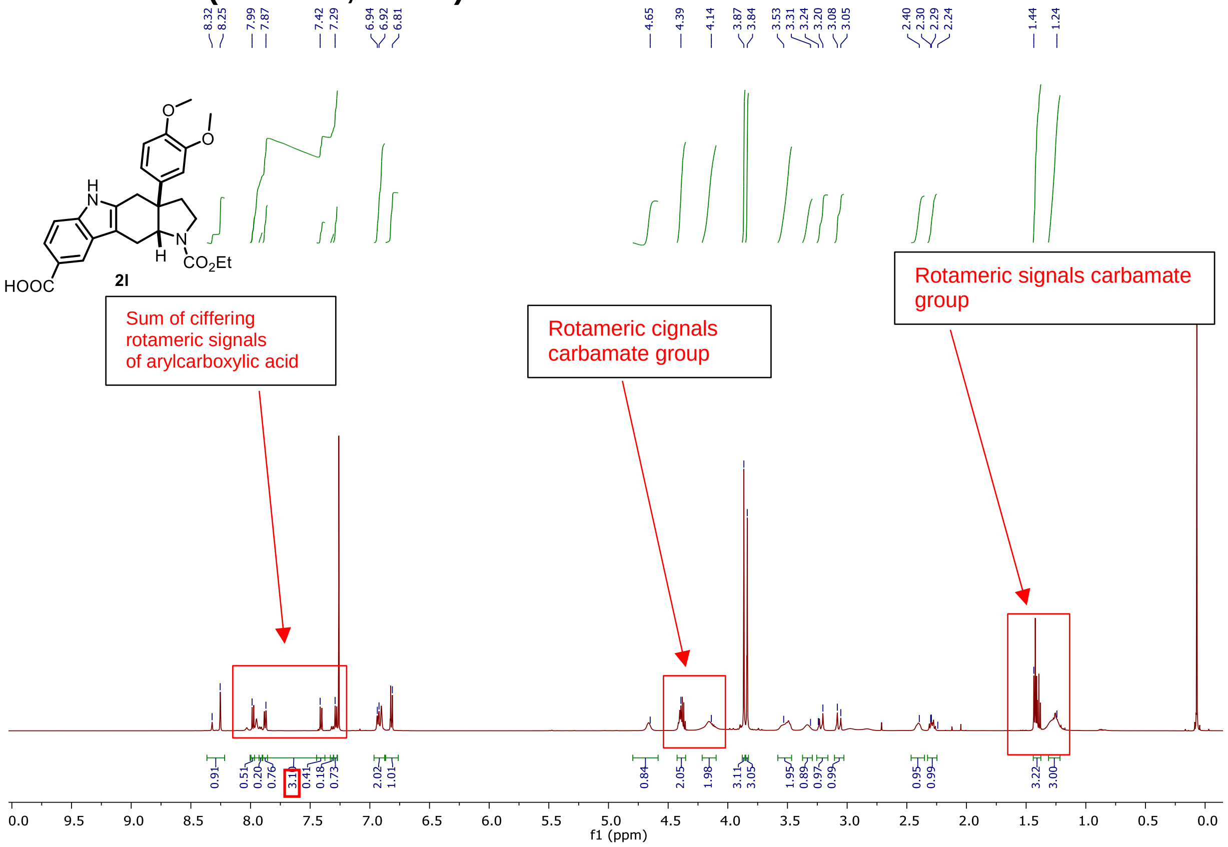

^{13}C-NMR of 2l (151 MHz, $CDCl_3$)

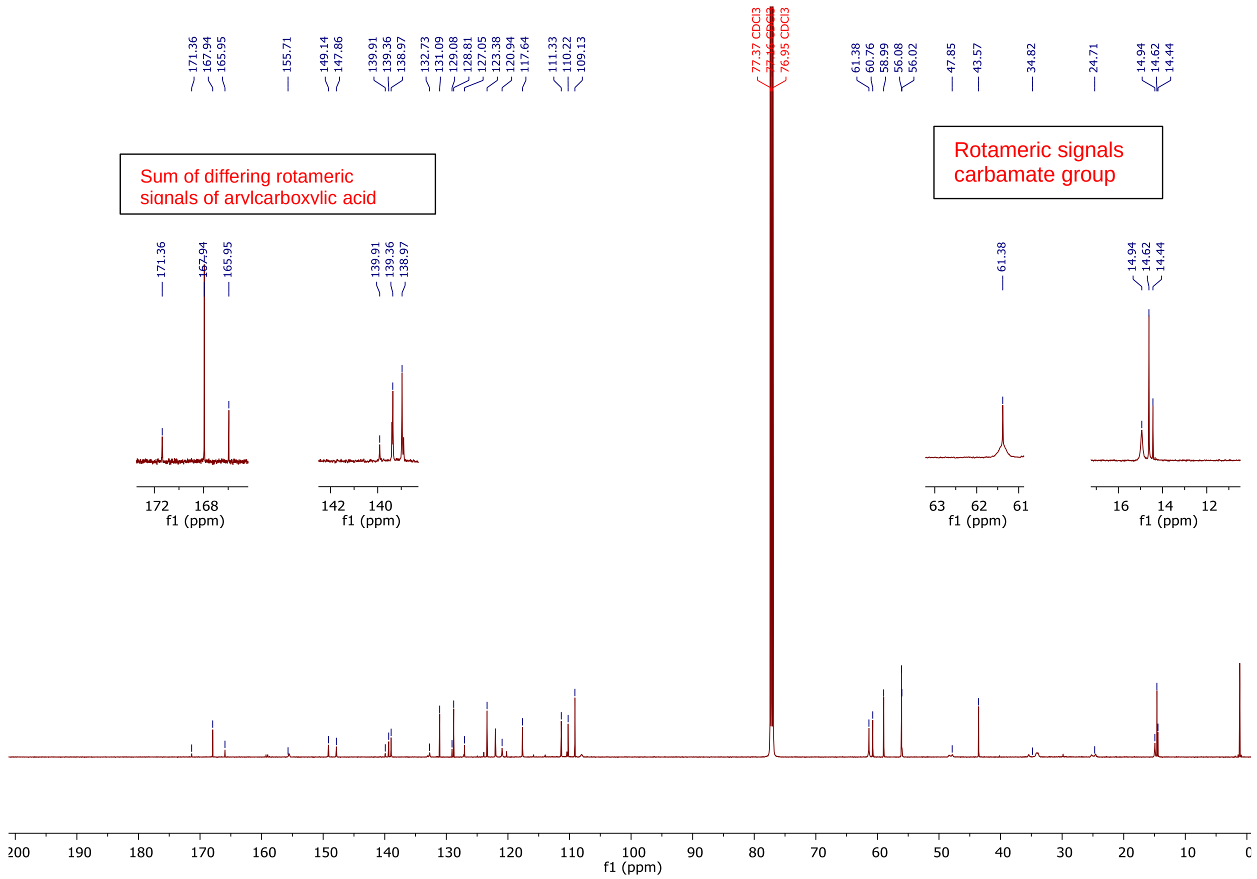

^{1}H-NMR of 2m (500 MHz, $CDCl_3$)

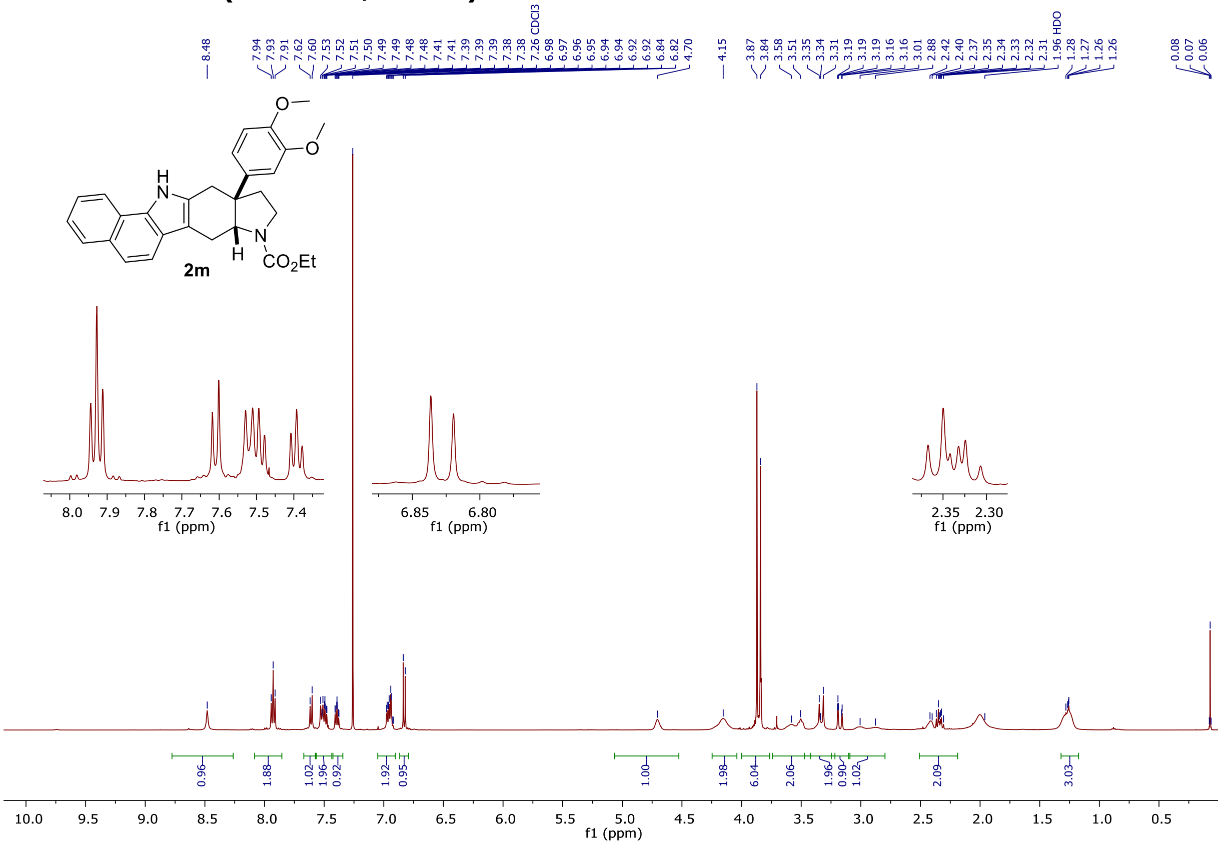

^{13}C-NMR of 2m (126 MHz, $CDCl_3$)

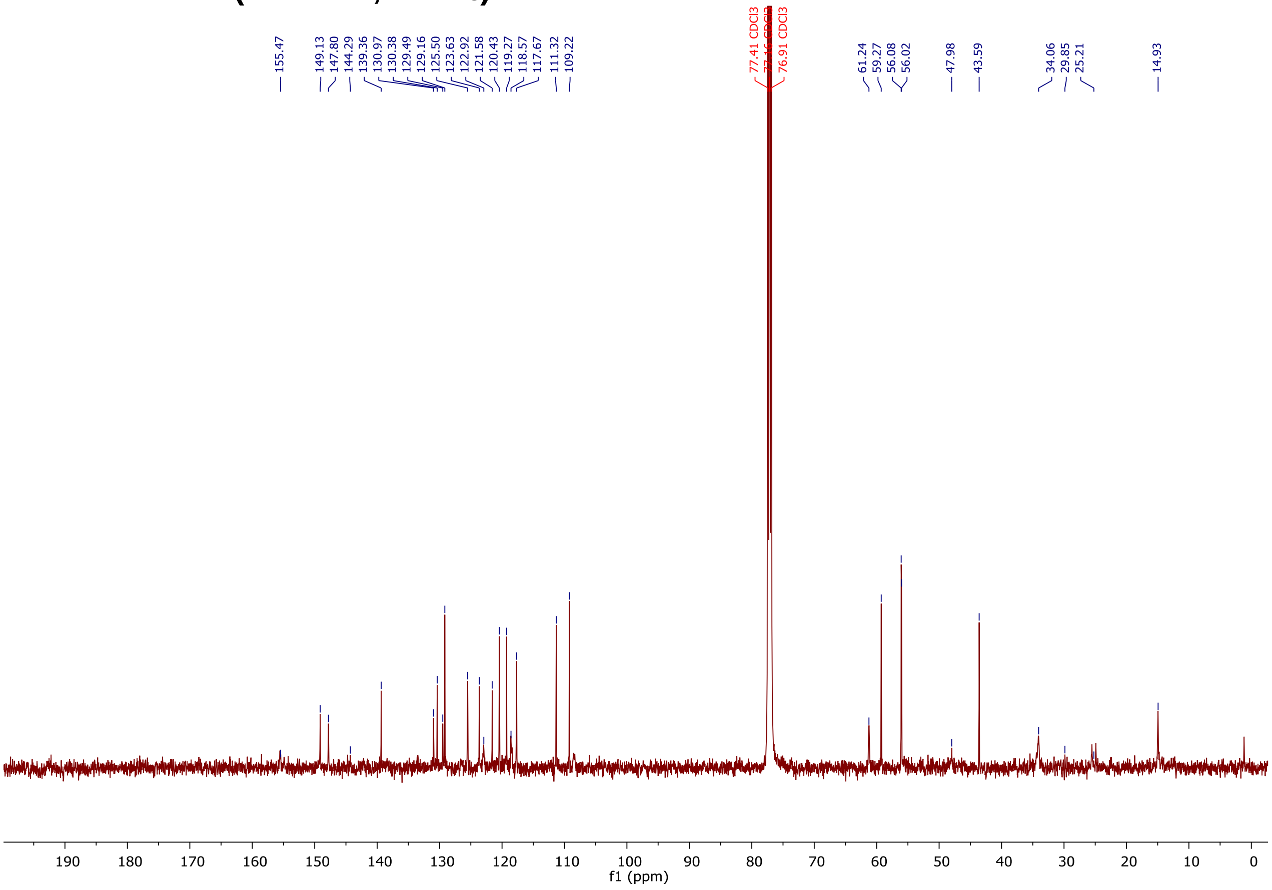

^{1}H-NMR of 3a (700 MHz, $CDCl_3$)

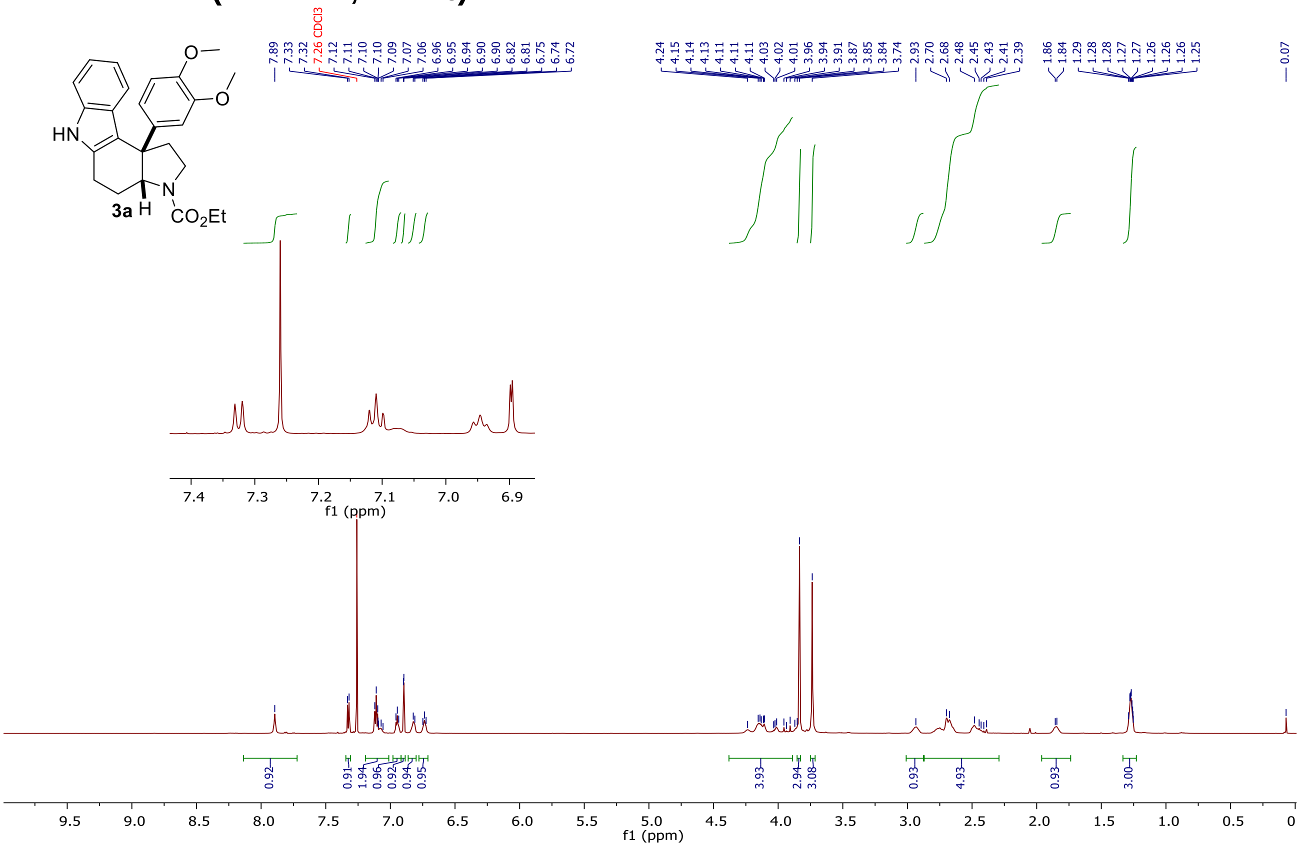

^{13}C-NMR of 3a (176 MHz, $CDCl_3$)

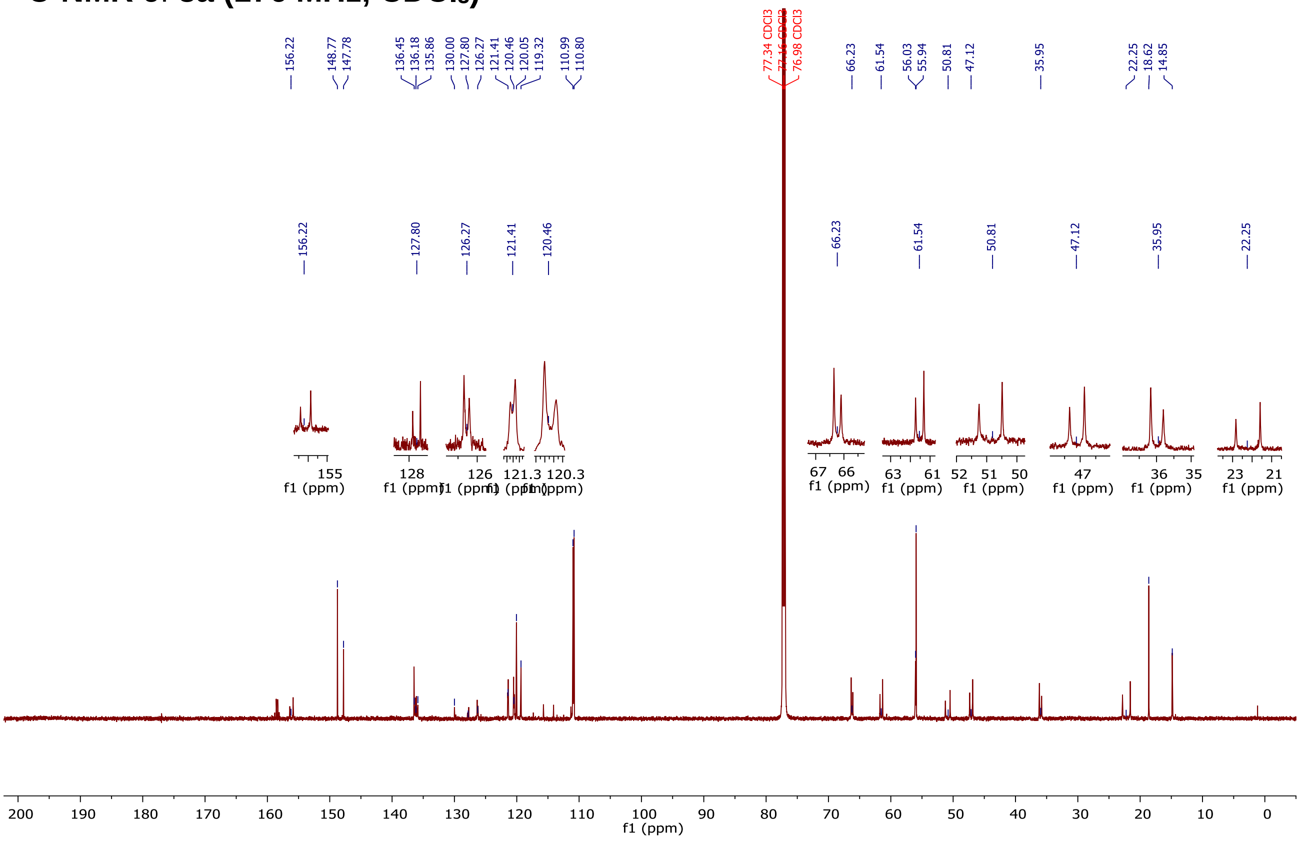

^{1}H-NMR of 3c (700 MHz, $CDCl_3$)

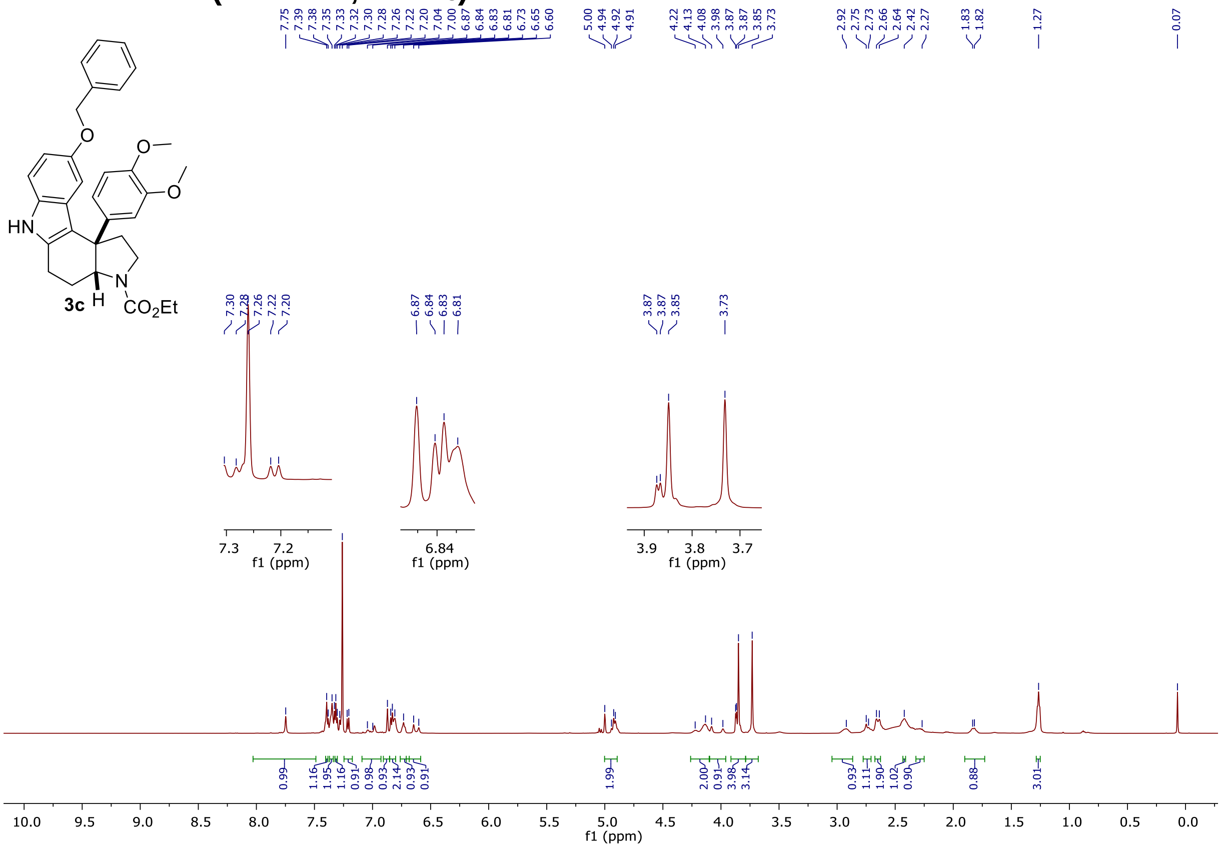

^{13}C-NMR of 3c (151 MHz, $CDCl_3$)

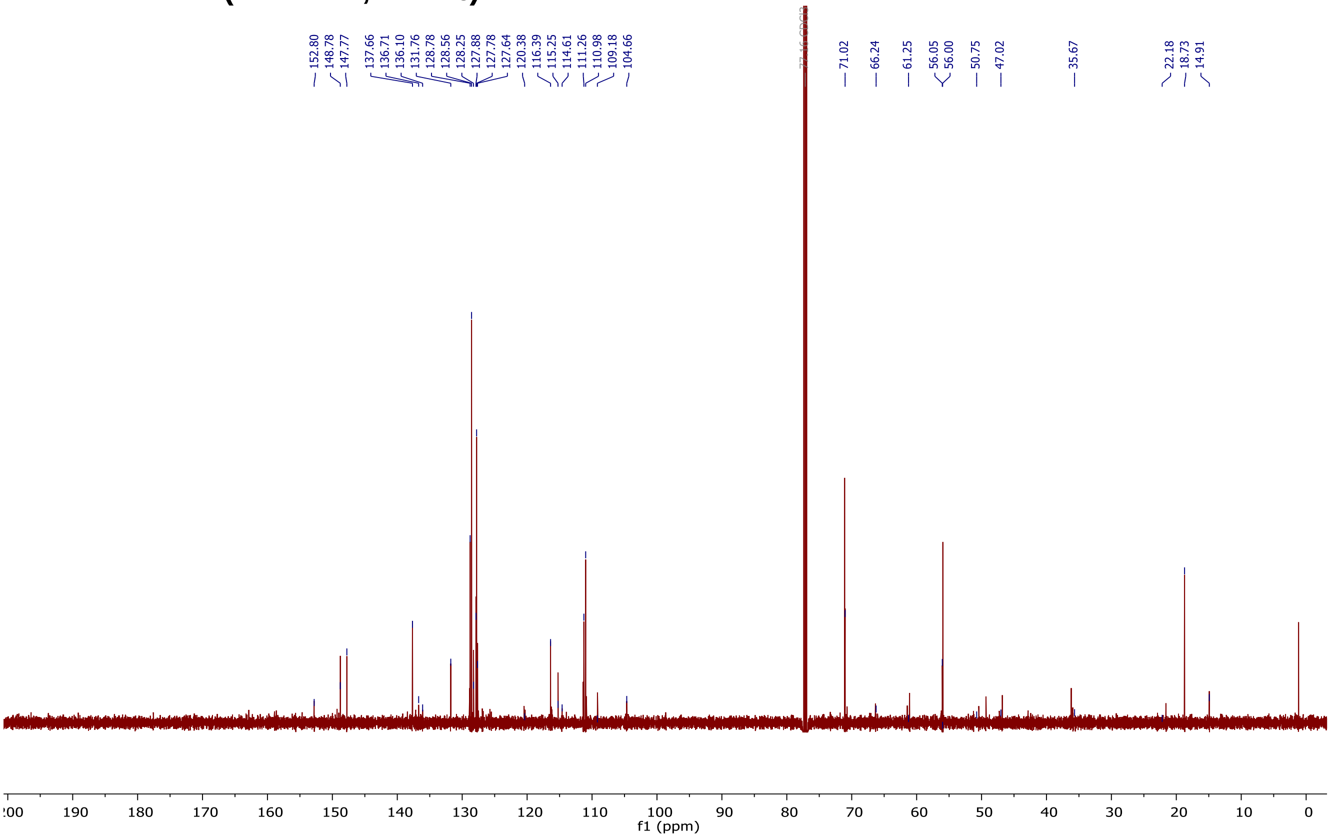

^{1}H-NMR of 3f (700 MHz, $CDCl_3$)

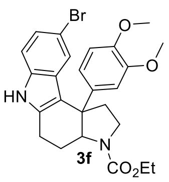

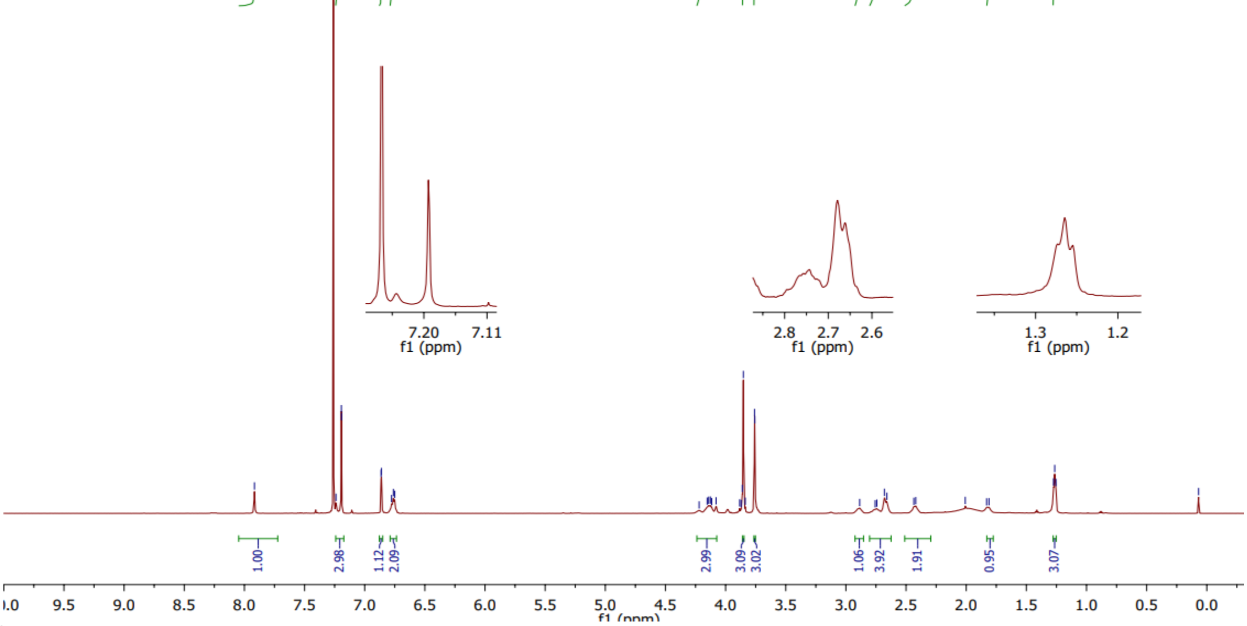

^{13}C-NMR of 3f (151 MHz, $CDCl_3$)

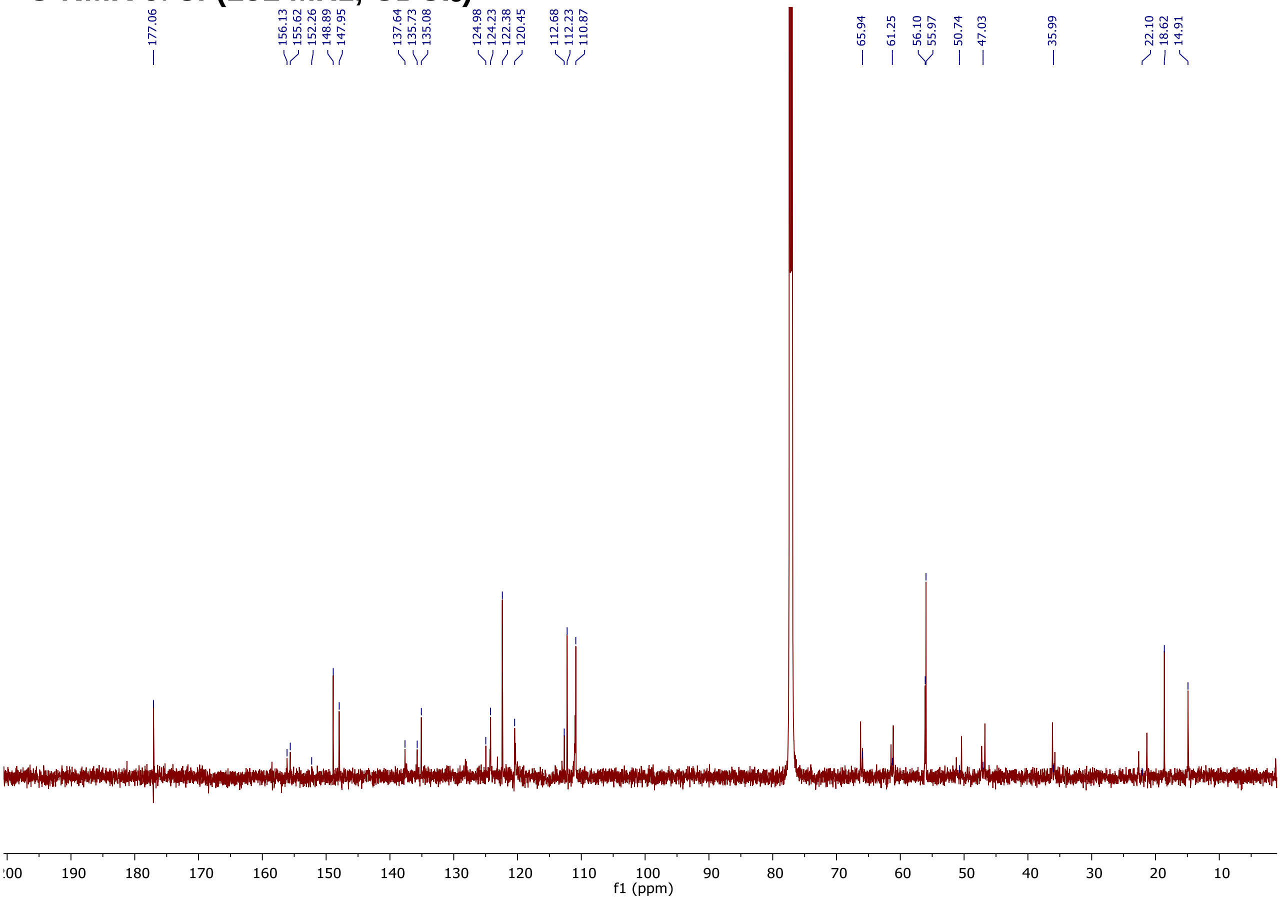

^{1}H-NMR of 3g (700 MHz, CDCl3)

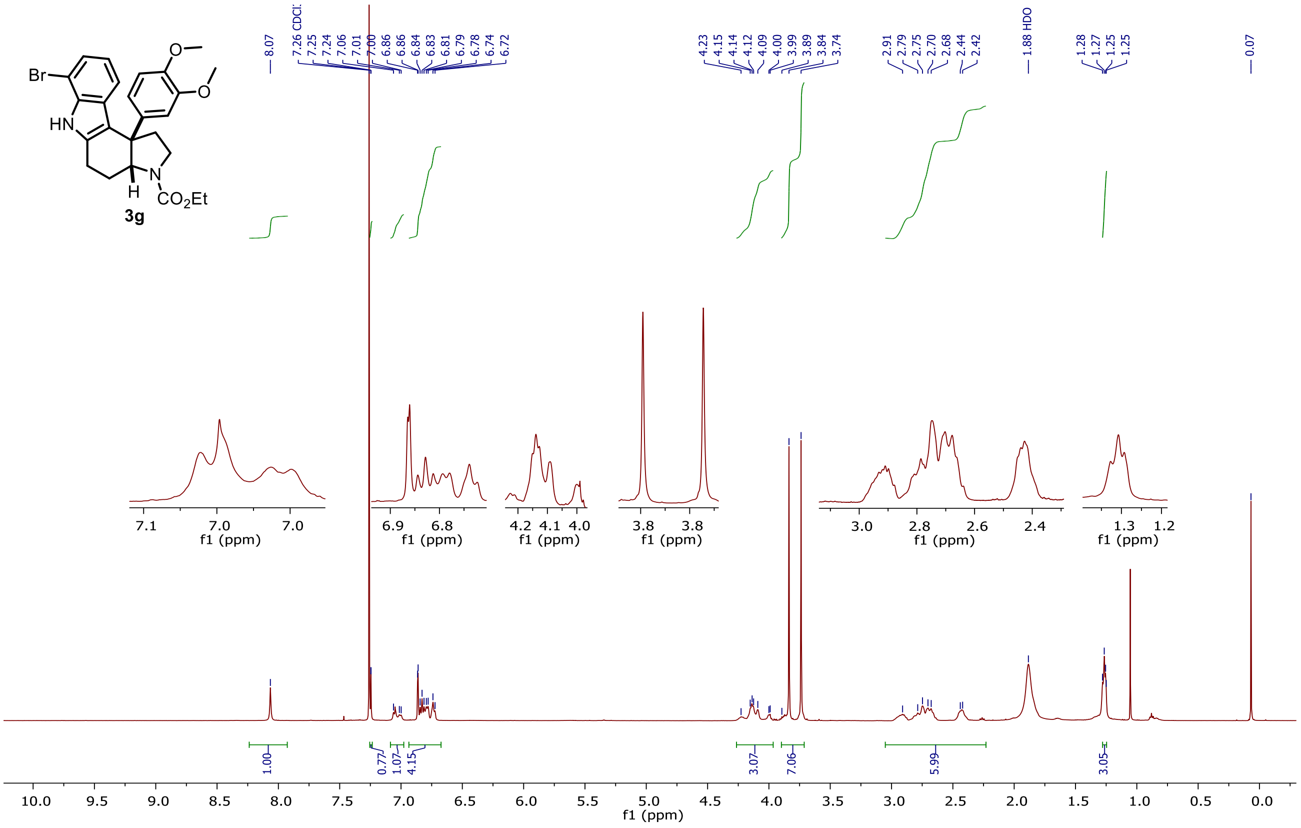

^{13}C-NMR of 3g (126 MHz, CDCl3)

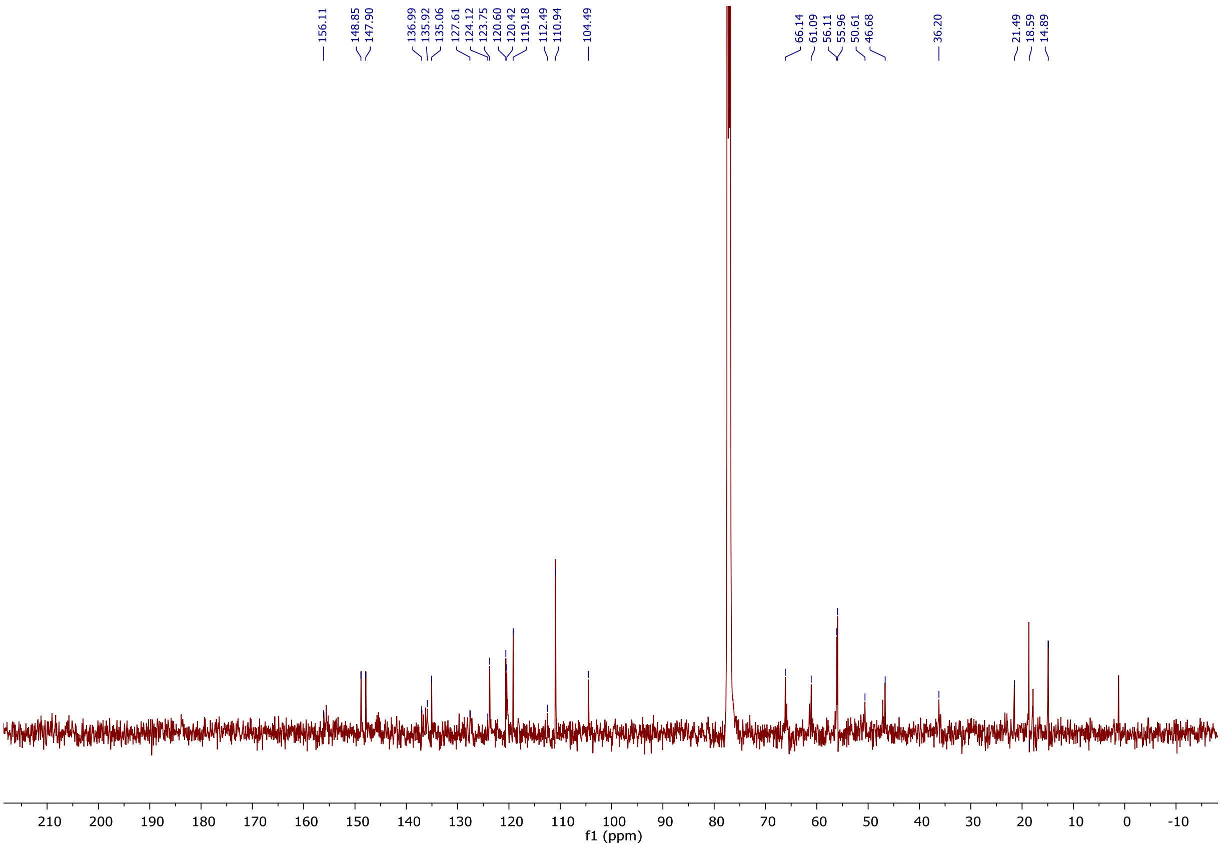

HSQC-NMR of **3g (CDCl$_3$):** Hidden methylene signal under residual water.

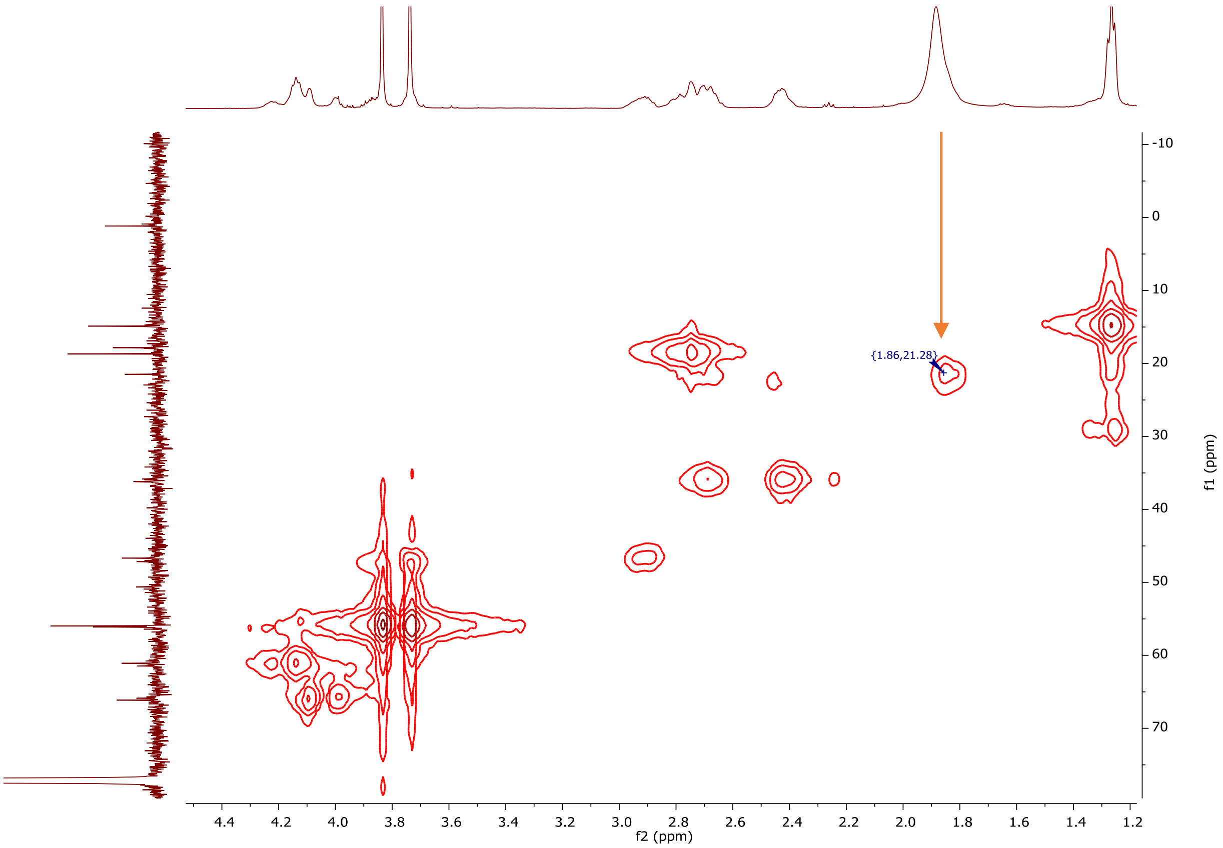

^{1}H-NMR of 3h (700 MHz, $CDCl_3$)

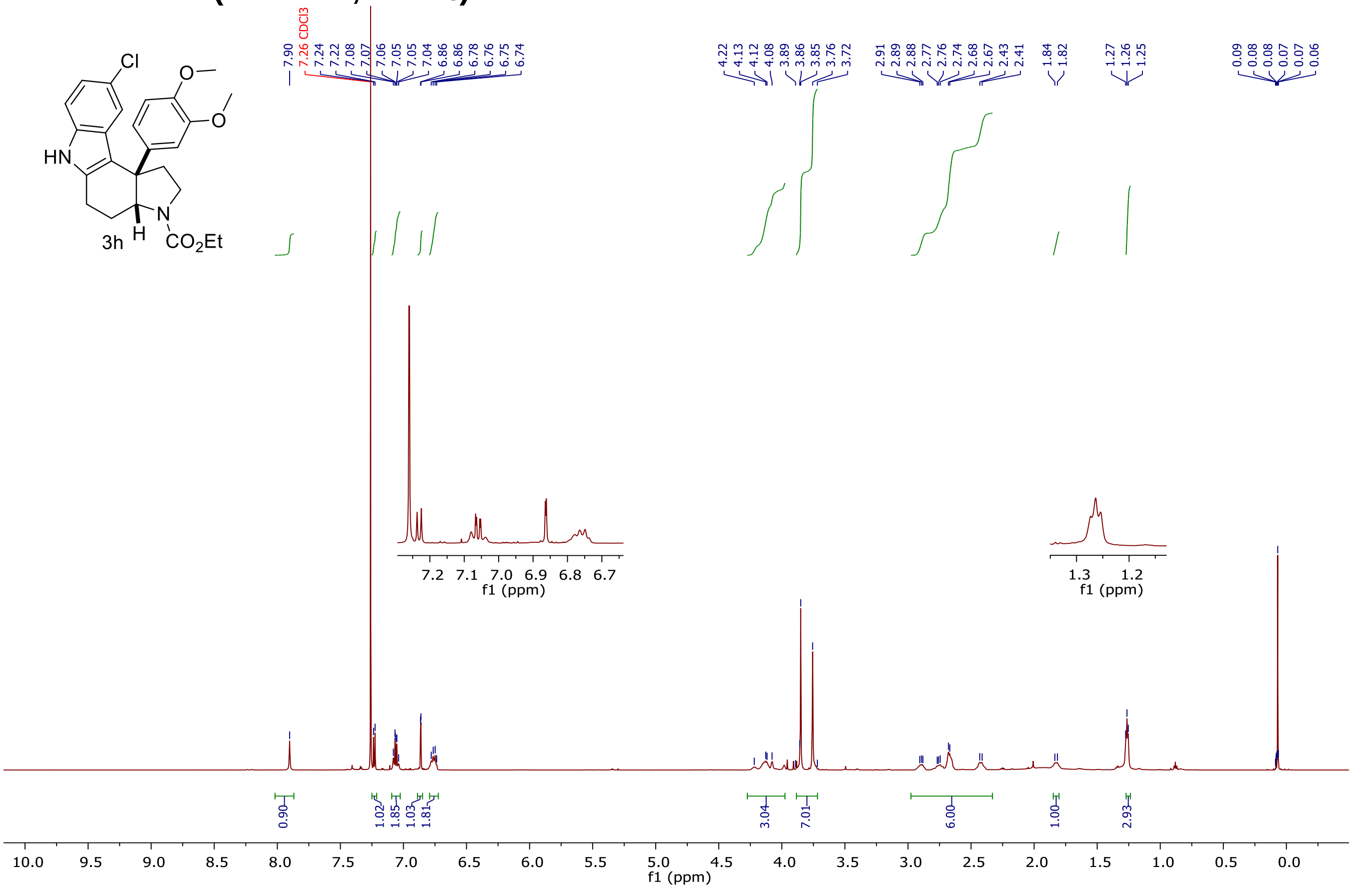

^{13}C-NMR of 3h (176 MHz, $CDCl_3$)

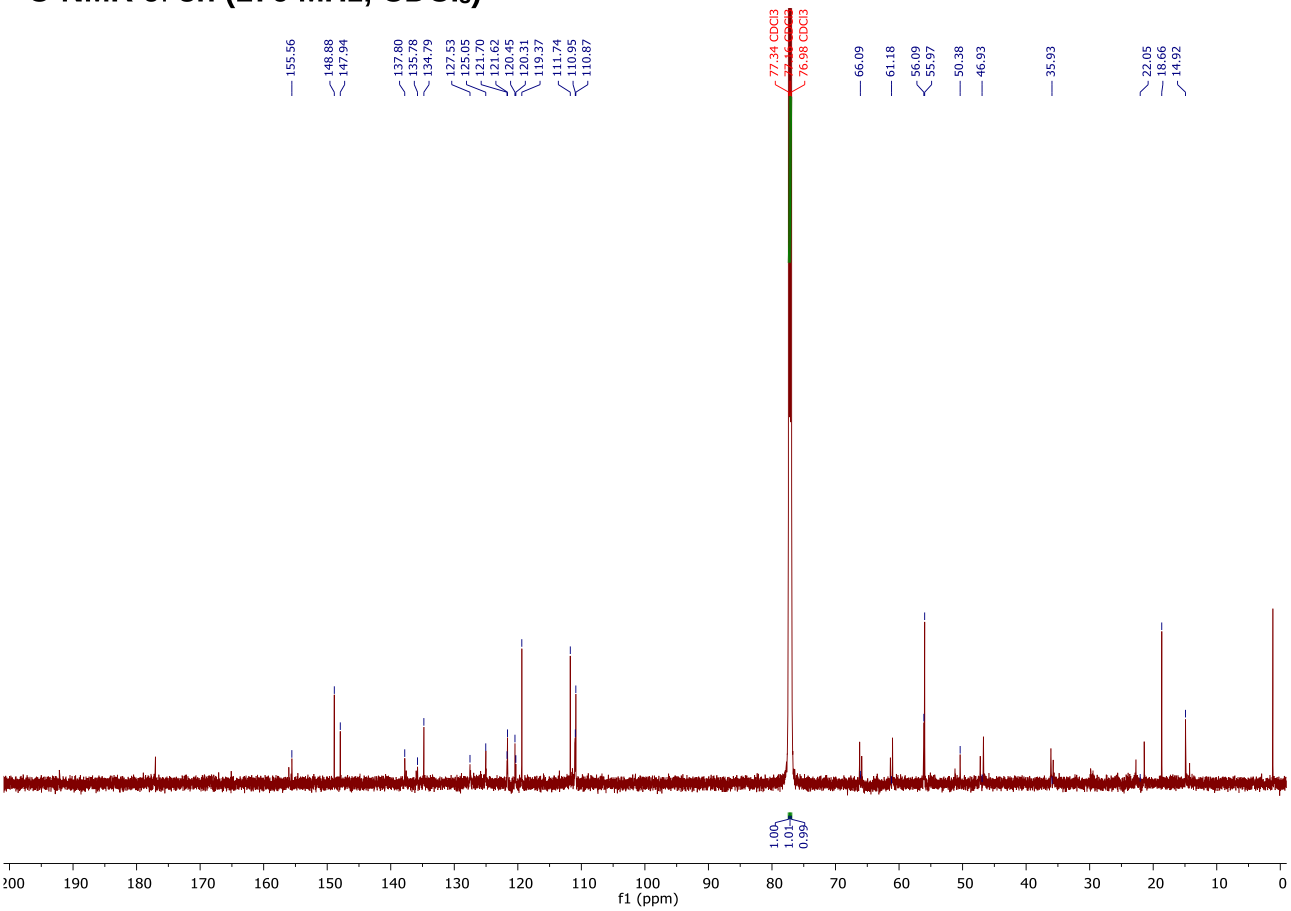

^{1}H-NMR of 3i (700 MHz, $CDCl_3$)

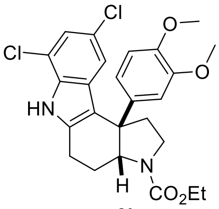

3i

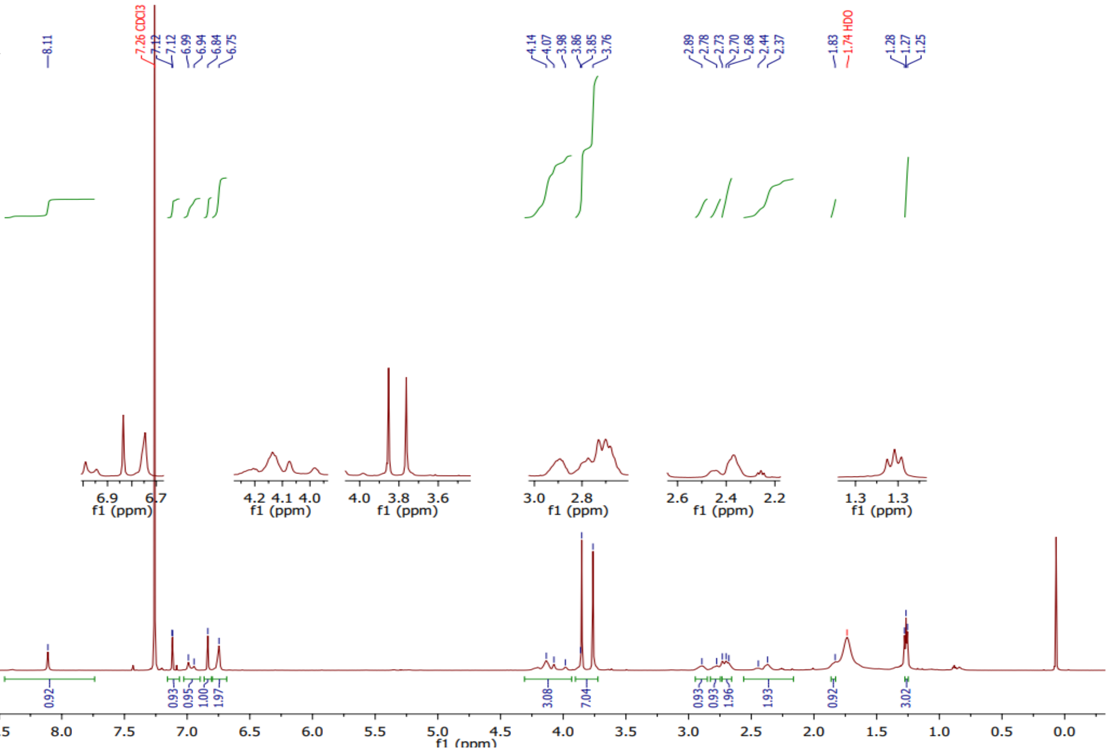

^{1}H-NMR of 3i (176 MHz, $CDCl_3$)

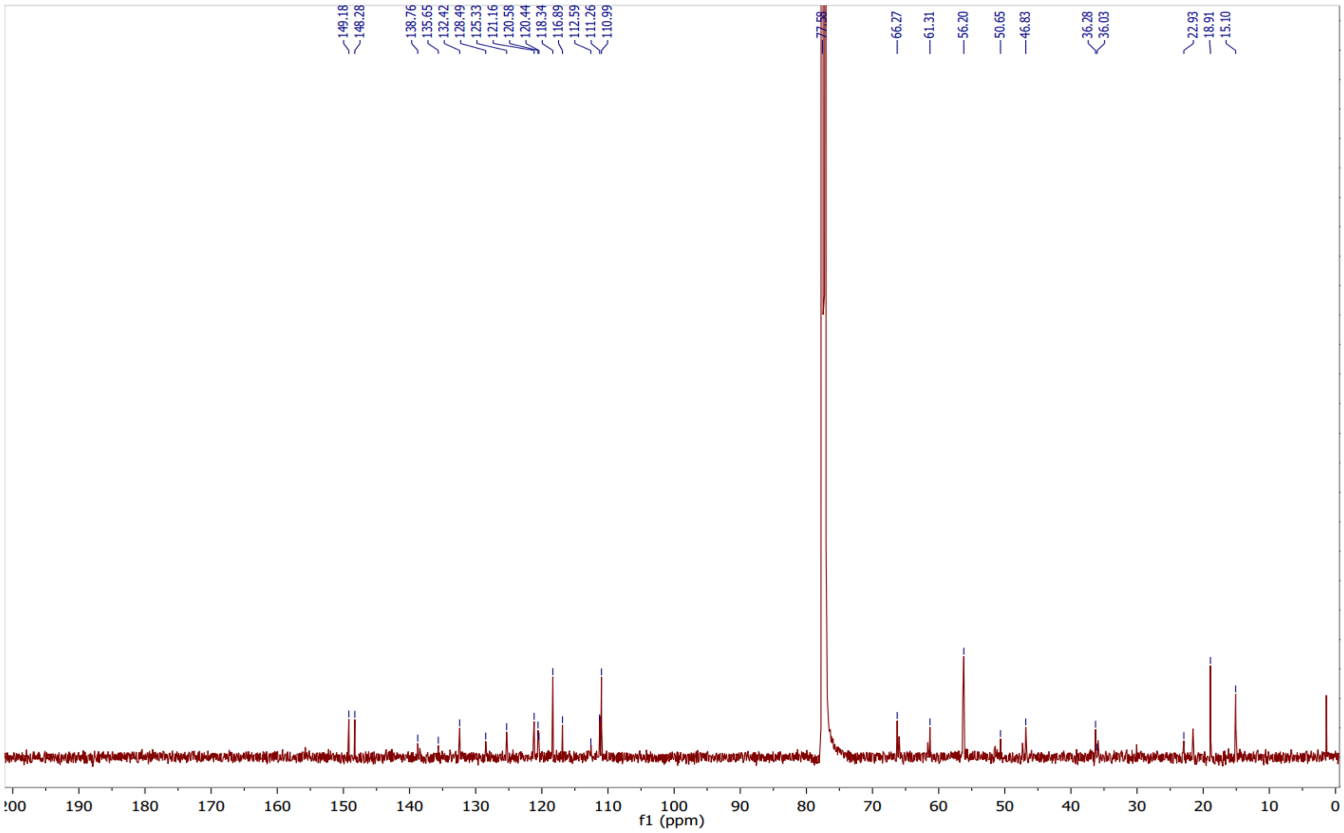

^{1}H-NMR of 3m (600 MHz, $CDCl_3$)

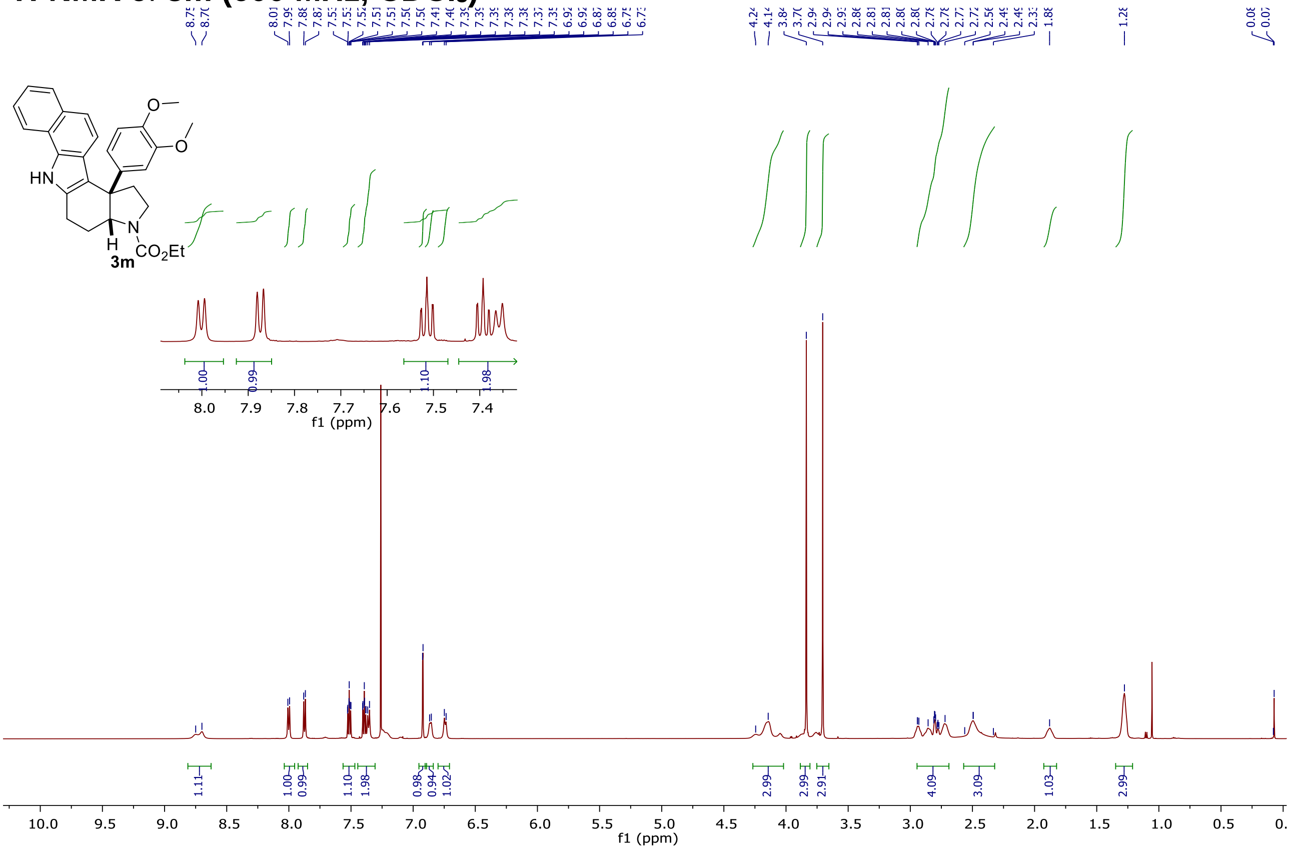

^{13}C-NMR of 3m (151 MHz, $CDCl_3$)

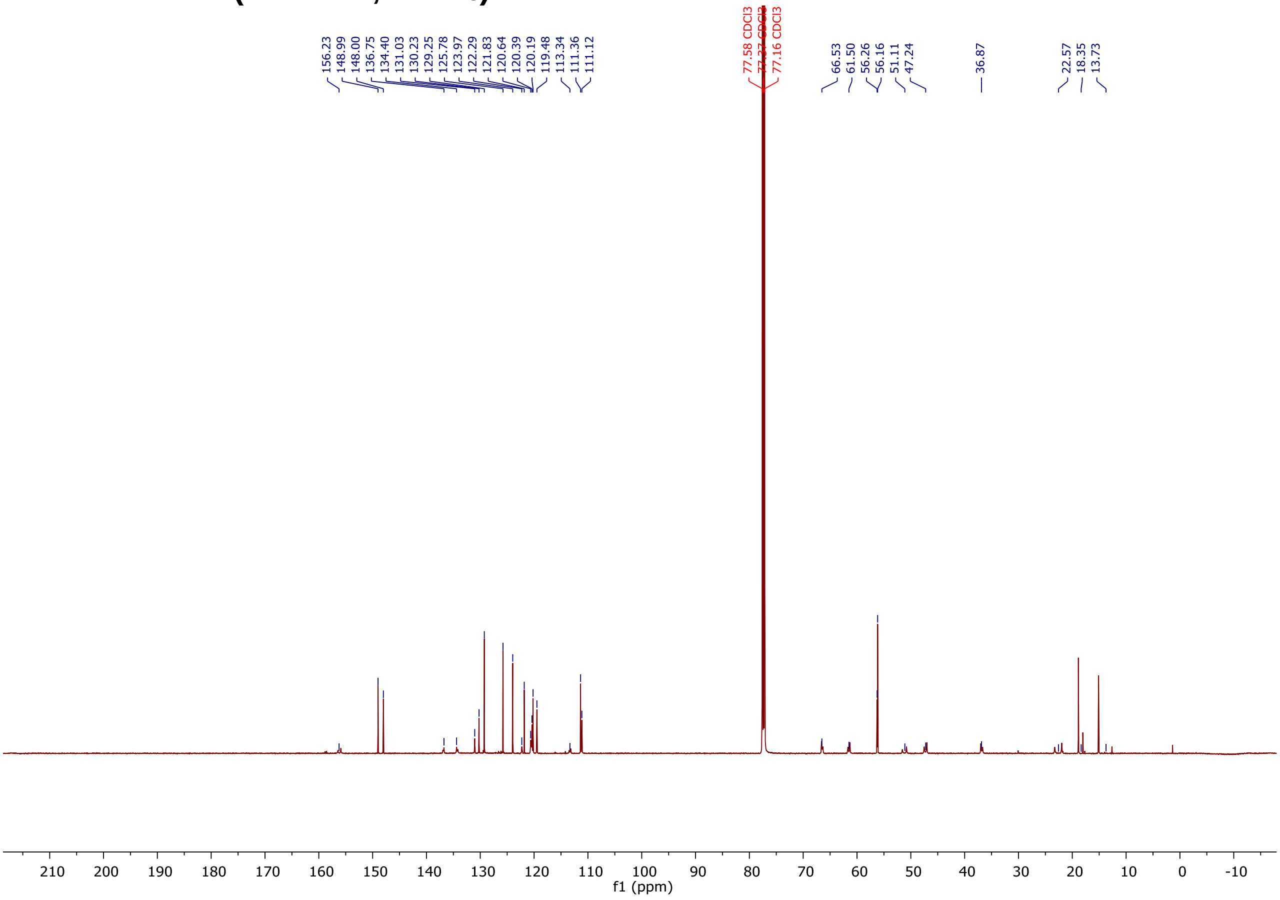

^{1}H-NMR of 3n (700 MHz, $CDCl_3$)

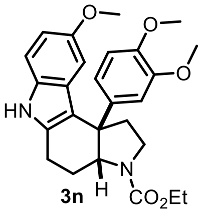

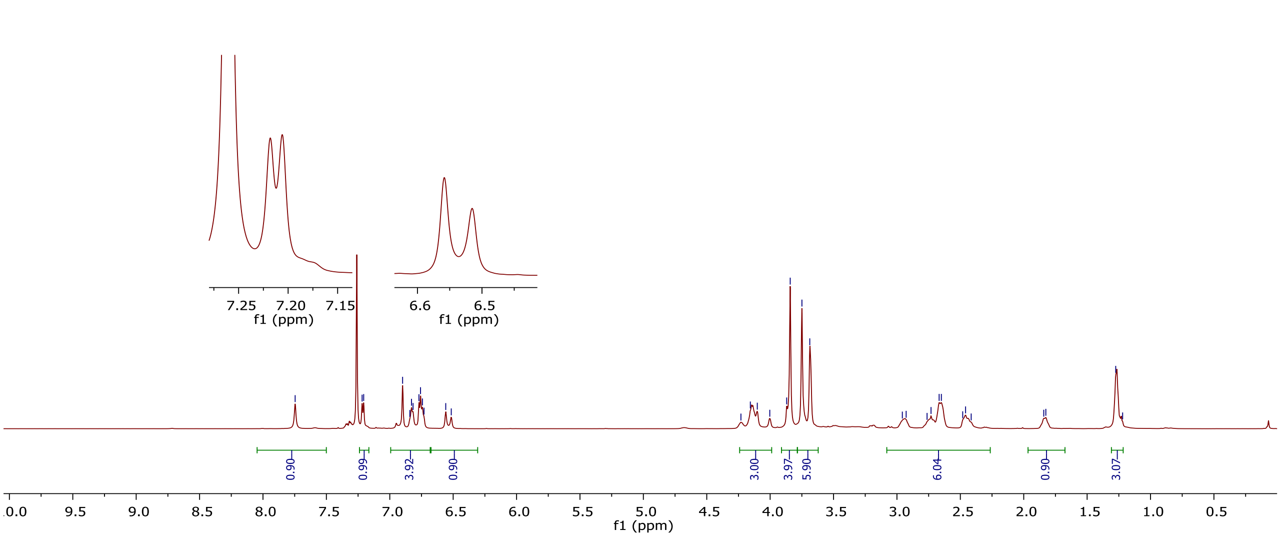

^{1}H-NMR of 3n (176 MHz, $CDCl_3$)

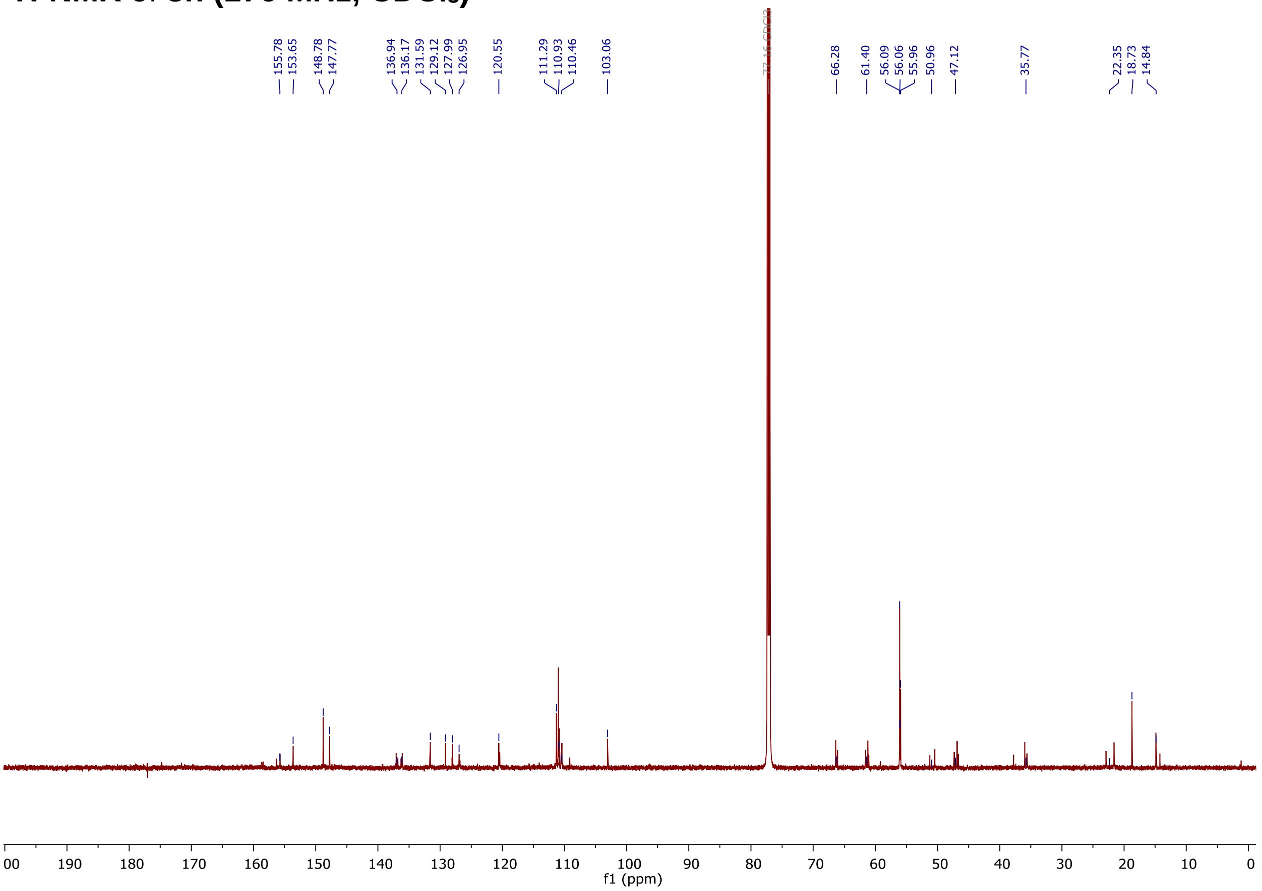

^{1}H-NMR of **5** (500 MHz, $CDCl_3$)

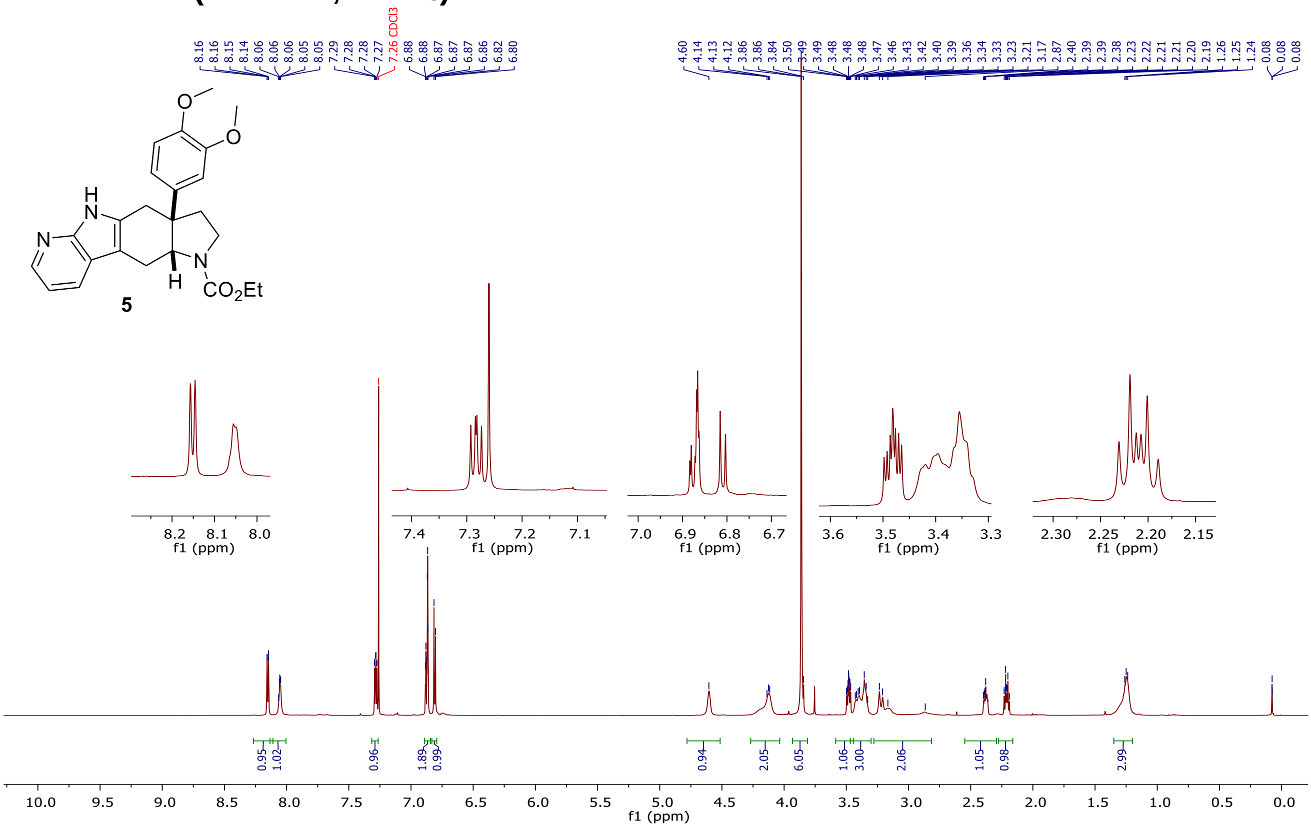

^{13}C-NMR of **5** (500 MHz, $CDCl_3$)

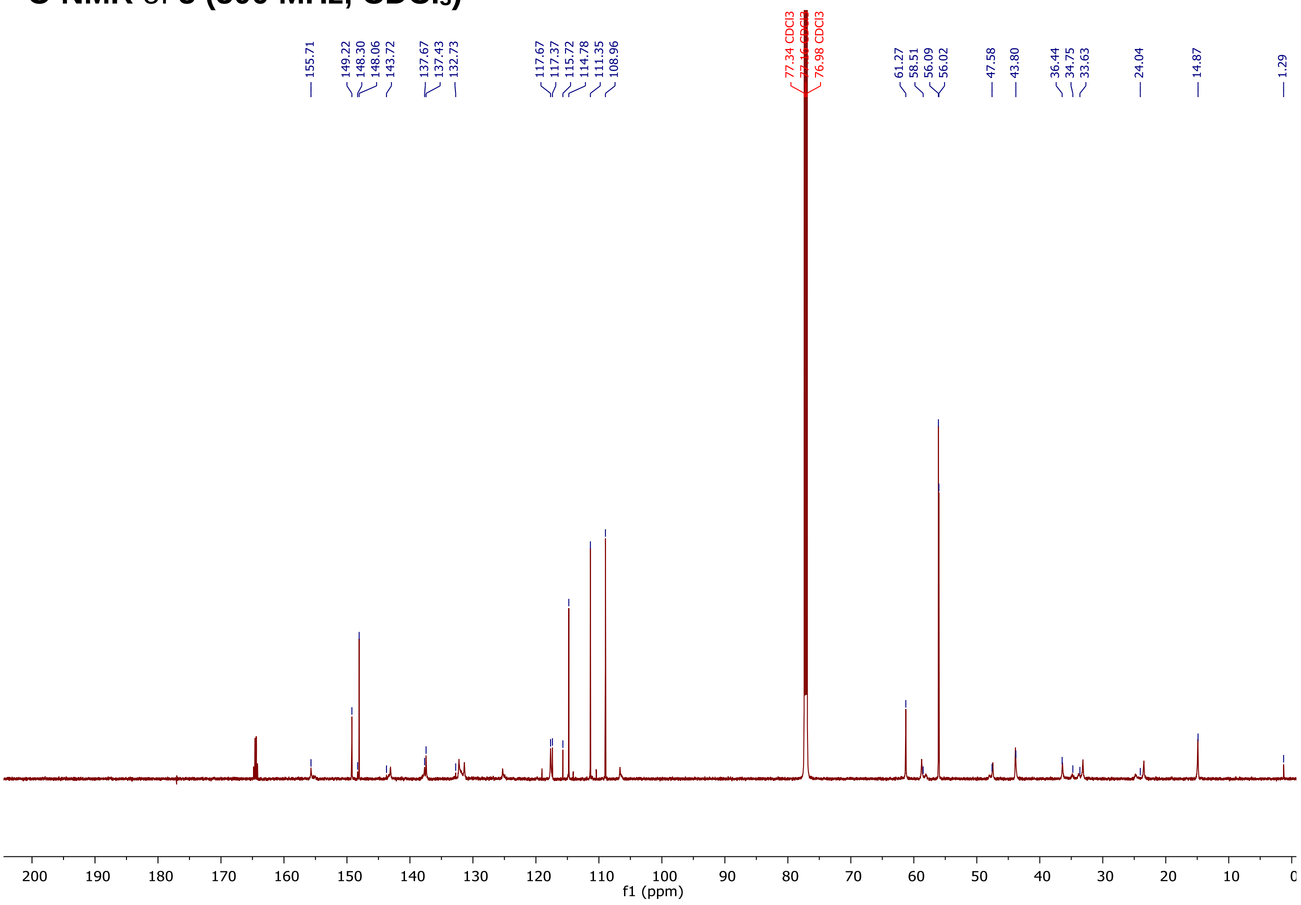

^{1}H-NMR of 7a (700 MHz, $CDCl_3$)

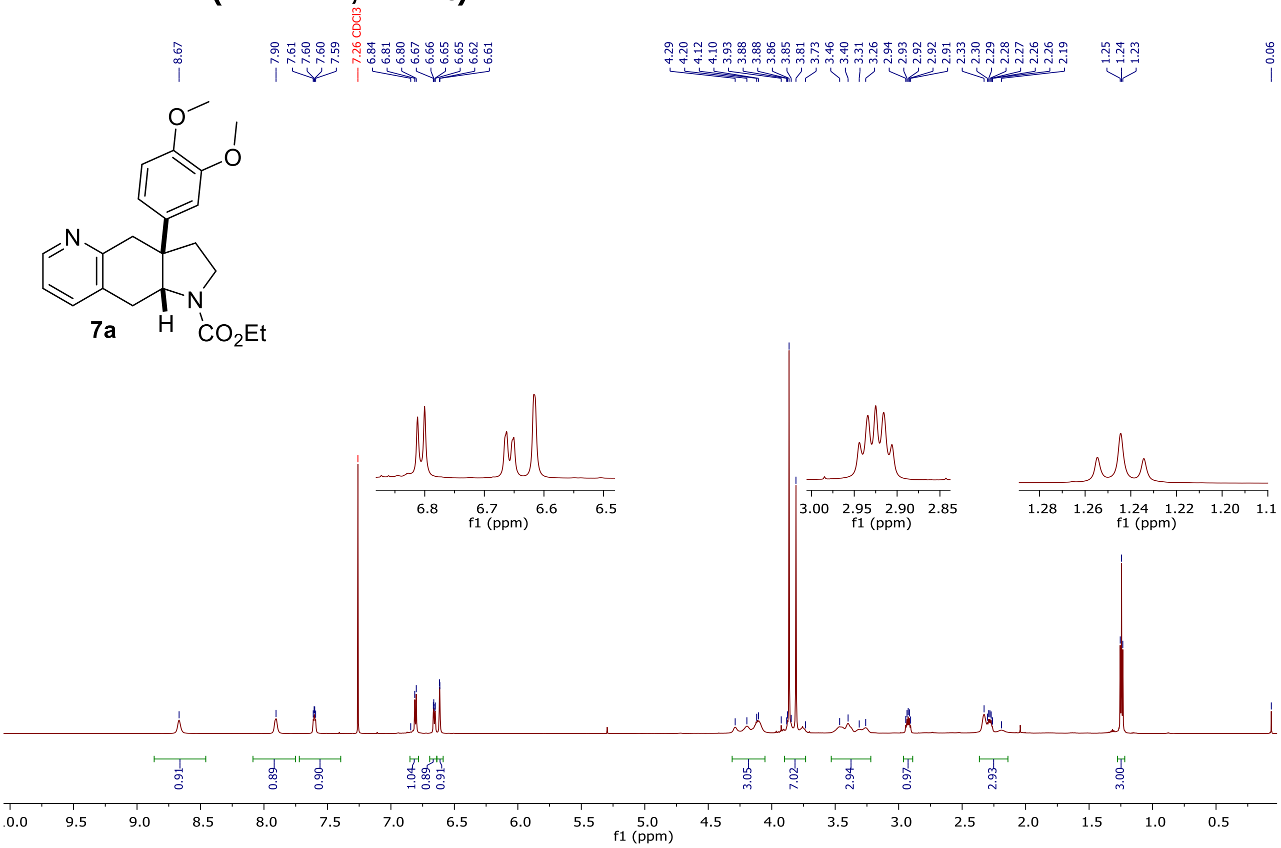

^{13}C-NMR of 7a (176 MHz, $CDCl_3$)

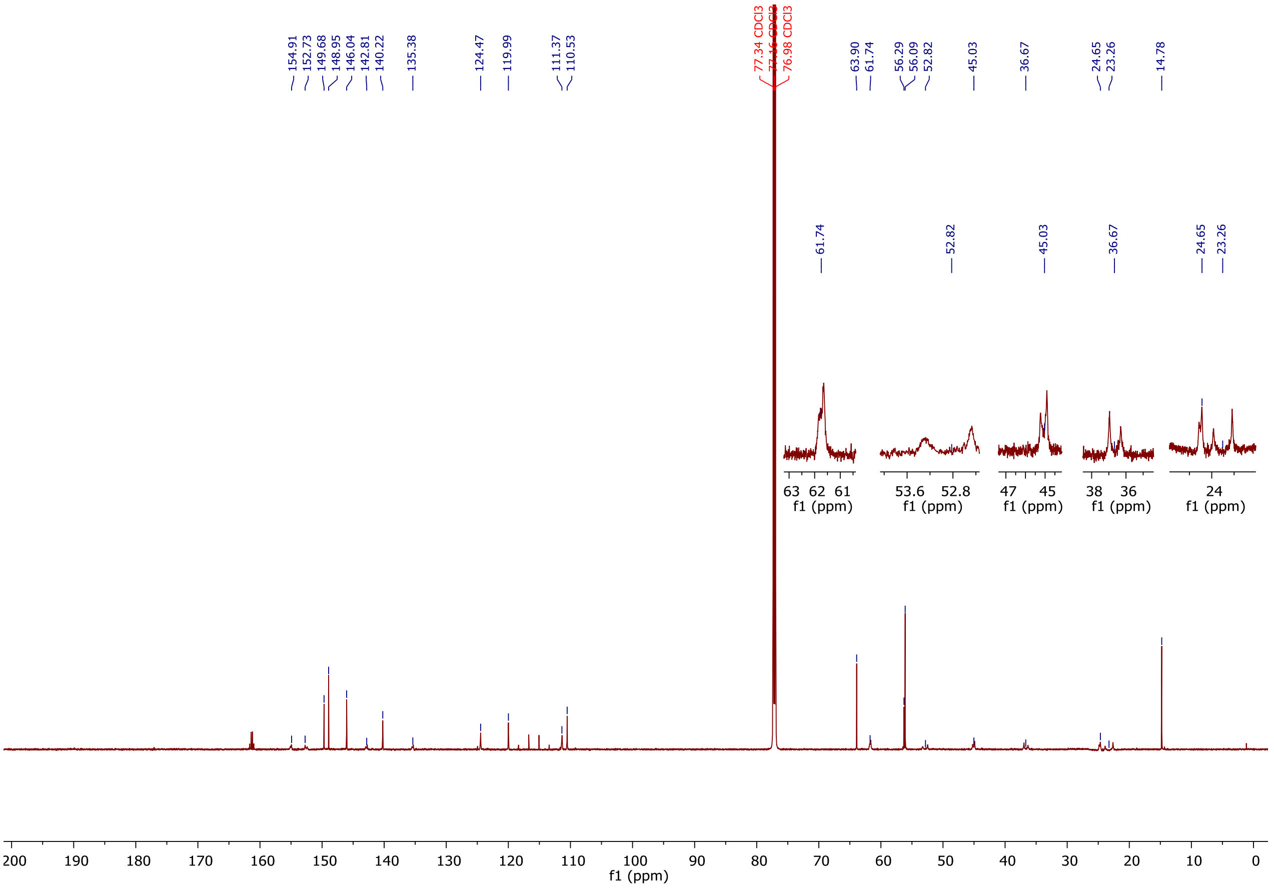

^{1}H-NMR of 7b (700 MHz, CDCl$_3$)

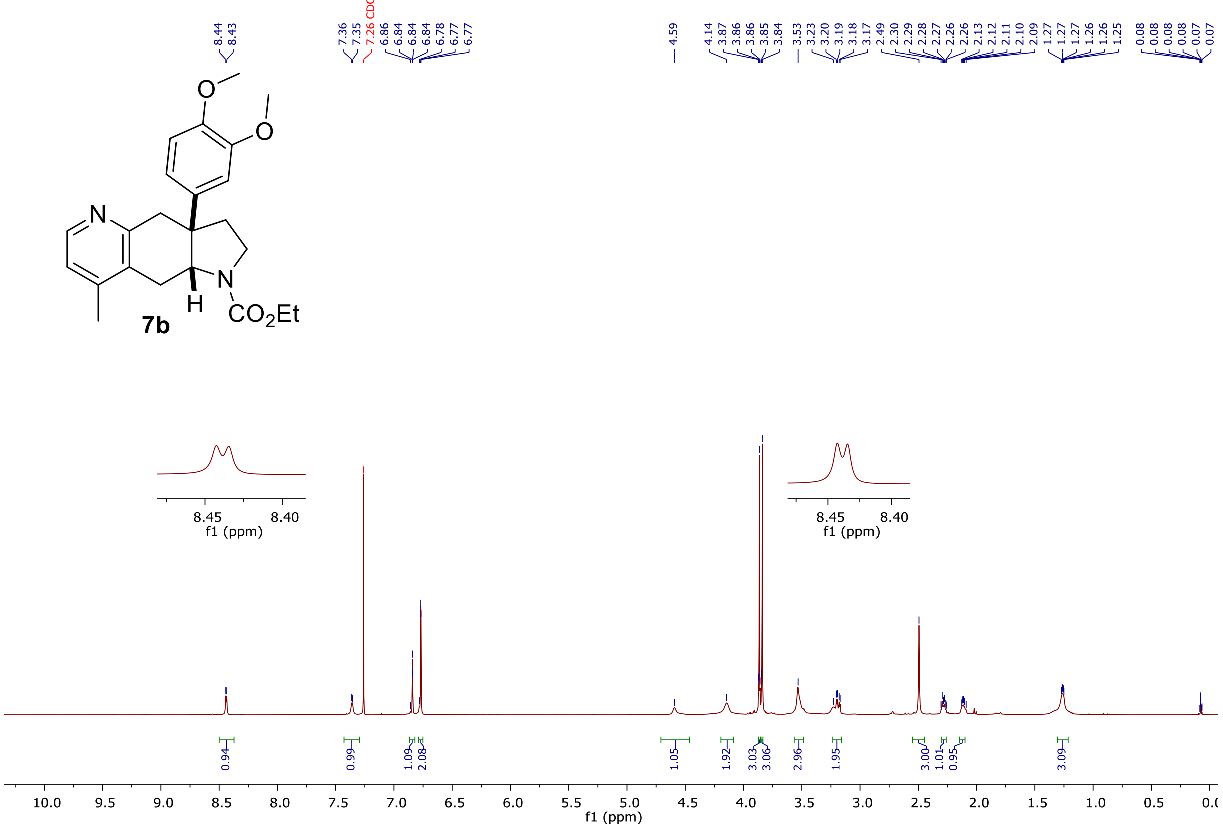

^{13}C-NMR of 7b (176 MHz, CDCl$_3$)

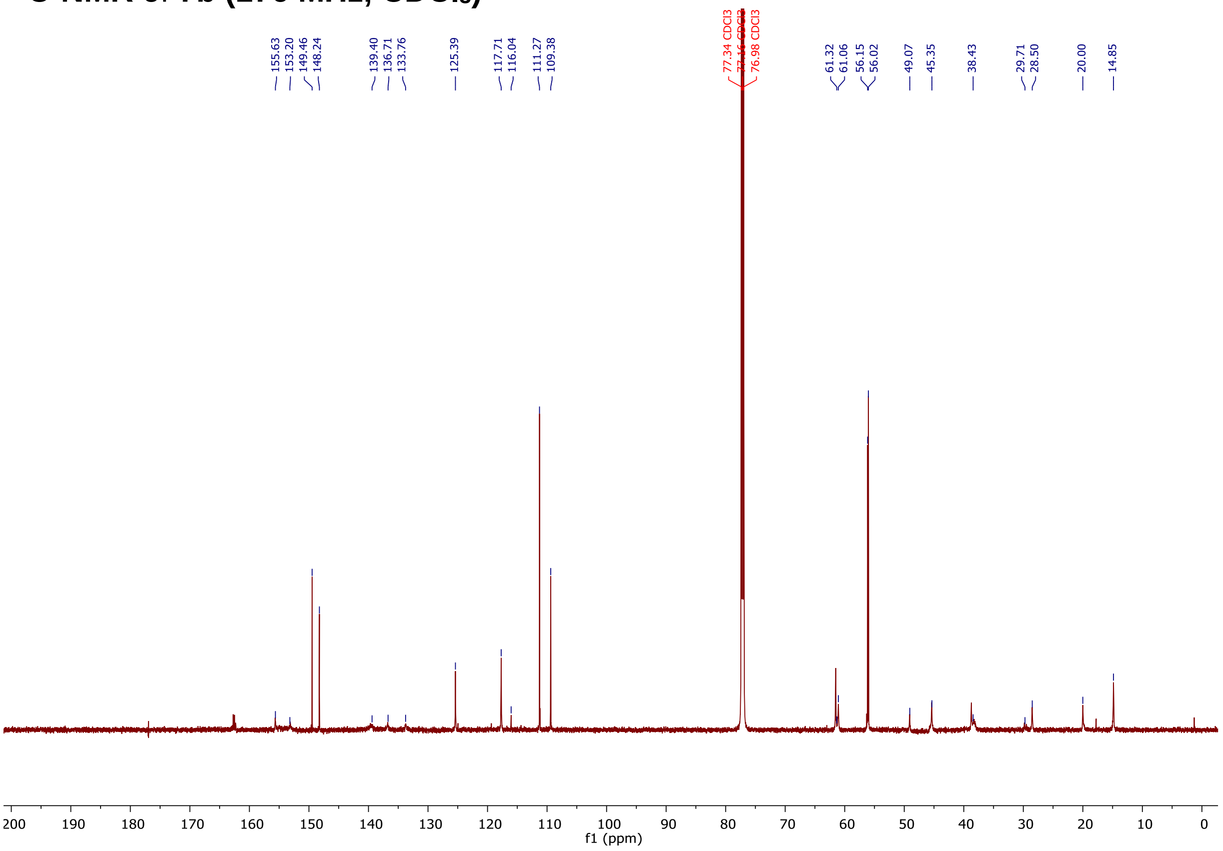

^{1}H-NMR of **7c** (700 MHz, $CDCl_3$)

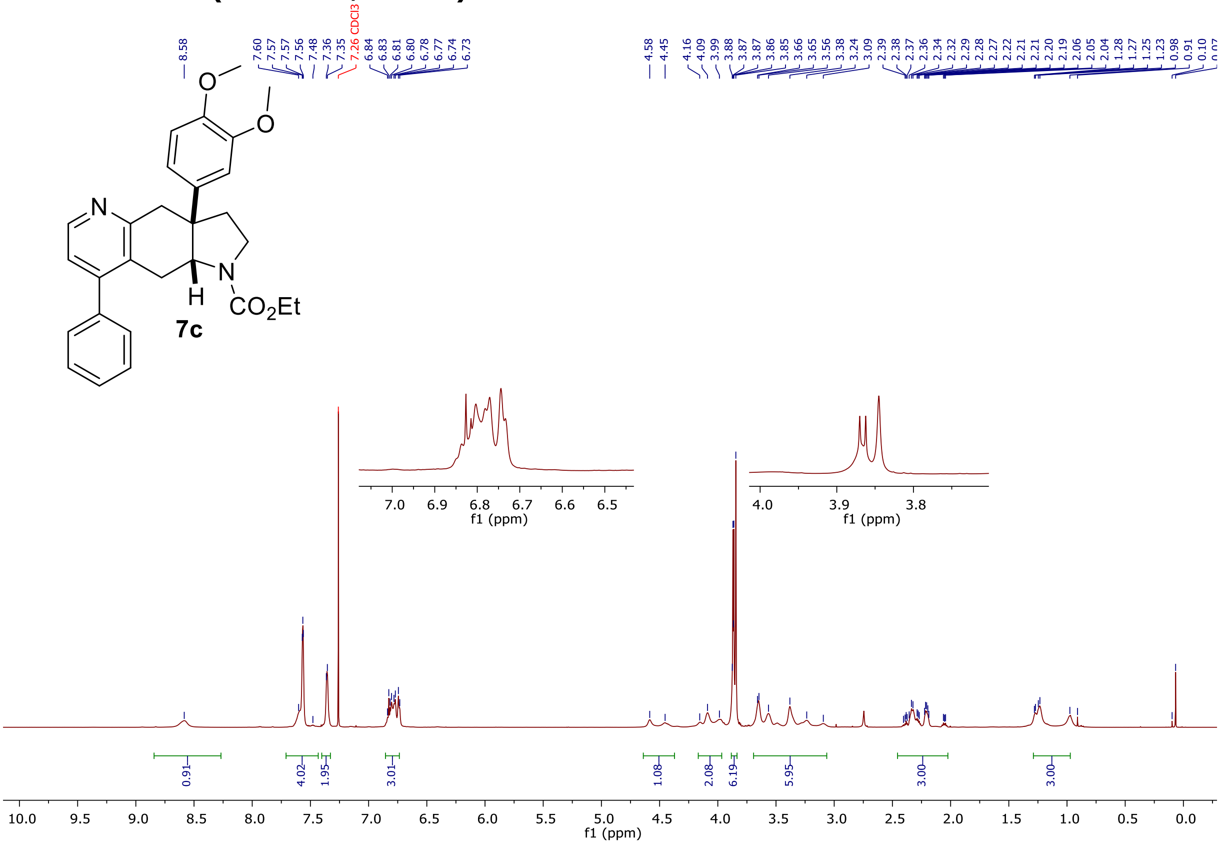

^{13}C-NMR of **7c** (176 MHz, $CDCl_3$)

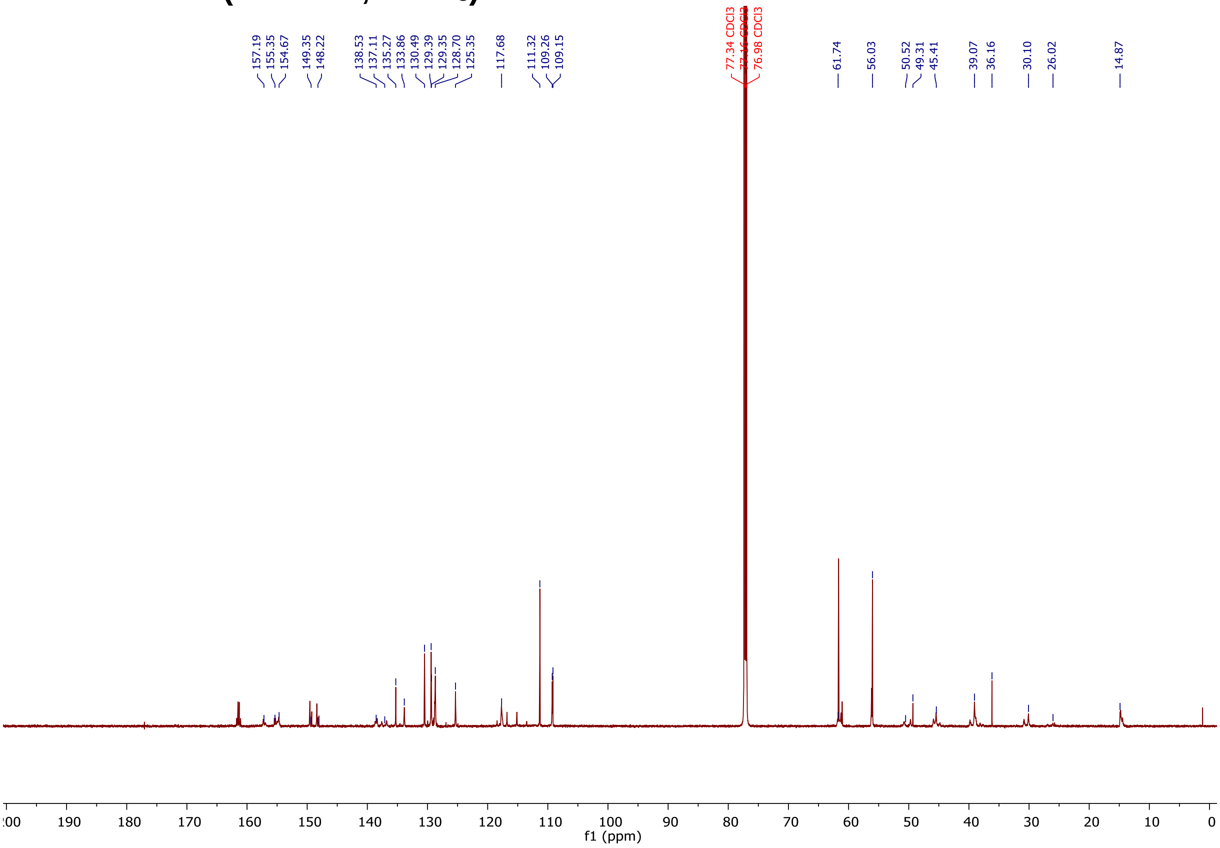

¹H-NMR of **9** (700 MHz, $CDCl_3$)

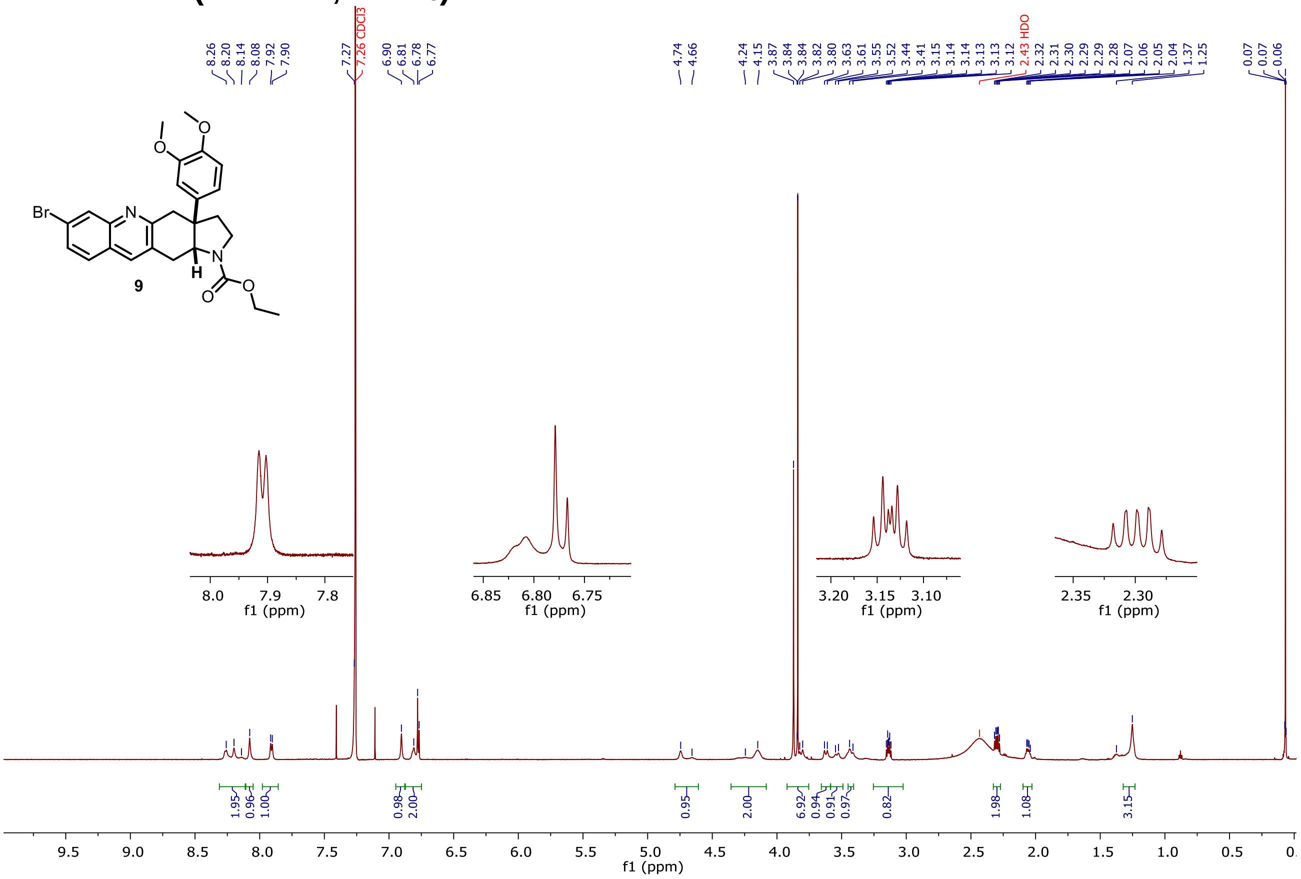

¹³C-NMR of **9** (176 MHz, $CDCl_3$)

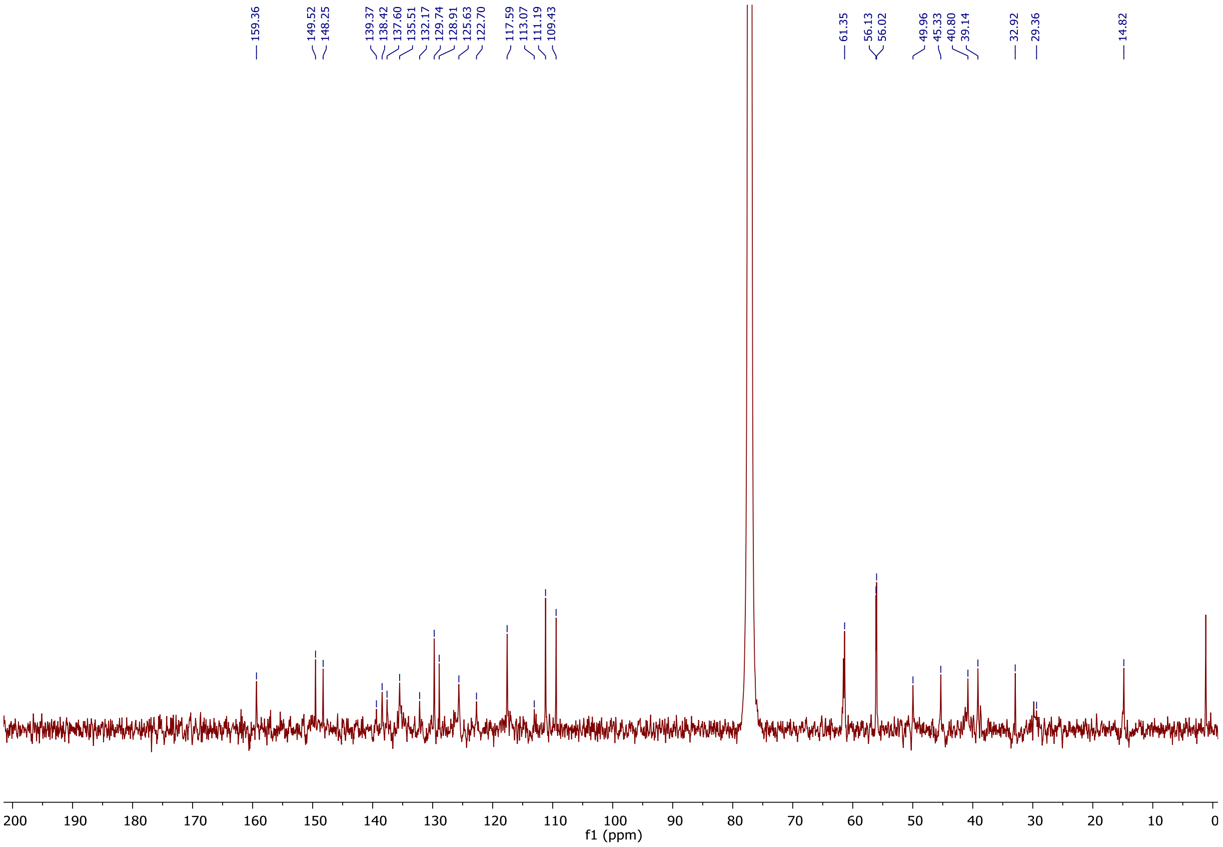

^{1}H-NMR of 11 (700 MHz, $CDCl_3$)

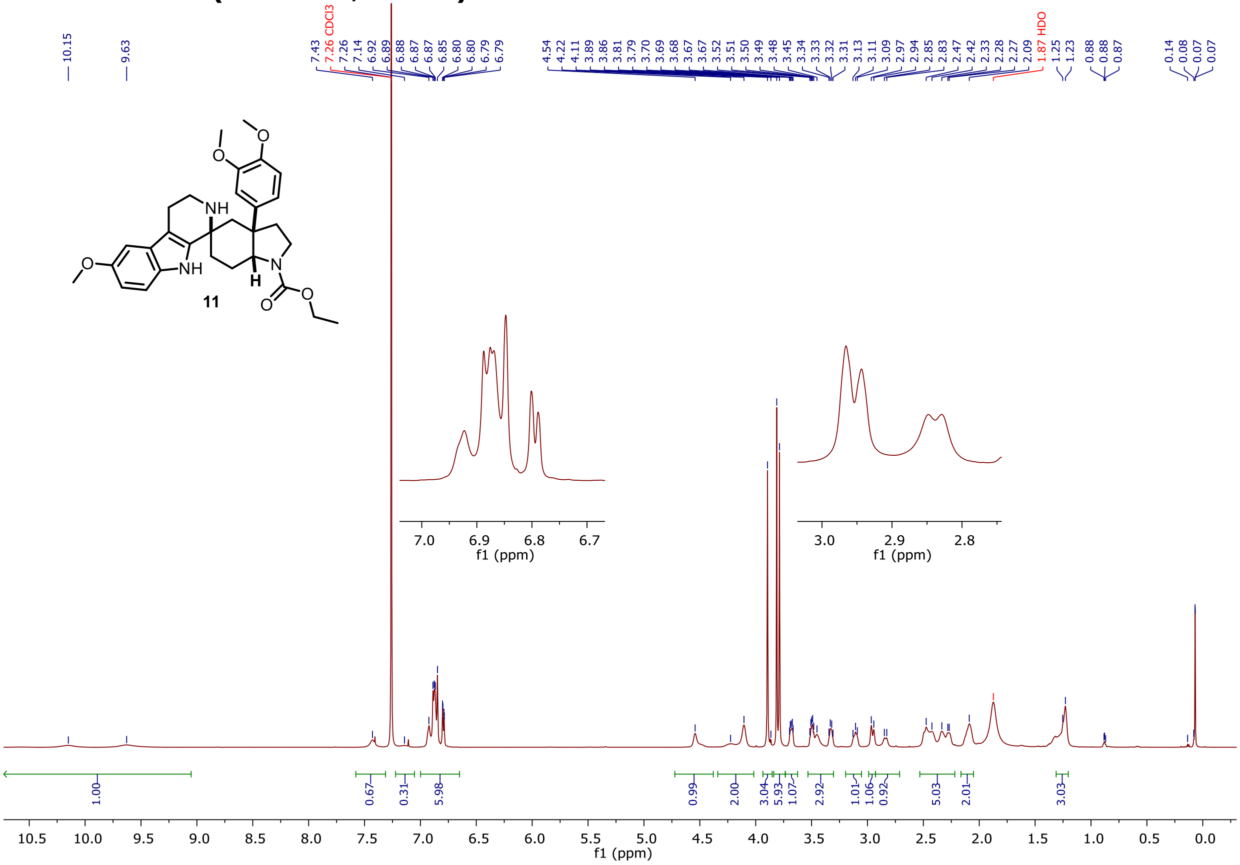

^{13}C-NMR of 11 (176 MHz, $CDCl_3$)

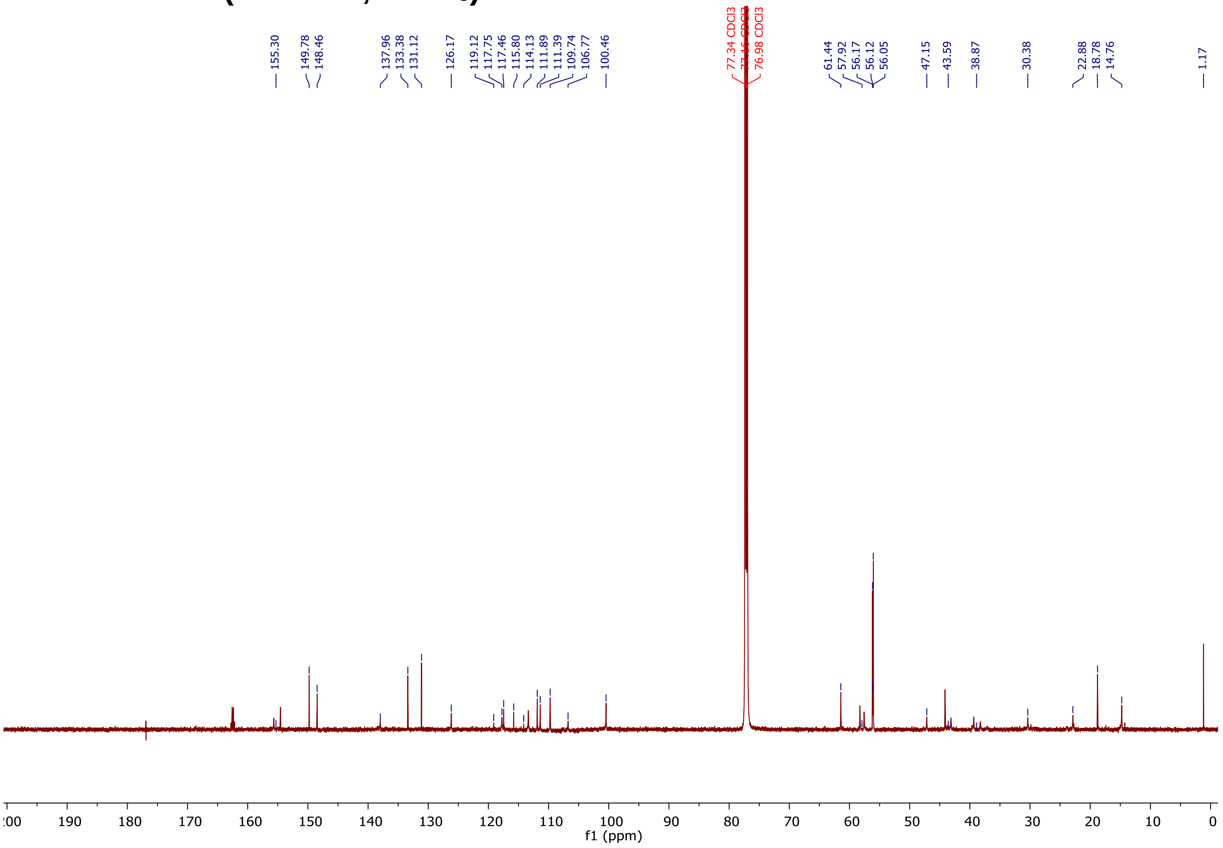

Supplement: Supplementary file 1 — Supplementary Material [file CMDC-21-e202501102-s001.pdf]
